# A Biocatalytic Platform for the Synthesis of Enantiopure Propargylic Alcohols and Amines

**DOI:** 10.1021/acs.orglett.2c01547

**Published:** 2022-06-07

**Authors:** Xianke Sang, Feifei Tong, Zhigang Zeng, Minghu Wu, Bo Yuan, Zhoutong Sun, Xiang Sheng, Ge Qu, Miguel Alcalde, Frank Hollmann, Wuyuan Zhang

**Affiliations:** † School of Nuclear Technology and Chemistry & Biology, Hubei University of Science and Technology, 88 Xianning Avenue, Xianning, Hubei 437100, China; ‡ Tianjin Institute of Industrial Biotechnology, Chinese Academy of Sciences, 32 West Seventh Avenue, Tianjin 300308, China; § Department of Biocatalysis, Institute of Catalysis, CSIC, 28049 Madrid, Spain; ∥ Department of Biotechnology, Delft University of Technology, van der Maasweg 9, 2629HZ Delft, The Netherlands

## Abstract

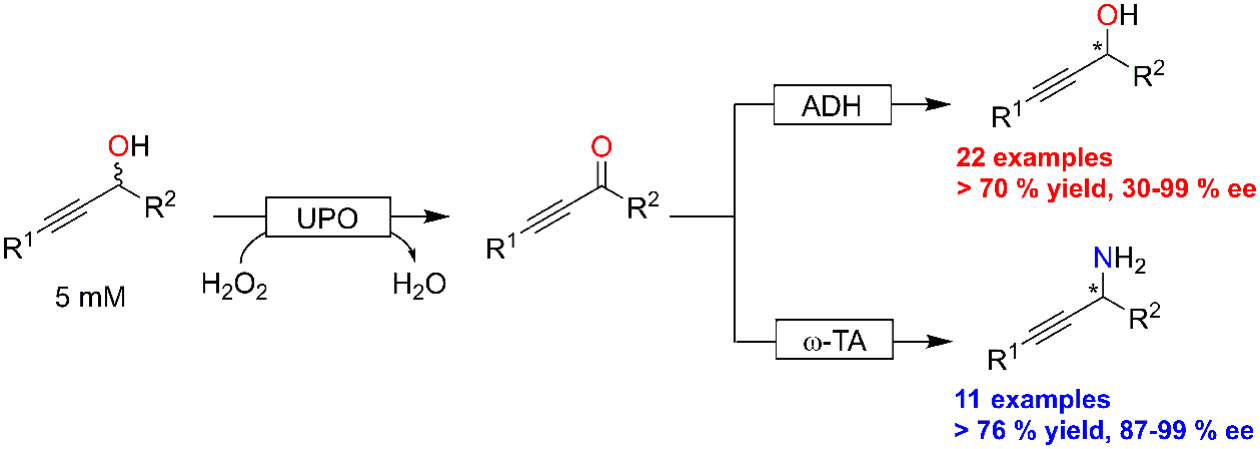

Propargylic alcohols and amines are versatile building blocks in organic synthesis. We demonstrate a straightforward enzymatic cascade to synthesize enantiomerically pure propargylic alcohols and amines from readily available racemic starting materials. In the first step, the peroxygenase from *Agrocybe aegerita* converted the racemic propargylic alcohols into the corresponding ketones, which then were converted into the enantiomerically pure alcohols using the (*R*)-selective alcohol dehydrogenase from *Lactobacillus kefir* or the (*S*)-selective alcohol dehydrogenase from *Thermoanaerobacter brokii*. Moreover, an enzymatic Mitsunobu-type conversion of the racemic alcohols into enantiomerically enriched propargylic amines using (*R*)-selective amine transaminase from *Aspergillus terreus* or (*S*)-selective amine transaminase from *Chromobacterium violaceum* was established. The one-pot two-step cascade reaction yielded a broad range of enantioenriched alcohol and amine products in 70–99% yield.

Functionalized alkynes, such as propargylic alcohols and amines, are versatile building blocks in organic chemistry.^1−3^ The presence of at least two functional groups predestines these compounds for a broad variety of chemical transformations, explaining their importance as building blocks in pharmaceutical and agrochemical chemistry. The natural antifungal agent Capillin, for example, is chemically synthesized via carbonyl oxidation of its pendant propargylic alcohols.^4^ Levonorgestrel and ethinylestradiol are common therapeutic hormones containing propargylic alcohols.^5^ Propargylic amines such as the monoamine oxidase-B inhibitors Pargyline^6^ and Rasagyline^7^ are used in the treatment of Parkinson’s disease and are of comparable importance.

Especially for the synthesis of APIs and fine chemicals, the optical purity of propargylic alcohols and amines is crucial. Therefore, it is not astonishing that a range of enantioselective syntheses starting from aldehydes^8−11^ or imines^12,13^ have been reported. Though conceptually elegant, these methods suffer from the need for comparably high (transition) metal catalyst loadings, posing a significant economic and environmental challenge. Very recently, Arnold and co-workers proposed a biocatalytic route to enantiomerically pure propargylic amines.^14^

Enantioselective reduction of propargylic ketones has been reported already in 2001 by Müller and co-workers.^21^ Using enantiocomplementary alcohol dehydrogenases, the authors demonstrated the synthesis of both enantiomers of propargylic alcohols. As racemic propargylic alcohols are more readily accessible, synthesis routes starting from racemic propargylic alcohols are desirable. Kinetic resolution approaches appear to be a practical route to enantiomerically pure products.^15−17^ Compared to the kinetic resolution strategy, deracemization (or dynamic kinetic resolution) intrinsically bears the advantage of theoretically yielding excellent conversion of the starting material into the desired product isomer (Scheme 1).

**Scheme 1:**
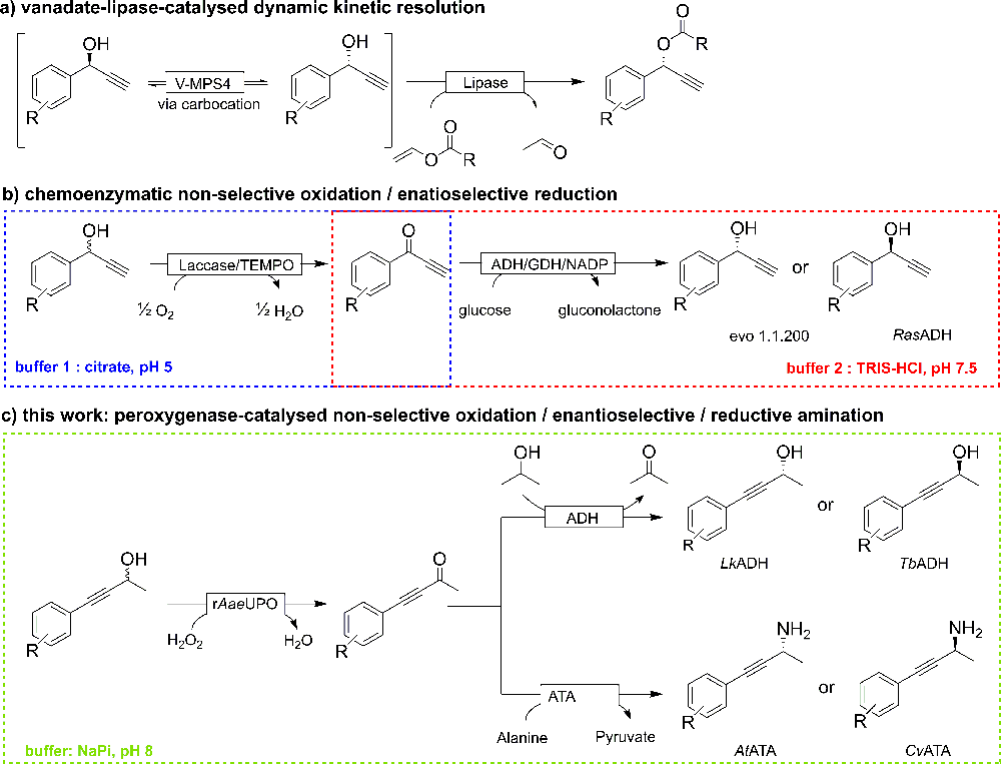
Synthetic Approaches to Obtain Enantiomerically Pure Propargylic Alcohols and Amines from Racemic Starting Materials

Chemoenzymatic approaches utilizing vanadium^18^ or TEMPO^19^ catalysts to enable the conversion of the racemic staring materials have been reported. The group around Lavandera and Gotor-Fernández, for example, used a laccase-TEMPO oxidation system to oxidize racemic propargylic alcohols into the corresponding ketones followed by enantioselective ADH-catalyzed reduction into the corresponding enantioenriched products.^19^ Unfortunately TEMPO, despite its popularity, is not a very efficient oxidation reagent, and catalyst loadings of up to 30 mol % are necessary in order to attain complete conversion of the alcohols to the corresponding ketones. Furthermore, the pH incompatibility of both reaction steps necessitated an intermittent buffer exchange.

Therefore, we set out to evaluate if peroxygenases may be more efficient catalysts for the first oxidation step. Though peroxygenases are mostly used for the oxyfunctionalization of (nonactivated) C–H-bonds^20,21^ recent experiments in our laboratories indicated that also the oxidation of alcohols to aldehydes/ketones may be an interesting application for peroxygenase catalysis.

As peroxygenase catalyst we chose the recombinant, evolved peroxygenase from *Agrocybe aegerita* (r*Aae*UPO, PaDa-I),^22,23^ which we recently produced on pilot scale.^24^ In a first set of experiments, we investigated the applicability of r*Aae*UPO for the complete oxidation of racemic 4-(4-fluorophenyl)but-3-yn-2-ol (**1a**, Figure 1). Previously, r*Aae*UPO has been reported as a highly enantioselective catalyst, especially for the hydroxylation of benzylic C–H-bonds;^25−27^ also the hydroxylation of but-1-yn-1-ylbenzene proceeded enantioselectively (85% ee). We therefore expected the r*Aae*UPO-catalyzed oxidation of racemic alcohol (**1a**) to result in a kinetic resolution and were very surprised observing that the oxidation of *rac*-(**1a**) occurred with low enantioselectivity (*E*-value of 3.6)^28^ (Figure 1), essentially converting both enantiomers.

**Figure 1:**
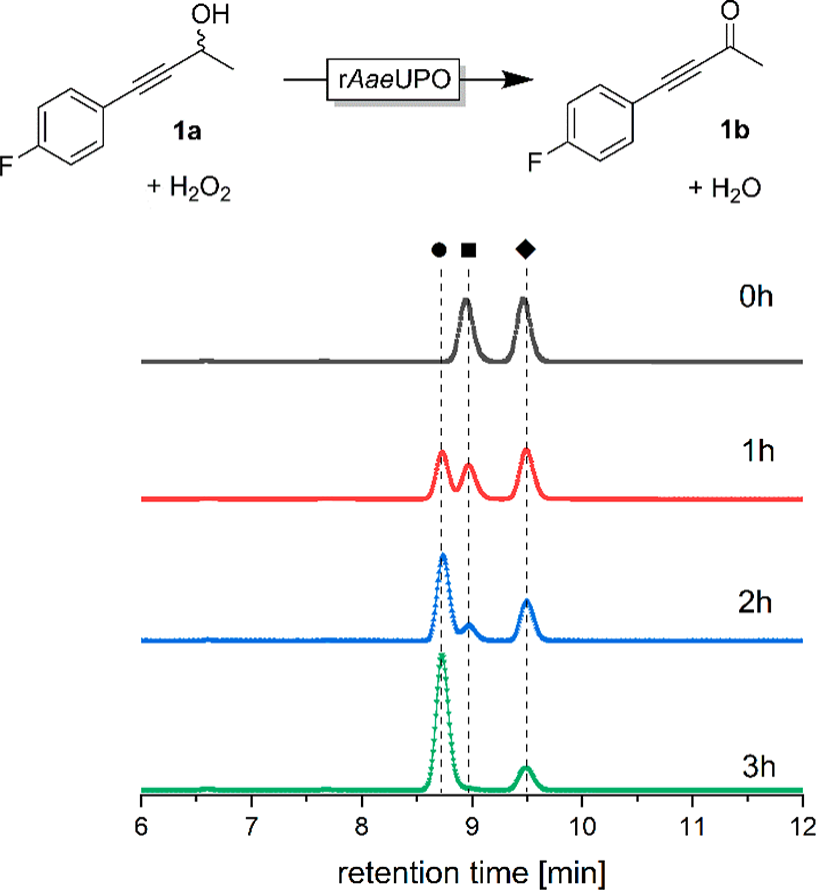
Oxidation of racemic 4-(4-fluorophenyl)but-3-yn-2-ol (**1a**) catalyzed by r*Aae*UPO monitored by chiral HPLC. Conditions: [**1a**] = 5 mM, [r*Aae*UPO] = 2 μM, [H_2_O_2_]_final_ = 10 mM added at 2 mM h^–1^, NaPi buffer (100 mM, pH 8), 30% (v/v) MeCN, 30 °C, 800 rpm. The retention times of (*R*)-**1a** (■), (*S*)-**1a** (◆), and the ketone product **1b** (●) were 7.8, 8.3, and 7.6 min, respectively. After 5 h, all starting material had been converted into the ketone (not shown).

To gain further insight into this unexpected low stereoselectivity of r*Aae*UPO toward the racemic alcohol, we modeled both enantiomers individually into the active site of r*Aae*UPO (Figure S1).^29,30^ It turned out that the binding affinity of both enantiomers was very similar, differing by only approximately 0.1 kcal × mol^–1^, suggesting that both enantiomers may be converted by r*Aae*UPO with comparable efficiency, thereby explaining the low enantioselectivity.

Complete oxidation of **1a** into **1b** was achieved within 5 h using 2 μM of r*Aae*UPO at a H_2_O_2_ addition rate of 2 mM h^–1^ (Figure S2). Control reactions in the absence of the biocatalyst under otherwise identical conditions did not yield in detectable conversion of any of these starting materials.

This procedure was extended to the oxidation of a range of racemic derivatives (Figure 2). With the exception of the substrates (**12a**–**14a**), all propargylic alcohols tested were oxidized into the corresponding ketones in good to excellent yields.

**Figure 2:**
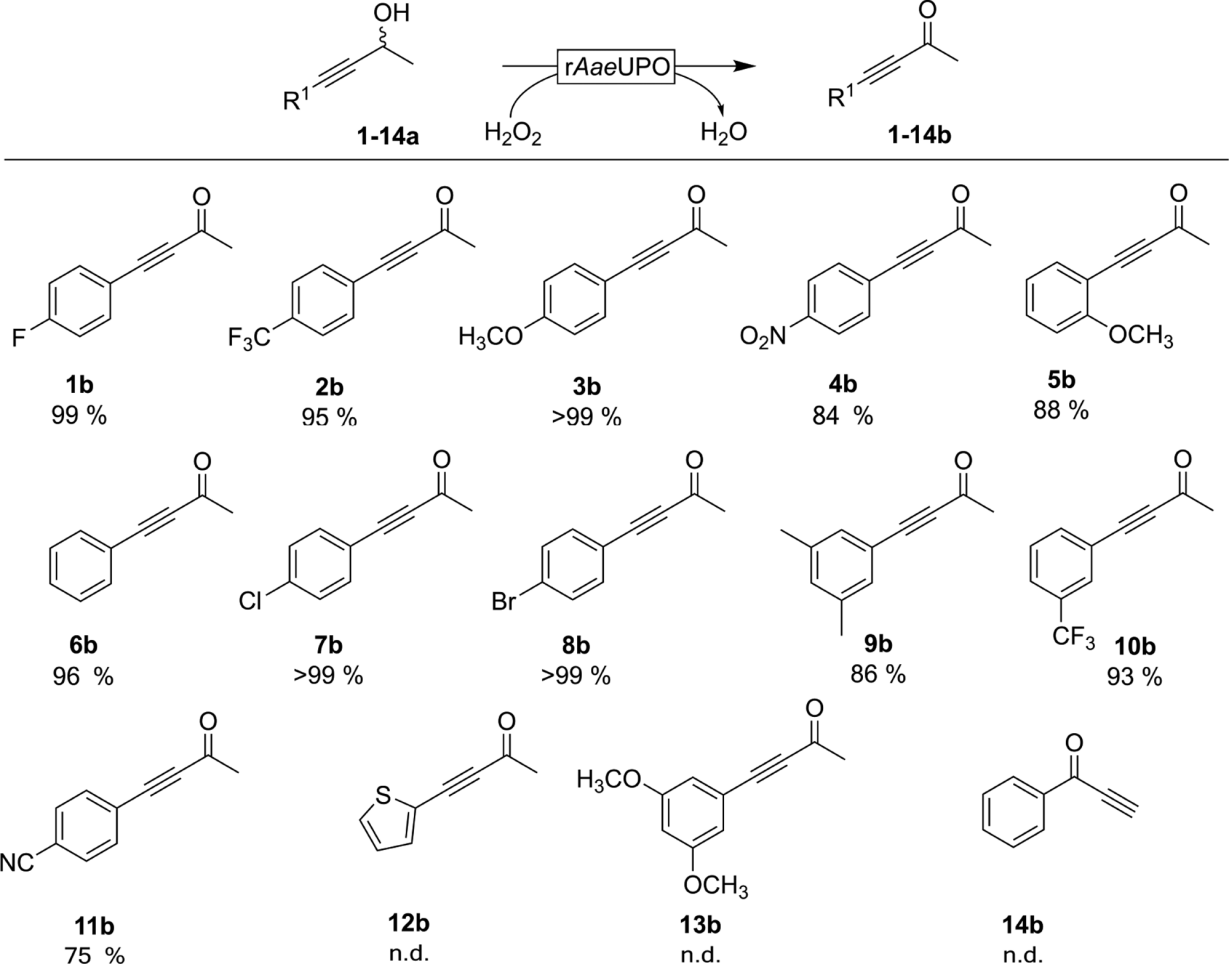
Peroxygenase catalyzed oxidation of racemic propargylic alcohols into the corresponding ketones. Conditions: [substrate] = 5 mM, [r*Aae*UPO] = 2 μM, [H_2_O_2_]_final_ = 10 mM added at 2 mM h^–1^, NaPi buffer (100 mM, pH 8), 30% (v/v) MeCN, 5 h, 30 °C, 800 rpm. N.D. = not detected. The GC yield was calculated via [ketone]_final_ × ([alcohol]_final_ + [ketone]_final_)^−1^.

Having established the r*Aae*UPO-catalyzed oxidation, we next investigated the enantioselective reduction of the ketone intermediate. For this, we chose the (*R*)-selective alcohol dehydrogenase from *Lactobacillus kefir* DSM 20587 (*Lk*ADH)^31^ as well as the (*S*)-selective ADH from *Thermoanaerobacter brokii* (*Tb*ADH).^32^ Both enzymes were prepared by recombinant expression in recombinant *E. coli* and used as lysed cells (obtained by treating the cell pellets with lysozyme and DNase I, see the SI for further details). Treating **1b** with *Tb*ADH or *Lk*ADH (in the presence of 5% v/v isopropanol for in situ regeneration of NADPH) gave the expected (*R*)-**1a** and (*S*)-**1a**, respectively, in high enantiomeric purity (Figure S3).

We therefore proceeded combining both reaction steps to attain the desired deracemization procedure. Preliminary experiments performing the cascade in a one-pot one-step fashion were not successful, resulting in a painfully slow deracemization of **1a**: after 7 h reaction time, the ee-value of (*R*)-**1a** had increased from 0% to 5.7%. Possibly catalase present in the ADH preparation (being a crude cell extract of the *E. coli* expression system) competed with r*Aae*UPO for the H_2_O_2_ added and thereby significantly decreased the oxidation rate of the first cascade step. Therefore, we drew our attention to a one-pot two-step procedure in which the r*Aae*UPO-catalyzed oxidation of racemic propargylic alcohols was followed by the enantioselective, ADH-catalyzed reduction. A representative time course is shown in Figure 3.

**Figure 3:**
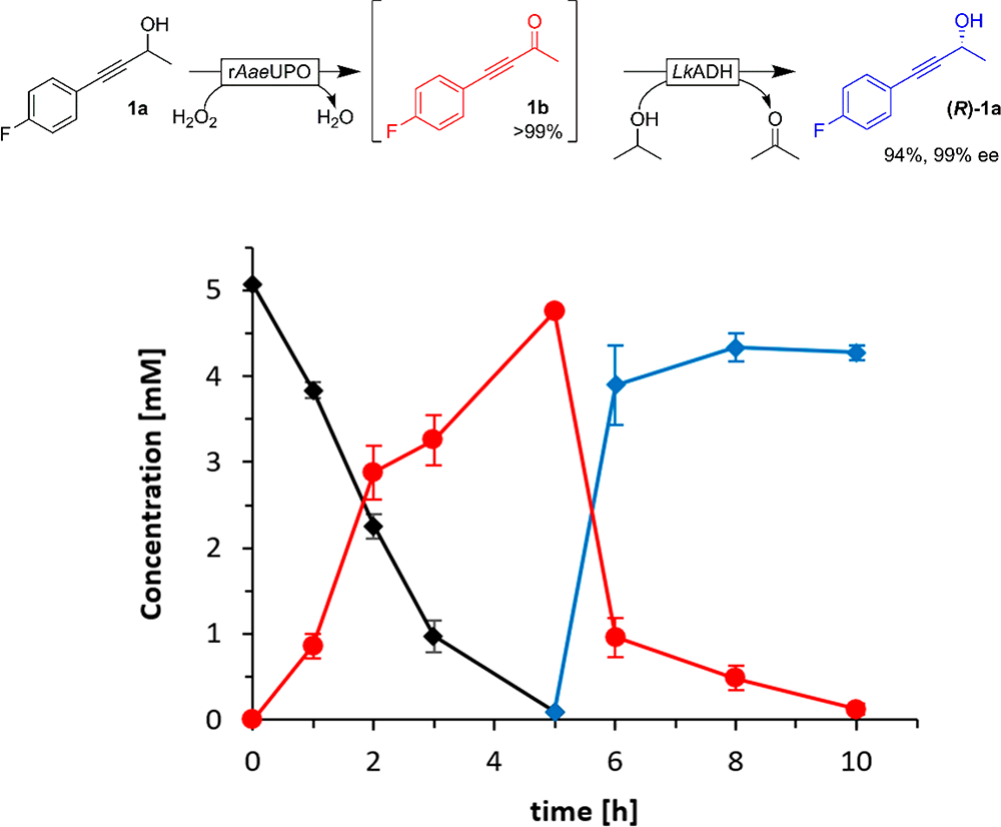
Representative time course of a one-pot two-step deracemization (**1a** (black ◆), **1b** (red ●), (*R*)-**1a** (blue ◆)). The r*Aae*UPO-catalyzed oxidation step was performed first, followed by addition of the ADH, isopropanol after 5 h. Conditions: [**1a**] = 5 mM, [r*Aae*UPO] = 2 μM; [H_2_O_2_]_final_ = 10 mM added at 2 mM h^–1^ (for 5 h), [*Lk*ADH] = 60 μM, NaPi buffer (100 mM, pH 8), 30% (v/v) MeCN, 5% isopropanol, [lysozyme] = 1 mg mL^–1^, [DNase I] = 6 U mL^–1^, 30 °C, 800 rpm.

Almost complete conversion of the racemic starting material (**1a**) into the ketone (**1b**) was achieved within 5 h. After addition of, e.g., *Lk*ADH and 5% (v/v) of isopropanol (serving as sacrificial reductant for the *Lk*ADH-catalyzed reduction reaction), smooth reduction into (*R*)-**1a** (94% yield and 99% ee) was observed. Similarly, using *Tb*ADH the corresponding (*S*)-**1a** was obtained in 77% yield and 98% ee (Figure S4–S6).

Encouraged by these results, we further synthesized a range of racemic propargylic alcohols (Figure S7–S45) and subjected them to the bienzymatic deracemization cascade (Figure 4). Depending on the substitution pattern of the starting material, the deracemization reaction proceeded between mediocre to excellent yields and optical purities of the products (Figure S46–S75). The ADH-selectivities were as expected with the notable exception of the *Tb*ADH-catalyzed reduction of **10b**, which resulted in the formation of the (*R*)-alcohol (*R*)-**10a** instead of the expected (*S*)-**10a**.

**Figure 4:**
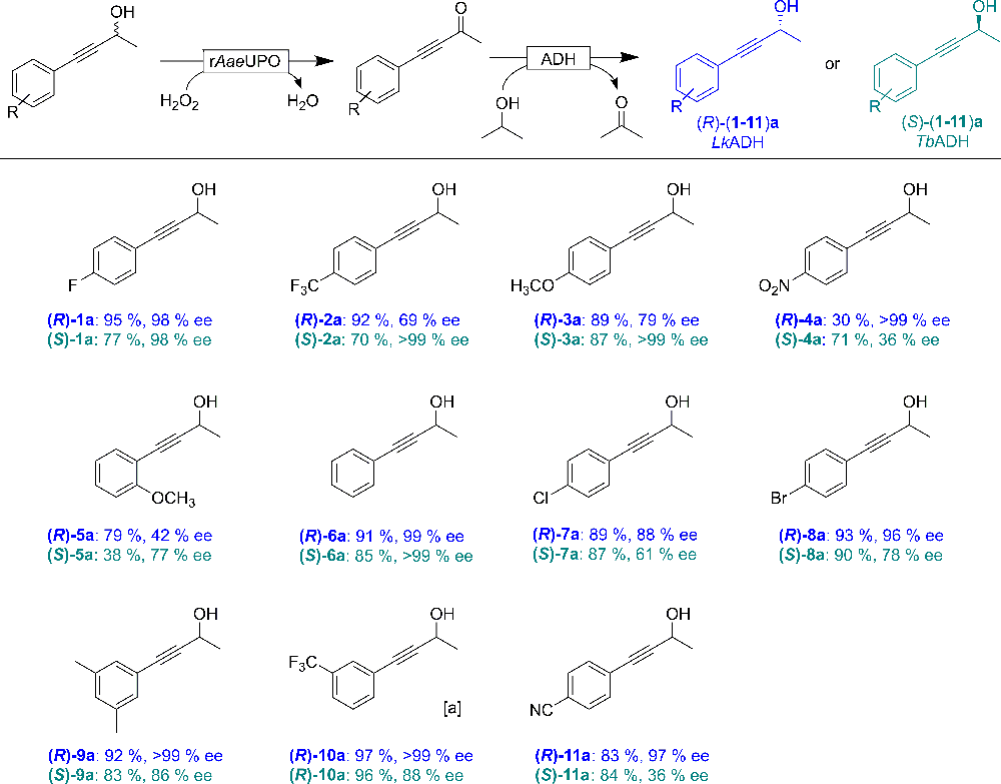
Biocatalytic cascade reaction for the synthesis of enantiomerically pure propargylic alcohols. Reaction conditions: [**1a**–**11a**] = 5 mM, [r*Aae*UPO] = 2 μM, [H_2_O_2_]_final_ = 10 mM added at 2 mM h^–1^, [*Lk*ADH] = 60 μM, or [*Tb*ADH] = 25 μM, NaPi buffer (100 mM, pH 8), 30% (v/v) MeCN, 5% isopropanol, [lysozyme] = 1 mg mL^–1^, [DNase I] = 6 U mL^–1^, 30 °C, 5 h, 800 rpm. n.d. = Not detected. ^*a*^Note that using *Tb*ADH yielded in formation of the (*R*)-alcohol.

Overall, the bienzymatic deracemization of a range of propargylic alcohols was established. The catalytic performance of the individual catalysts in terms of turnover numbers (TON, [product]_final_ × [catalysts]^−1^) was calculated to be 2500, 80, and 190 for r*Aae*UPO, *Lk*ADH, *Tb*ADH, respectively. The concentration of the nicotinamide cofactor (endogenously contained in the *E. coli* crude extract) can be estimated using a NADP content of approximately 0.51 μmol × g_wet *E. coli*_^–1^ to be around 51 μM,^33,34^ translating into an approximate TON_NADP_ around 100.

Next, we enlarged the bienzymatic deracemization concept with a reductive amination step yielding an enantioselective, biocatalytic variant of the Mitsunobu reaction.^35−37^ For this, we simply substituted the previously used ADHs in the sequential cascade by the (*R*)-selective amine transaminase from *Aspergillus terreus* (*At*ATA)^38^ or the (*S*)-selective amine transaminase from *Chromobacterium violaceum* DSM30191 (*Cv*ATA).^39^ Again, the reaction was realized as one-pot two-step procedure (Figure 5). A representative time course converting *rac*-**6a** into (*R*)-**6c** is shown in Figure S76. Under partially optimized reaction conditions (Figure S77–S79) a range of enantiomerically pure (*R*)- and (*S*)-propargylic amines could be obtained in reasonable to excellent yields and enantiomeric excess from the corresponding racemic alcohols (see experimental details in the SI, Figures S80–S100 and Figures S101–S108). The TONs observed for *At*ATA and *Cv*ATA were 62 and 122.

**Figure 5:**
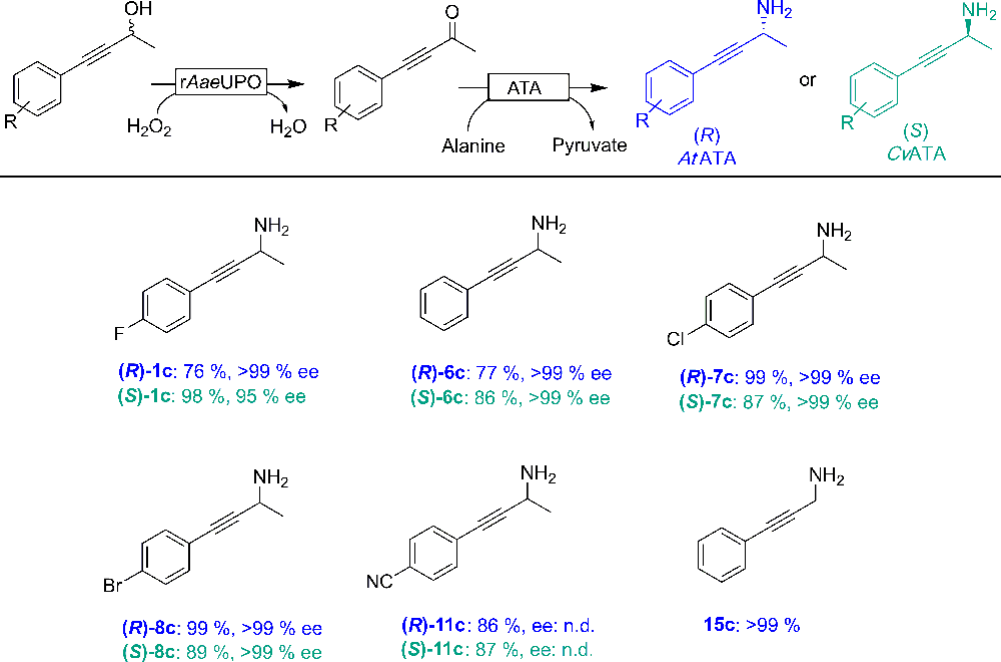
Biocatalytic cascade reaction for the synthesis of enantiomerically pure propargylic amines from racemic alcohols. Reaction conditions: [substrate] = 5 mM, [r*Aae*UPO] = 2 μM, [H_2_O_2_] = 2 mM h^–1^ for 5 h, [H_2_O_2_]_final_ = 10 mM, [*At*ATA] = 80 μM or [*Cv*ATA] = 40 μM, [PLP] = 0.1 mM, [d/l-alanine] = 1 M, NaPi buffer (100 mM, pH 8), 30% (v/v) MeCN, 30 °C, 800 rpm, 24 h. n.d. = not determined.

Finally, we performed a semipreparative scale synthesis. Both propargylic alcohol and amine were performed at 3 mmol scale (20 mM of **1a** in 150 mL a representative time course is shown in Figure S109). The peroxygenase enabled complete oxidation of **1a** in the first step. The overall cascade reaction gave (*S*)-**1a** and (*R*)-**1c** in 76% (91.1% ee) and 19.5% (>99% ee) isolated yield, respectively (Figure S110–115). The low isolated yield of (*R*)-**1c** was due to issues with the chromatographic purification.

In summary, we have established a catalytic platform transforming readily accessible racemic propargylic alcohols into enantiomerically pure propargylic alcohols and amines. We are convinced that the simplicity of the procedure will provide preparative organic and medicinal chemists with a practical tool for their synthesis planning. Apparently, the catalytic performance of the enzymes used has to be increased considerably to attain economic feasibility. We are, however, convinced that further reaction engineering measures can increase the TONs considerably. For example, external addition of the ADH-cofactor may accelerate the ADH-catalyzed reduction reaction. Also the efficiency of the reductive amination can be improved upon application of suitable coupled reactions to shift the equilibrium.^40^ Efforts broadening the substrate scope (e.g., for starting materials **12a**–**14a**) via engineered r*Aae*UPO variants, expanding the amine product scope and increasing the substrate loading to preparatively relevant scales, are currently ongoing in our laboratories.

## Supporting Information Available

Experimental details, biocatalytic reactions, ^1^H and ^13^C NMR, GC and HPLC chromatograms, and GC-MS data (PDF)
(*R*)-**1a** docked into the enzyme (PDB)
(*S*)-**1a** docked into the enzyme (PDB)

## Supplementary Material

ol2c01547_si_001.pdf

ol2c01547_si_002.pdb

ol2c01547_si_003.pdb
