# Supplementary material for: A Biocatalytic Platform for the Synthesis of Enantiopure Propargylic Alcohols and Amines
Source: Org Lett. 2022 Jun 7;24(23):4252–7. doi: 10.1021/acs.orglett.2c01547 (PMC9208015; doi:10.1021/acs.orglett.2c01547)
Supplement: Supplementary file 1 — ol2c01547_si_001.pdf [file ol2c01547_si_001.pdf]

Supporting Information

A Biocatalytic Platform for the Synthesis of Enantiopure Propargylic Alcohols and Amines

Xianke Sang,[a,b] Feifei Tong,[b] Zhigang Zeng,[a,f]* Minghu Wu,[a,f] Bo Yuan,[b] Zhoutong Sun,[b,e] Xiang Sheng,[b,e] Ge Qu,[b,e] Miguel Alcalde,[c] Frank Hollmann,[d]* Wuyuan Zhang[b,e]*

[a] School of Nuclear Technology and Chemistry & Biology, Hubei University of Science and Technology, Xianning, Hubei 437100

[b] Tianjin Institute of Industrial Biotechnology, Chinese Academy of Sciences, 32 West 7th Avenue, Tianjin 300308, China

[c] Department of Biotechnology, Delft University of Technology, Van der Maasweg 9, 2629HZ Delft, The Netherlands

[d] Department of Biocatalysis, Institute of Catalysis, CSIC, 28049 Madrid, Spain

[e] National Center of Technology Innovation for Synthetic Biology, 32 West 7th Avenue, Tianjin 300308, China

[f] Hubei Key Laboratory of Radiation Chemistry and Functional Materials, 88 Xianning Avenue, Xianning, Hubei 437100, China

Email: zzgang2012@hotmail.com; f.hollmann@tudelft.nl; zhangwy@tib.cas.cn

Materials

All chemicals were purchased from Sigma-Aldrich, Bide Pharmatech Ltd, Macklin, Energy-Chemical or Alfa-Aesar with the highest purity available and used without further treatment.

Cloning and expression of alcohol dehydrogenases

Genes for the alcohol dehydrogenases (ADH) from *Lactobacillus kefiri* DSM 20587 (GeneBank: AY267012.1, *Lk*ADH)[1] and *Thermoanaerobacter brockii* (GeneBank: WP_014063649.1, *Tb*ADH)[2] were incorporated into pET24a between restriction sites of *Nde* I and *Xho* I and expressed in *E. coli* BL21(DE3) cells, respectively. The cells were cultivated in 5 mL LB medium containing 50 μg/mL kanamycin at 37 °C and 220 rpm for 6-8 h. Subsequently 1 %(v/v) seed culture was transferred into TB medium containing 50 μg/mL kanamycin at 37 °C and 220 rpm. Induction for protein expression started by addition of 0.1 mM isopropyl β-D-1-thiogalactopyranoside (IPTG) when OD_{600} reached 0.6-0.8. Then 0.1 mM zinc ions were added to the culture. The cultivation continued for 16 h at 20 °C and 220 rpm. The cells were harvested by centrifugation at 4 °C and 4000 rpm for 10 min, washed and resuspended with PBK buffer (100 mM, pH 7.4), stored at -20 °C for the preparation. SDS-PAGE was run with the crude enzymes after suspending and disrupting the cells in PBK buffer.

Cloning and expression of amine transaminase

The (*R*)-selective amine transaminase from *Aspergillus terreus* (*At*ATA) was prepared according to the reported procedures[3]. ATA plasmid (2 μL) was added to the *E. Coli* BL21 (DE3) competent cells, and electroporation was performed. Sterilized LB medium (900 μL) was added to the cells and subsequently resuscitated at 37 °C and 200 rpm for 1 hour. Cells were harvested by centrifugation at 4000 rpm for 2 min to collect the bacteria and the supernatant was discarded. After fully resuspended, 100 μL of the solution was taken and spread on the LB agar plate supplemented with 100 μg/mL ampicillin, then incubated overnight at 37 °C, 200 rpm. Single colony was picked and inoculated, the seed culture was incubated overnight at 37 °C, 200 rpm in 5 mL LB medium supplemented with 100 μg/mL ampicillin. 1 mL of the seed solution was then inoculated into 100 mL TB medium (including 10 mL sterilized TB buffer) supplemented with 100 μg/mL ampicillin and incubated at 37 °C and 200 rpm for 3-4 hours until OD_{600} reached 0.6-0.8. Induction was performed by addition of IPTG (0.1 mmol/L final concentration) 100 μL at 20 °C for 15 h. The cells were then harvested by centrifugation at 4000 rpm and 4 °C for 20 min. The supernatant was discarded, and the cells were resuspended with addition of 5 mL phosphate

buffer (50 mmol/L pH 7.4). The cell pallet was then washed by phosphate buffer (10 mL/g cell pallet). Sonification was then performed to disrupt the cells with the programmed 4 s on, 3 s off, 20 min in total. After centrifugation the cell free extract (CFE) was obtained and applied for (*R*)-propargylic amine synthesis.

Similar procedures were taken to prepare the (*S*)-selective amine transaminase from *Chromobacterium violaceum* DSM30191 (*Cv*ATA) as described by Kaulmann et al[4]. After the centrifugation the cell free extract (CFE) was obtained and applied for (*S*)-propargylic amine synthesis.

Oxidation of racemic propargylic alcohols

Taking the oxidation of **1** as an example, the alcohol oxidation catalyzed by peroxygenase was performed according to the following procedures: firstly, 5 mM of substrate, 1 µM r*Aae*UPO, and 30 % of acetonitrile (v/v) in 950 µL NaPi buffer (50 mM) were added to a 2 mL vial. Then, H_2O_2 from a 200 mM stock solution was added slowly by a syringe pump at a rate of 10 µL h^{-1}. The total concentration of H_2O_2 was 10 mM (2 equiv. of the substrate). The solution was mixed in a thermal shaker at 30 ^oC, 800 rpm. Aliquots were withdrawn at intervals. The reaction mixture was extracted using ethyl acetate (extraction ratio: 1 : 2) containing dodecane as the internal standard (5 mM) and dried over Na_2SO_4. The samples were then analyzed by gas chromatography (GC).

Biocatalytic cascade synthesis of enantiopure propargylic alcohols

The typical procedures for the envisioned enantiopure propargylic alcohol synthesis started with the alcohol oxidation by peroxygenase following the above-mentioned procedures. The reaction conditions after optimization were: [4-(4-fluorophenyl)-3-butyn-2-ol] = 5 mM, [H_2O_2] = 10 mM, [H_2O_2 dose rate] = 2 mM h^{-1}, [*rAae*UPO]= 2 µM, pH 8.0 (phosphate buffer, 100 mM), 30 % of acetonitrile. The reaction vial was sealed and incubated in a thermal shaker at 30 °C, 800 rpm. After 5 hours ,4-(4-fluorophenyl)-3-butyn-2-ol was converted into 4-para-fluorophenyl-3-butyn-2-one. Then, the alcohol dehydrogenase, isopropanol, lysozyme and Dnase I were added as the second step. The final conditions were: [ADH] = 60 µM (0.1 g mL^{-1} wet cell), isopropanol = 5 % v/v, [lysozyme] = 1 mg mL^{-1}, [Dnase I] = 6 U mL^{-1}, 5 h, 30 ^oC, 800 rpm. At intervals, aliquots were withdrawn. The reaction mixture was extracted using ethyl acetate (extraction ratio: 1:2) containing dodecane as internal standard (5 mM) and dried over Na_2SO_4. The samples were then analyzed by gas chromatography (GC). To determine the

enantiomeric excess, normal phase HPLC was used.

Biocatalytic cascade synthesis of enantiopure propargylic amines

The procedures herein followed those in the cascade synthesis of enantiopure propargylic alcohols. In the second step, the ATA, PLP and D-alanine (solid) were added. The final reaction conditions were: [ATA] = 0.1 g mL^{-1} wet cell, [PLP] = 0.1 mM, [D-alanine] = 1 M, 5 h, 30 ^{o}C, 800 rpm. Aliquots were withdrawn at intervals. The reaction mixture was extracted using ethyl acetate (extraction ratio: 1 : 2) containing dodecane as the internal standard (5 mM) and dried over Na_2SO_4. The samples were then analyzed by gas chromatography (GC). To determine the enantiomeric excess, HPLC in normal phase was used.

Computational methods

The structure of Solo-UPO used in the molecular docking is constructed on the basis of the crystal structure of *Aae*UPO (PDB ID: 5OXU). The grid map with 60×60×60 points and a grid-point spacing of 0.375 Å was centered at the Fe atom by AutoGrid 4.2.[5] Then, AutoDock 4.2 was used for the docking simulations. The best-scored docking pose was acquired from each docking for the analysis.

Preparative scale reactions

Oxidation of racemic propargylic alcohols in preparative scale

The preparative scale synthesis of (*S*)-**1a** and (*R*)-**1c** was performed starting from 3.2 mmol of substrate **1a** in ca. 150 mL reaction volume.

Specifically, the alcohol oxidation of **1a** catalyzed by peroxygenase was performed by adding the substrate, r*Aae*UPO, and the co-solvent into NaPi buffer placed in a round bottom flask. In this step, the final solution contained 21 mM of **1a** (0.52 g, 3.2 mmol, 1 equiv), 4 µM of r*Aae*UPO, 100 mL of NaPi buffer (100 mM, pH 8) and 45 mL of acetonitrile (30 % v/v). Subsequently, H_2O_2 from a 200 mM stock solution was added slowly by a syringe pump at a rate of 1.5 mL h^{-1}. The final concentration of H_2O_2 was 30 mM. The solution was stirred on a magnetic stirrer. At intervals, aliquots were withdrawn at 1 h, 3 h, 12 h and 16 h. At 12 h, to the reaction solution was added the fresh r*Aae*UPO (final concentration: 1 µM). The overall conditions of **1a** oxidation were: **1a** (0.52 g, 3.2 mmol, 1 equiv), [r*Aae*UPO] = 5 µM,

$[H_2O_2]_{final}$ = 30 mM at 2 mM h^{-1} rate, NaPi buffer (100 mM, pH 8), 30 % (v/v) MeCN, 150 mL reaction volume, 30 °C, 200 rpm. The reactions were essentially complete (no starting material detectable via GC analysis. The ketone product was not isolated and the reaction mixtures (150 mL each) were used 'as is' for further transformations (i.e. reduction to (*S*)-**1a** and (*R*)-**1c**, respectively, vide infra).

Biocatalytic cascade of enantiopure propargylic alcohols in preparative scale

When the-above mentioned **1a** oxidation to **1b** was completed, the reaction ingredients for *Tb*ADH catalyzed reduction were directly added to the reaction mixture. After 3 h, a portion of fresh *Tb*ADH was added again. The solution was stirred on a magnetic stirrer at 30 °C, 200 rpm. The final condition was: [substrate **1b**] = 21 mM (0.52 g, 3.2 mmol, 1 equiv), [*Tb*ADH] = 36 µM, NaPi buffer (100 mM, pH 8), 5 % isopropanol (7.5 mL), [lysozyme] = 1 mg mL^{-1}, [Dnase I] = 6 U mL^{-1}, 30 °C, 800 rpm. At the end of the reactions, the reaction mixture was extracted using ethyl acetate for 3 times, dried over Na_2SO_4. The organic phase was evaporated under vacuum. 395 mg (2.4 mmol) of the final product (*S*)-**1a** with 91.1 % ee was obtained, corresponding to a 75 % isolated yield.

Biocatalytic cascade of enantiopure propargylic amines in preparative scale

When the-above mentioned **1a** oxidation to **1b** was completed, the reaction ingredients for *At*ATA catalyzed reduction were directly added to the reaction mixture. The final reaction conditions of the reductive amination were: [substrate **1b**] = 21 mM (0.52 g, 3.2 mmol, 1 equiv), [cell free extract of *At*ATA] = 60 µM, [PLP] = 0.1 mM, D-alanine = 1 M (13.35 g, 0.15 mol, 47 equiv), NaPi buffer (100 mM, pH 8), 30 % (v/v) MeCN, 30 °C, 200 rpm, 150 mL reaction volume. At the end of the reactions, the reaction mixture was extracted using ethyl acetate for 3 times, dried over Na_2SO_4. The organic phase was evaporated under vacuum. 101.6 mg (0.62 mmol) of the final product (*R*)-**1c** with >99 % ee was obtained, corresponding to 19.5 % isolated yield.

Synthesis of racemic propargylic alcohols

General procedures were adapted from literatures[6-8]: to a round bottom flask was added $Pd(PPh_3)_2Cl_2$ and CuI. The flask was purged with N_2 and Et_3N was added via a syringe under N_2 protection. The aryl iodide was then added and followed by the addition of 3-butyne-2-ol. The reaction mixture was stirred at room temperature or heated when necessary, and the reaction progress was monitored by thin-layer chromatography (TLC). Upon completion, the crude mixture was filtered through a filter

paper on top of a separation funnel, the solid residue was washed with ethyl acetate. The combined organic phase was concentrated under reduced pressure. Purification via flash column chromatography yielded the desired aryl propargyl alcohols.

General procedures A: $Pd(PPh_3)_2Cl_2$ (0.02 mmol, 0.01 equiv) and CuI (0.04 mmol, 0.02 equiv) were suspended in Et_3N (16 mL) under N_2. The substituted iodobenzene (2 mmol, 1 equiv) was added, followed by the addition of the alcohol (2.2 mmol 1.1 equiv). The reaction was stirred at room temperature for 10 hours. Subsequent workup and column chromatography (hexane : EtOAc = 10 : 1) yielded the final compound as an orangish solid.

4-(4-fluorophenyl)-3-butyn-2-ol (1a)

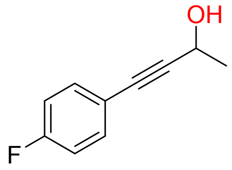

4-(4-fluorophenyl)-3-butyn-2-ol was prepared following **General Procedure A** starting with 1-fluoro-4-iodobenzene (2 mmol, 490 mg) and 3-butyne-2-ol (2.2 mmol,154 mg). After purification by column chromatography the final compound was obtained as a yellow solid in 83 % yield (272 mg). ^{1}H NMR (400 MHz, $CDCl_3$) δ 7.43 – 7.38 (m, 2H), 7.03 – 6.97 (m, 2H), 4.77 – 4.71 (m, 1H), 1.97 – 1.94 (m, 1H), 1.55 (d, J = 6.6 Hz, 3H). ^{13}C NMR (100 MHz, $CDCl_3$) δ 162.6 (d, J = 249.5 Hz), 133.6 (d, J = 8.4 Hz), 118.7 (d, J = 3.5 Hz), 115.6 (d, J = 22.2 Hz), 90.7 (d, J = 1.5 Hz), 83.0, 58.9, 24.4.

4-(4-(Trifluoromethyl)phenyl)but-3-yn-2-ol (2a)

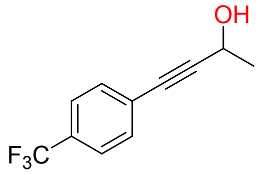

4-(4-(Trifluoromethyl)phenyl)but-3-yn-2-ol was prepared following **General Procedure A** starting with 1-iodo-4-(trifluoromethyl)benzene (2 mmol, 590 mg) and 3-butyne -2-ol (2.2 mmol, 154 mg). After purification by column chromatography the final compound was obtained as an orange oil in 71 % yield (304 mg). ^{1}H NMR (400 MHz, $CDCl_3$) δ 7.52 – 7.50 (m, 4H), 4.80 – 4.74 (m, 1H), 2.13 (d, J = 5.3 Hz, 1H),

1.57 (d, J = 6.6 Hz, 3H). ^{13}C NMR (100 MHz, $CDCl_3$) δ 131.9, 130.2 (d, J = 32.6 Hz), 126.5 (d, J = 1.6 Hz), 125.3 (q, J = 3.9 Hz), 122.6, 93.4, 82.8, 58.8, 24.3.

4-(4-Methoxyphenyl)but-3-yn-2-ol (3a)

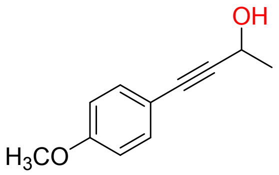

4-(4-Methoxyphenyl)but-3-yn-2-ol was prepared following **General Procedure A** starting with 1-iodo-4-methoxybenzene (2 mmol, 516 mg) and 3-butyne-2-ol (2.2 mmol, 154 mg). After purification by column chromatography the final compound was obtained as an orange oil in 73 % yield (257 mg). ^{1}H NMR (400 MHz, $CDCl_3$) δ 7.37 – 7.34 (m, 2H), 6.84 – 6.81 (m, 2H), 4.77 – 4.71 (m, 1H), 3.80 (s, 3H), 2.14 (d, J = 5.1 Hz, 1H), 1.54 (d, J = 6.6 Hz, 3H).^{13}C NMR (100 MHz, $CDCl_3$) δ 159.7, 133.2, 114.7, 114.0, 89.7, 84.0, 59.0, 55.3, 24.6.

4-(4'-nitrophenyl)-3-butyn-2-ol (4a)

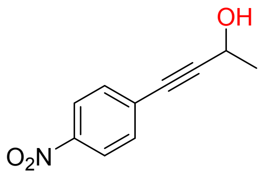

4-(4'-nitrophenyl)-3-butyn-2-ol was prepared following **General Procedure A** starting with 1-iodo-4-nitrobenzene (2 mmol, 546 mg) and 3-butyne-2-ol (2.2 mmol, 154 mg). After purification by column chromatography the final compound was obtained as an orange solid in 63 % yield (241 mg). ^{1}H NMR (400 MHz, DMSO-d_6) δ 8.25 – 8.22 (m, 2H), 7.71 – 7.67 (m, 2H), 5.65 (d, J = 5.4 Hz, 1H), 4.70 – 4.63 (m, 1H), 1.42 (d, J = 6.6 Hz, 3H). ^{13}C NMR (100 MHz, DMSO-d_6) δ 147.2, 133.0, 129.8, 124.3, 99.2, 81.2, 57.2, 24.8.

4-(2-Methoxyphenyl)but-3-yn-2-ol (5a)

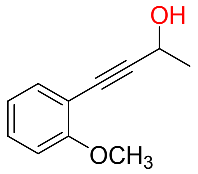

4-(2-Methoxyphenyl)but-3-yn-2-ol was prepared following **General Procedure A** starting with 1-iodo-2-methoxybenzene (2 mmol, 516 mg) and 3-butyn-2-ol (2.2 mmol, 154 mg). The reaction was heated to 70 °C for 17 hours. After purification by column chromatography the final compound was obtained as an orange oil in 58 % yield (204 mg). ^{1}H NMR (400 MHz, $CDCl_3$) δ 7.44– 7.42 (m, 1H), 7.35 – 7.31 (m, 1H), 6.96 – 6.90 (m, 2H), 4.88 – 4.82 (m, 1H), 3.92 (s, 3H), 2.44 (d, J = 4.8 Hz, 1H), 1.61 (d, J = 6.6 Hz, 3H). ^{13}C NMR (100 MHz, $CDCl_3$) δ 159.9, 133.8, 129.9, 120.5, 111.8, 110.7, 95.33, 80.3, 59.0, 55.8, 24.4.

4-Phenylbut-3-yn-2-ol (6a)

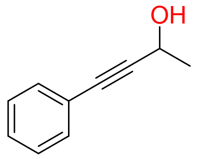

4-Phenylbut-3-yn-2-ol was prepared following **General Procedure A** starting with Iodobenzene (2 mmol, 454 mg) and 3-butyne-2-ol (2.2 mmol, 154 mg). After purification by column chromatography the final compound was obtained as an orange oil in 81 % yield (237 mg). ^{1}H NMR (400 MHz, $CDCl_3$) δ 7.47 – 7.45 (m, 2H), 7.36 – 7.32 (m, 3H), 4.82 – 4.76 (m, 1H), 2.06 (d, J = 5.3 Hz, 1H), 1.59 (d, J = 6.6 Hz, 3H). ^{13}C NMR (100 MHz, $CDCl_3$) δ 131.9, 128.6, 128.5, 122.8, 91.2, 84.3, 59.1, 24.6.

4-(4-chlorophenyl)-3-butyn-2-ol (7a)

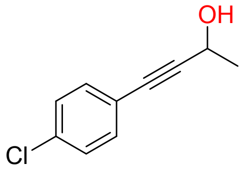

4-(4-chlorophenyl)-3-butyn-2-ol was prepared following **General Procedure A** starting with 1-chloro-4-iodobenzene (2 mmol, 522 mg) and 3-butyne-2-ol (2.2 mmol, 154 mg). After purification by column chromatography the final compound was obtained as a yellow solid in 66 % yield (238 mg). ^{1}H NMR (400 MHz, $CDCl_3$) δ 7.41 – 7.38 (m, 2H), 7.34 – 7.31 (m, 2H), 4.82 – 4.76 (m, 1H), 2.09 (d, J = 5.3

Hz, 1H), 1.60 (d, J = 6.6 Hz, 3H). ^{13}C NMR (100 MHz, $CDCl_3$) δ 134.5, 132.9, 128.7, 121.1, 92.0, 83.0, 58.9, 24.4.

4-(4-bromophenyl)-3-butyn-2-ol (8a)

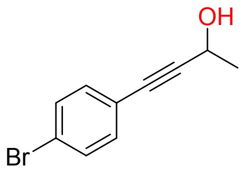

4-(4-bromophenyl)-3-butyn-2-ol was prepared following **General Procedure A** starting with 1-bromo-4-iodobenzene (2 mmol, 612 mg) and 3-butyne-2-ol (2.2 mmol, 154 mg). After purification by column chromatography the final compound was obtained as a yellow solid in 54 % yield (243 mg). ^{1}H NMR (400 MHz, $CDCl_3$) δ 7.48 – 7.45 (m, 2H), 7.32 – 7.29 (m, 2H), 4.81 – 4.74 (m, 1H), 2.21 (d, J = 5.2 Hz, 1H), 1.58 (d, J = 6.6 Hz, 3H). ^{13}C NMR (100 MHz, $CDCl_3$) δ 133.2, 131.6, 122.2, 121.6, 92.1, 83.1, 58.9, 24.4.

4-(3,5-Dimethylphenyl)but-3-yn-2-ol (9a)

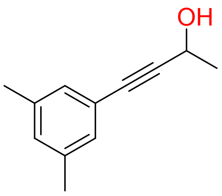

4-(3,5-Dimethylphenyl)but-3-yn-2-ol was prepared following **General Procedure A** starting with 1-iodo-3,5-dimethylbenzene (2 mmol, 512 mg) and 3-butyne-2-ol (2.2 mmol, 154 mg). After purification by column chromatography the final compound was obtained as an orange oil in 63 % yield (219 mg). ^{1}H NMR (400 MHz, $CDCl_3$) δ 7.10 – 7.09 (m, 2H), 6.99 – 6.98 (m, 1H), 4.81 – 4.74 (m, 1H), 2.31 (s, 6H), 2.00 (d, J = 5.3 Hz, 1H), 1.54 (d, J = 6.6 Hz, 3H). ^{13}C NMR (100 MHz, $CDCl_3$) δ 137.9, 130.3, 129.4, 122.3, 90.4, 84.4, 58.9, 24.5, 21.1.

4-[3-(trifluoromethyl)phenyl]but-3-yn-2-ol (10a)

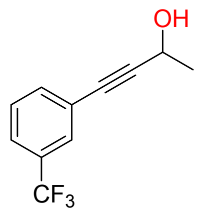

4-[3-(trifluoromethyl)phenyl]but-3-yn-2-ol was prepared following **General Procedure A** starting with 1-iodo-3-(trifluoromethyl)benzene (2 mmol, 592 mg)and 3-butyne-2-ol (2.2 mmol, 154 mg). After purification by column chromatography the final compound was obtained as an orange oil in 74 % yield (317 mg).^{1}H NMR (400 MHz, $CDCl_3$) δ 7.69 – 7.41 (m, 4H), 4.80 – 4.73 (m, 1H), 2.02 (d, J = 5.4 Hz, 1H), 1.56 (d, J = 6.6 Hz, 3H). ^{13}C NMR (100 MHz, $CDCl_3$) δ 134.8 (d, J = 1.2 Hz), 131.0 (d, J = 32.6 Hz), 128.9, 128.5 (q, J = 3.9 Hz), 125.0 (q, J = 3.9 Hz), 123.6, 122.4, 92.6, 82.6, 58.8, 24.3.

4-(3-hydroxybut-1-yn-1-yl)benzonitrile (11a)

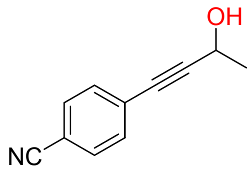

4-(3-hydroxybut-1-yn-1-yl)benzonitrile was prepared following **General Procedure A** starting with 4-iodobenzonitrile (2 mmol, 506 mg) and 3-butyne-2-ol (2.2 mmol, 154 mg). After purification by column chromatography the final compound was obtained as an orange oil in 43 % yield (147 mg). ^{1}H NMR (400 MHz, $CDCl_3$) δ 7.63 – 7.61 (m, 2H), 7.53 – 7.51 (m, 2H), 4.84 – 4.78 (m, 1H), 2.44 – 2.37 (m, 1H), 1.59 (d, J = 6.7 Hz, 3H).^{13}C NMR (100 MHz, $CDCl_3$) δ 132.3, 132.2, 127.7, 118.5, 111.9, 95.5, 82.6, 58.9, 24.3.

4-(Thiophen-2-yl)but-3-yn-2-ol (12a)

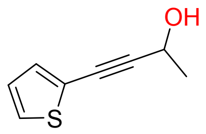

4-(Thiophen-2-yl)but-3-yn-2-ol was prepared following **General Procedure A** starting with 2-bromothiophene (2 mmol, 420 mg) and 3-butyne-2-ol (2.2 mmol, 154 mg). The reaction was heated to 45 °C for 20 hours. After purification by column chromatography the final compound was obtained as a yellow oil in 38 % yield (116 mg). ^{1}H NMR (400 MHz, $CDCl_3$) δ 7.30 – 7.28 (m, 1H), 7.24 – 7.23 (m, 1H), 7.01 – 6.99 (m, 1H), 4.84 – 4.77 (m, 1H), 2.15 (d, J = 5.3 Hz, 1H), 1.59 (d, J = 6.6 Hz, 3H). ^{13}C NMR (100 MHz, $CDCl_3$) δ 132.3, 127.3, 127.0, 122.6, 94.8, 77.4, 59.0, 24.3.

4-(3,5-Dimethoxyphenyl)but-3-yn-2-ol (13a)

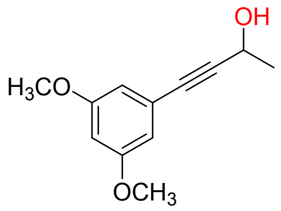

4-(3,5-Dimethoxyphenyl)but-3-yn-2-ol was prepared following **General Procedure A** starting with 1-iodo-3,5-dimethoxybenzene (2 mmol, 576 mg) and 3-butyne-2-ol (2.2 mmol, 154 mg). The reaction was heated to 70 °C. After 4.5 hours, additional $Pd(PPh_3)_2Cl_2$ (0.02 mmol, 14 mg), CuI (0.04 mmol, 7 mg), and 3-butyn-2-ol (0.05 mmol, 3.5 mg) were added under N_2. The temperature increased to 80 °C. The reaction was worked up after an additional heating for 2 hours at 80 ° C. After purification by column chromatography the final compound was obtained as an orange oil in 57 % yield (235 mg). ^{1}H NMR (400 MHz, $CDCl_3$) δ 6.58 – 6.57 (m, 2H), 6.44 – 6.43 (m, 1H), 4.77 – 4.71 (m, 1H), 3.77 (s, 6H), 2.05 (d, J = 5.1 Hz, 1H), 1.55 (d, J = 6.6 Hz, 3H). ^{13}C NMR (100 MHz, $CDCl_3$) δ 160.5, 123.9, 109.5, 101.9, 90.6, 84.0, 58.8, 55.4, 24.4.

Procedures for the synthesis of racemic propargyl amines

General procedures were adapted from literatures[9-11].

General procedures B: to a round bottom flask was added propargyl alcohol (1 mmol, 1.0 equiv), triphenylphosphine (1.1 mmol, 1.1 equiv) and phthalimide (1.1 mmol, 1.1 equiv). The flask was purged with N_2 and 10 mL THF was transferred via syringe under N_2 and at 0 °C. A solution of diethyl azodicarboxylate (DEAD) (40 % wt in toluene, 0.6 mL) was added at 0 °C. The reaction mixture was stirred for 20 h at room temperature or heated as necessary, and the reaction progress was monitored

by TLC. Upon completion, the crude mixture was filtered through a separation funnel, and the solid residue washed with ethyl acetate. The combined organic phase was concentrated under reduced pressure. Purification via flash column chromatography yielded the desired target compound.

General procedures C: to a round bottom flask was added the product from general procedure B (0.5 mmol, 1 equiv.). The flask was purged with N_2 and EtOH (26 mL), hydrazine hydrate (1 mL, 20 mmol 40 equiv.) was transferred via syringe under N_2. The reaction mixture was stirred at reflux for 4 h. The reaction progress was monitored by TLC. Upon completion, the combined organic phase was concentrated under reduced pressure. Purification via flash column chromatography yielded the desired target compound.

2-(4-(4-fluorophenyl)but-3-yn-2-yl)isoindoline-1,3-dione

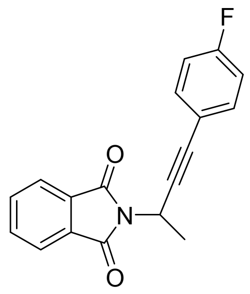

2-(4-(4-fluorophenyl)but-3-yn-2-yl)isoindoline-1,3-dione was prepared following **General Procedure B** starting with 4-(4-fluorophenyl)-3-butyn-2-ol (1 mmol, 164 mg), triphenylphosphine (1.1 mmol, 288 mg) and a phthalimide (1.1 mmol, 162 mg) . After purification by column chromatography (hexane : EtOAc = 10 : 1) the final compound was obtained as a white solid in 72 % yield (211 mg). ^{1}H NMR (400 MHz, $CDCl_3$) δ 7.92 – 7.87 (m, 2H), 7.78 – 7.74 (m, 2H), 7.46 – 7.43 (m, 2H), 7.02 – 6.98 (m, 2H), 5.45 (q, *J* = 7.1 Hz, 1H), 1.82 (d, *J* = 7.1 Hz, 3H).

2-(4-Phenylbut-3-yn-2-yl)isoindoline-1,3-dione

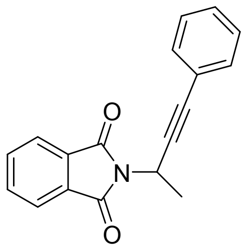

2-(4-Phenylbut-3-yn-2-yl)isoindoline-1,3-dione was prepared following **General Procedure B** starting with 4-Phenylbut-3-yn-2-ol (1 mmol, 146 mg), triphenylphosphine (1.1 mmol, 288 mg) and a phthalimide (1.1 mmol, 162 mg). After purification by column chromatography (hexane : EtOAc = 10 : 1) the final compound was obtained as an orange oil in 88 % yield (242 mg). ^{1}H NMR (400 MHz, $CDCl_3$) δ 7.93 – 7.90 (m, 2H), 7.80 – 7.76 (m, 2H), 7.50 – 7.48 (m, 2H), 7.34 – 7.32 (m, 3H), 5.49 (q, J = 7.1 Hz, 1H), 1.85 (d, J = 7.2 Hz, 3H).

2-(4-(4-chlorophenyl)but-3-yn-2-yl)isoindoline-1,3-dione

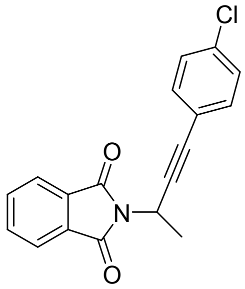

2-(4-(4-chlorophenyl)but-3-yn-2-yl)isoindoline-1,3-dione was prepared following **General Procedure B** starting with 4-(4-chlorophenyl)-3-butyn-2-ol (1 mmol, 180 mg), triphenylphosphine (1.1 mmol, 288 mg) and a phthalimide (1.1 mmol, 162 mg). After purification by column chromatography (hexane : EtOAc = 10 : 1) the final compound was obtained as a white solid in 79 % yield (244 mg). ^{1}H NMR (400 MHz, $CDCl_3$) δ 7.90 – 7.87 (m, 2H), 7.78 – 7.73 (m, 2H), 7.40 – 7.38 (m, 2H), 7.30 – 7.27 (m, 2H), 5.45 (q, J = 7.1 Hz, 1H), 1.82 (d, J = 7.1 Hz, 3H).

2-(4-(4-bromophenyl)but-3-yn-2-yl)isoindoline-1,3-dione

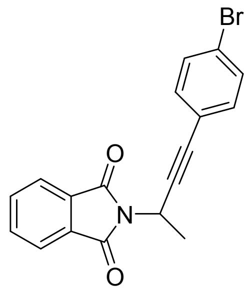

2-(4-(4-bromophenyl)but-3-yn-2-yl)isoindoline-1,3-dione was prepared following **General Procedure B** starting with 4-(4-bromophenyl)-3-butyn-2-ol (1 mmol, 224 mg), triphenylphosphine (1.1 mmol, 288 mg) and a phthalimide (1.1 mmol, 162 mg). After purification by column chromatography (hexane : EtOAc = 10 : 1) the final compound was obtained as a white solid in 77 % yield (273 mg).^{1}H NMR (400 MHz, $CDCl_3$) δ 7.91 – 7.88 (m, 2H), 7.78 – 7.74 (m, 2H), 7.45 – 7.43 (m, 2H), 7.33 – 7.30 (m, 2H), 5.45 (q, J = 7.1 Hz, 1H), 1.82 (d, J = 7.1 Hz, 3H).

2-(3-(1,3-dioxoisoindolin-2-yl)but-1-yn-1-yl)benzonitrile

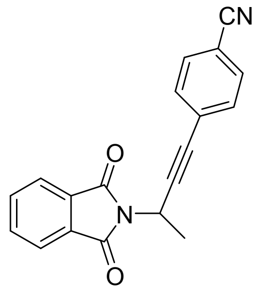

2-(3-(1,3-dioxoisoindolin-2-yl)but-1-yn-1-yl)benzonitrile was prepared following **General Procedure B** starting with 4-(3-hydroxybut-1-yn-1-yl)benzonitrile (1 mmol, 172 mg), triphenylphosphine (1.1 mmol, 288 mg) and a phthalimide (1.1 mmol, 162 mg) . After purification by column chromatography (hexane : EtOAc = 10 : 1) the final compound was obtained as a white solid in 58 % yield (174 mg). ^{1}H NMR (400 MHz, $CDCl_3$) δ 7.89 – 7.85 (m, 2H), 7.77 – 7.73 (m, 2H), 7.58 – 7.56 (m, 2H), 7.52 – 7.50 (m, 2H), 5.44 (q, J = 7.1 Hz, 1H), 1.79 (d, J = 7.1 Hz, 3H).

4-(4-fluorophenyl)but-3-yn-2-amine (1c)

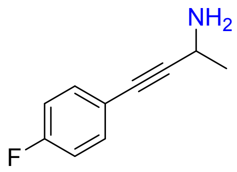

4-(4-fluorophenyl)but-3-yn-2-amine was prepared following **General Procedure C** starting with 2-(4-(4-fluorophenyl)but-3-yn-2-yl)isoindoline-1,3-dione (0.5 mmol, 147 mg) and hydrazine hydrate (40 mmol, 1 g). After purification by column chromatography (hexane : EtOAc = 1 : 2) the final compound was obtained as a yellow oil in 37 % yield (30 mg). ^{1}H NMR (400 MHz, $CDCl_3$) δ 7.43 – 7.39 (m, 2H), 7.04 – 7.00 (m, 2H), 3.95 (q, J = 6.8 Hz, 1H), 1.64 (s, 2H), 1.47 (d, J = 7.7 Hz, 3H).^{13}C NMR (100 MHz, $CDCl_3$) δ 162.3 (d, J = 248.8 Hz), 133.4 (d, J = 8.3 Hz), 119.3 (d, J = 3.6 Hz), 115.5 (d, J = 21.9 Hz), 93.5, 80.6, 39.4, 24.5.

4-phenylbut-3-yn-2-amine (6c)

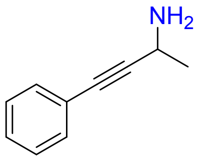

4-phenylbut-3-yn-2-amine was prepared following **General Procedure C** starting with 2-(4-Phenylbut-3-yn-2-yl)isoindoline-1,3-dione (0.5 mmol, 138 mg) and hydrazine hydrate (40 mmol, 1 g). After purification by column chromatography (hexane : EtOAc = 1 : 2) the final compound was obtained as a yellow oil in 64 % yield (46 mg). ^{1}H NMR (400 MHz, $CDCl_3$) δ 7.41 – 7.38 (m, 2H), 7.30 – 7.26 (m, 3H), 3.93 (q, J = 6.8 Hz, 1H), 1.78 (s, 2H), 1.44 (d, J = 6.7 Hz, 3H).^{13}C NMR (100 MHz, $CDCl_3$) δ 131.6, 128.3, 128.0, 123.3, 94.1, 81.5, 39.5, 24.6.

4-(4-chlorophenyl)but-3-yn-2-amine (7c)

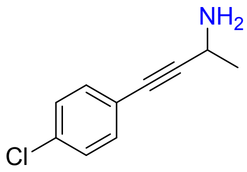

4-(4-chlorophenyl)but-3-yn-2-amine was prepared following **General Procedure C** starting with 2-(4-(4-chlorophenyl)but-3-yn-2-yl)isoindoline-1,3-dione (0.5 mmol, 155 mg) and hydrazine hydrate (40 mmol, 1 g). After purification by column chromatography (hexane:EtOAc = 1 : 2) the final compound was obtained as a yellow oil in 45 % yield (40 mg). ^{1}H NMR (400 MHz, $CDCl_3$) δ 7.34 – 7.26 (m, 4H), 3.92 (q, J = 6.8 Hz, 1H), 1.61 (s, 2H), 1.44 (d, J = 6.7 Hz, 3H).^{13}C NMR (100 MHz, $CDCl_3$) δ 134.0, 132.8, 128.6, 121.8, 94.8, 80.6, 39.4, 24.5.

4-(4-bromophenyl)but-3-yn-2-amine (8c)

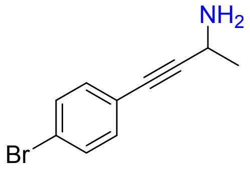

4-(4-bromophenyl)but-3-yn-2-amine was prepared following **General Procedure C** starting with 2-(4-(4-bromophenyl)but-3-yn-2-yl)isoindoline-1,3-dione (0.5 mmol, 177 mg) and hydrazine hydrate (40 mmol, 1 g). After purification by column chromatography (hexane : EtOAc = 1 : 2) the final compound was obtained as a yellow oil in 47 % yield (53 mg). ^{1}H NMR (400 MHz, $CDCl_3$) δ 7.48 – 7.45 (m, 2H), 7.31 – 7.28 (m, 2H), 3.95 (q, J = 6.8 Hz, 1H), 1.67 (s, 2H), 1.48 (d, J = 6.7 Hz, 3H). ^{13}C NMR (100 MHz, $CDCl_3$) δ 133.0, 131.5, 122.3, 122.2, 95.1, 80.6, 39.5, 24.5.

4-(3-aminobut-1-yn-1-yl)benzonitrile (11c)

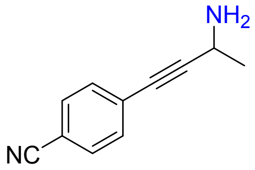

4-(3-aminobut-1-yn-1-yl)benzonitrile was prepared following **General Procedure C** starting with 2-(3-(1,3-dioxoisoindolin-2-yl)but-1-yn-1-yl)benzonitrile (0.5 mmol, 150 mg) and hydrazine hydrate (40 mmol, 1 g) . After purification by column chromatography (hexane : EtOAc = 1 : 2) yielded the final compound as a yellow oil in 32 % yield (27 mg). ^{1}H NMR (400 MHz, $CDCl_3$) δ 7.59 – 7.46 (m, 4H), 3.95 (q, J = 6.8 Hz, 1H), 1.61 (s, 2H), 1.45 (d, J = 6.7 Hz, 3H). ^{13}C NMR (100 MHz, $CDCl_3$) δ 132.1, 132.0, 128.3, 118.5, 111.4, 80.3, 63.6, 39.5, 24.3.

3-Phenylprop-2-yn-1-amine (15c)

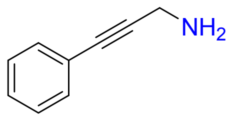

$Pd(PPh_3)_2Cl_2$ (0.02 mmol, 14 mg) and CuI (0.04 mmol, 7 mg) were suspended in THF/Et_3N (4：1) under N_2. Iodobenzene (5 mmol, 1.02 g) was added, followed by the addition of prop-2-yn-1-amine (5 mmol, 345 mg). The reaction was stirred at room temperature for 10 hours. Subsequent workup and column chromatography (hexane: EtOAc = 3 : 1) yielded the final compound as a yellow oil in 42 % yield (275mg). ^1H NMR (400 MHz, $CDCl_3$) δ 7.47 – 7.43 (m, 2H), 7.35 – 7.32 (m, 3H), 3.69 (s, 2H), 1.57 (s, 2H).

GC analysis

The products were analyzed by Gas Chromatography with a flame ionization detector (GC-FID, model: SHIMADZU GC-2010 Pro) equipped with the column SH-Rtx-1 Cap:(30 m × 0.25 mm × 0.25 µm), N_2 as the carrier gas. The temperature profile was: 100 °C holding for 1.3 min; 15 °C min^{-1} to 130 °C holding for 1.5 min; 30 °C min^{-1} to 310 °C for 1.2 min.

The enantiomeric excess of propargylic amines was determined by chiral GC. The column was LIPODEX E (25m×0.25mm×0.25um). In order to separate the two enantiomers, 3 mg of *N,N*-dimethylpyridin-4-amine (DMAP) and 10 uL of acetic anhydride were added to the ethyl acetate after extracting the reaction mixture. The derivatization was performed at 30 °C for 45 minutes, then 100 uL of MilliQ water was added to stop the acetylation. The organic phase was dried over $MgSO_2$ and measured by chiral GC.

GC-MS analysis

Sample analysis: Electron ionization (EI) GC-MS data were collected on an Agilent model 7890A GC with a DB-5 fused silica capillary column (30 m length, 0.25 mm inner diameter, 0.25 µm film thickness), Agilent 7200 Q-TOF mass selective detector and 7683B autosampler. The GC was programmed from 100 °C (held for 1.3 min) to 130 °C at 15 °C min^{-1} (held for 1.5 min); 30 °C min^{-1} to 300 °C holding for 1.2 min; the injection port temperature was 250 °C, and the transfer line temperature was 280 °C using the following parameters: ultra-high purity helium carrier gas, column flow at 1 mL min^{-1}, injection port

temperature 250 °C, transfer line temperature 280 °C. The MS were scanned through full-scan data acquisition from 35 to 550 atomic mass units.

HPLC analysis

Unless otherwise specified, the alcohol products were analyzed by High Performance Liquid Chromatography (HPLC) with a UV detector (model: SHIMADZU CORPORATION LC-2010C HT), using a CHIRALCEL OJ-H column; the mobile phase is n-Hexane : ^{i}PrOH = 90 : 10; flow rate = 1 mL/min; λ = 254 nm; T = 30 °C.

The exceptions were: for **2a**, the mobile phase was n-heptane : ^{i}PrOH (98 : 2).

NMR analysis

^{1}H NMR spectra were recorded at 298.2 K on a Bruker AVANCE III 400 MHz NMR spectrometer (Bruker Biospin, Germany). (7.47 μs 90 pulse, and a relaxation delay of 1.00 s, 16 scans).

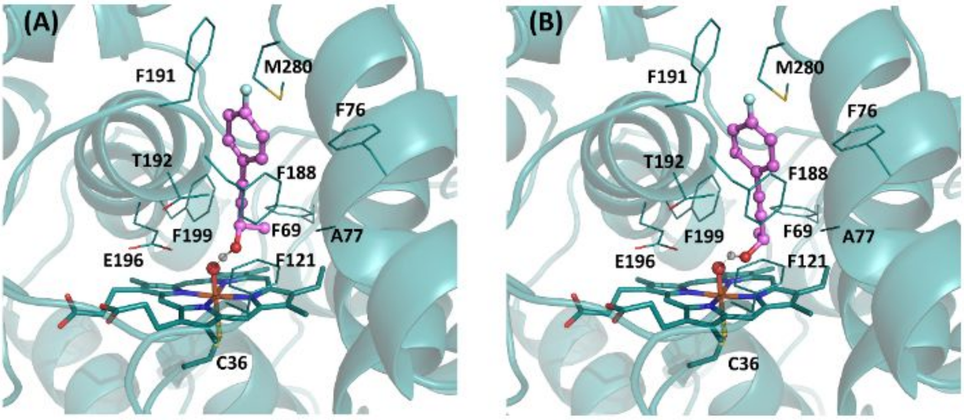

Figure S1. Active site model of PaDa-I in complex with (*R*)-1a (A, -4.82 kcal/mol) and (*S*)-1a (B, -4.72 kcal/mol), respectively.

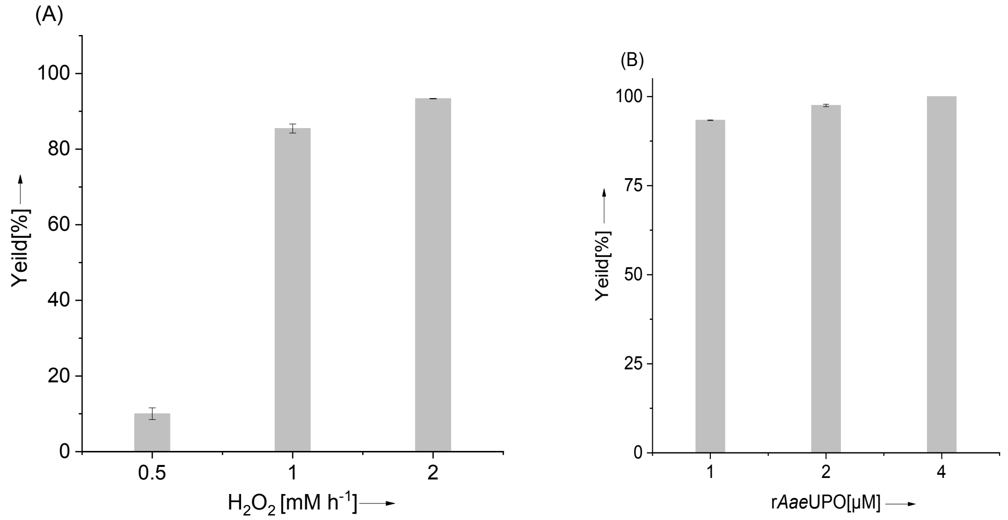

Figure S2. r*Aae*UPO catalysed propargylic alcohol oxidation using 4-(4-fluorophenyl)-3-butyn-2-ol as substrate. The influence of H_2O_2 dose rate (A) and enzyme concentration (B) on the reaction yield. Conditions: [r*Aae*UPO] = 1- 4 µM, [substrate] = 5 mM, NaPi buffer (100 mM, pH 8), 30 % (v/v) MeCN as cosolvent, [H_2O_2] = 0.5-2 mM h^{-1}, [total H_2O_2] = 10 mM, 30 °C, 800 rpm, 5 h. The yield was calculated by gas chromatography: [ketone **1a**]$_{final}$ × ([alcohol **1**]$_{final}$ + [ketone **1a**]$_{final}$)$^{-1}$.

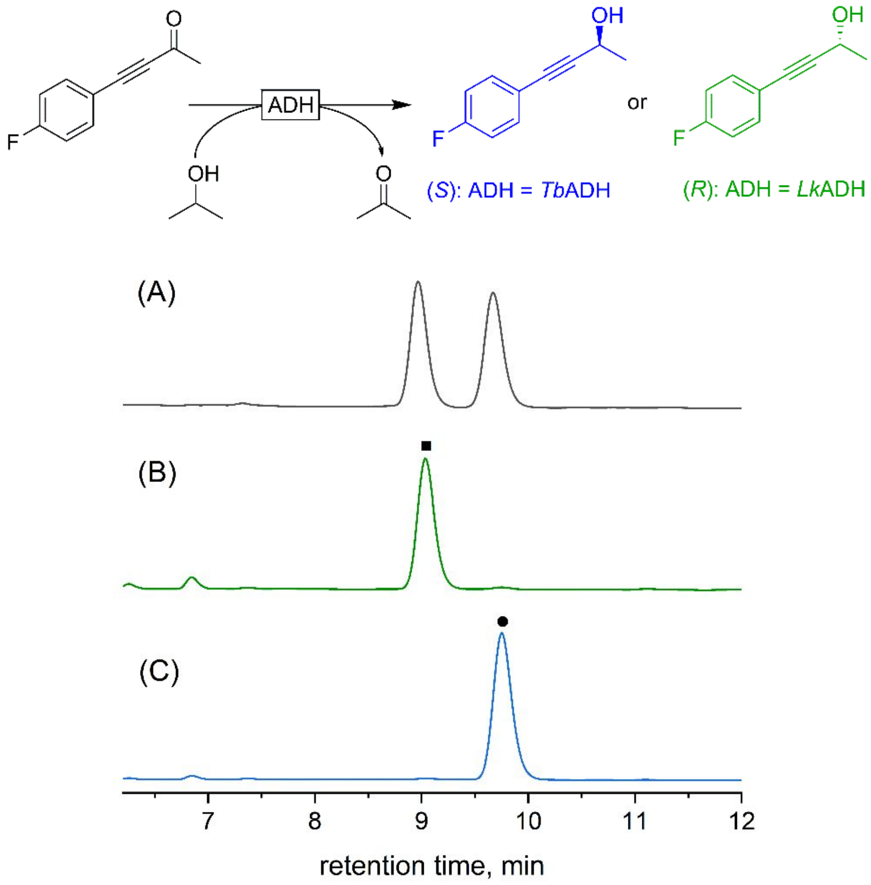

Figure S3. Representative chiral HPLC chromatograms of racemic racemic-**1a** (A), (*R*)-**1a**, B, ■, reduced by *Lk*ADH) and (*S*)-**1a**, C, ●, reduced by *Tb*ADH), respectively. Conditions: [substrate **1a**] = 5 mM, [*Lk*ADH] = 60 µM or [*Tb*ADH] = 50 µM, NaPi buffer (100 mM, pH 8), 5% isopropanol, [lysozyme] = 1mg mL^{-1}, [Dnase I] = 6U mL^{-1}, 30 ^{o}C, 800 rpm.

4-(4-fluorophenyl)-3-butyn-2-ol

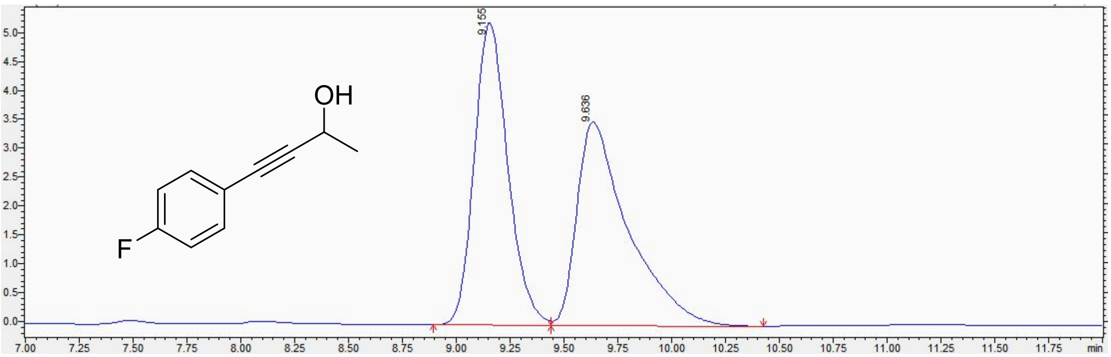

Peak	Retention time (min)	Peak area (mV*min)	Peak height (mV)	Peak area (%)
1	9.155	568626	52271	49.352
2	9.636	583561	87463	50.648

Figure S4. Representative HPLC chromatogram of racemic 1a .

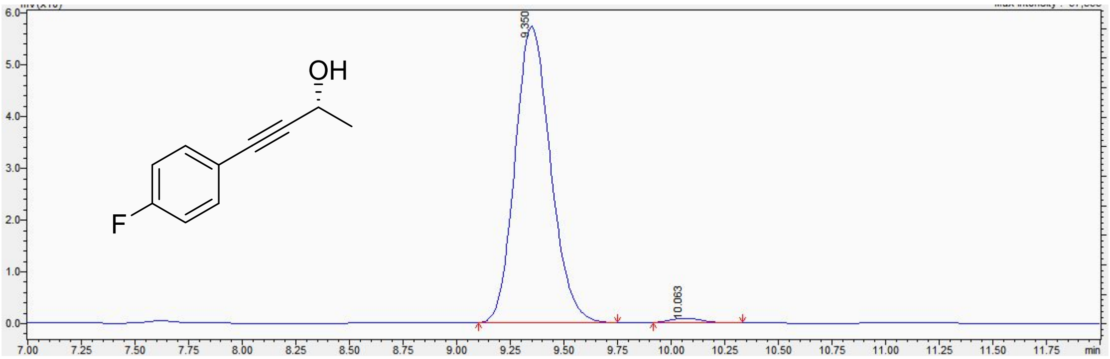

Peak	Retention time (min)	Peak area (mV*min)	Peak height (mV)	Peak area (%)
1	9.350	658798	57374	98.776
2	10.063	8166	831	1.224

Figure S5. Representative HPLC chromatogram of (*R*)-1a.

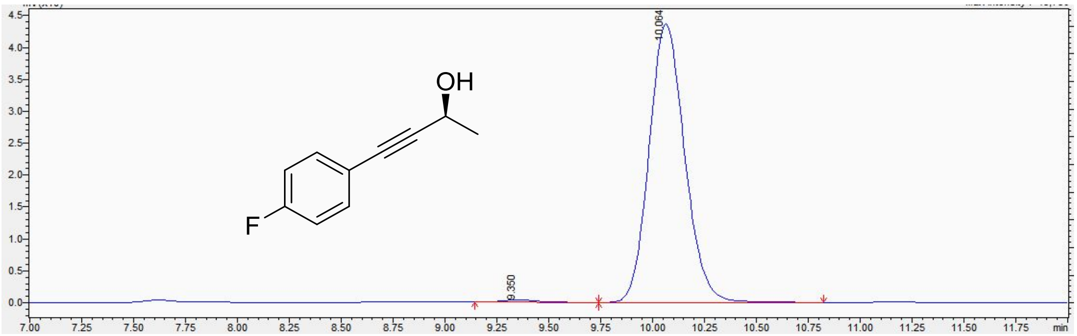

Peak	Retention time (min)	Peak area (mV*min)	Peak height (mV)	Peak area (%)
1	9.350	4350	373	0.829
2	10.064	520310	43709	99.171

Figure S6. Representative HPLC chromatogram of (*S*)-**1a**.

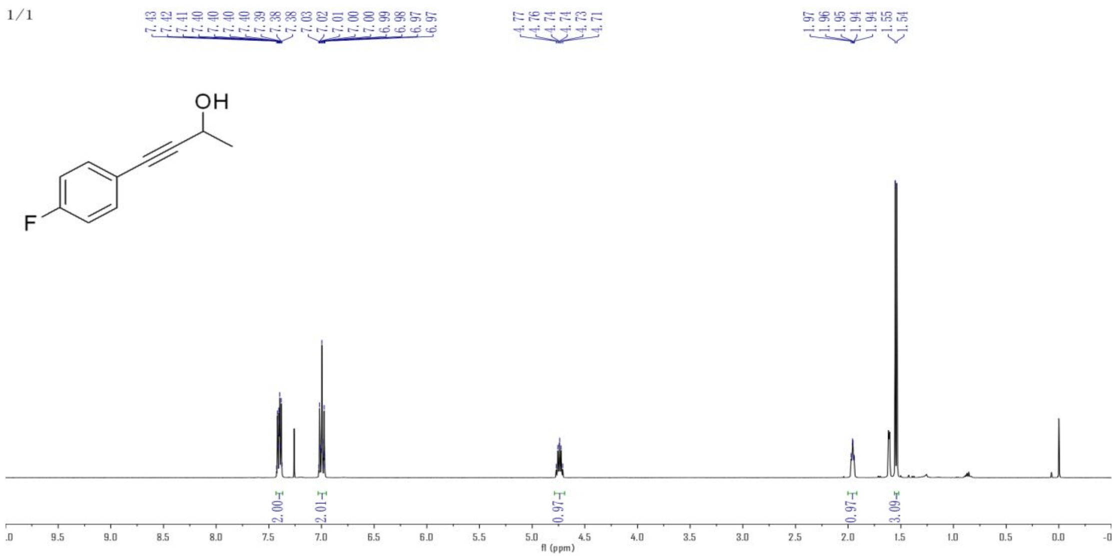

Figure S7. ^{1}H NMR (400 MHz) spectrum of racemic 4-(4-fluorophenyl)-3-butyn-2-ol (**1a**) in $CDCl_3$.

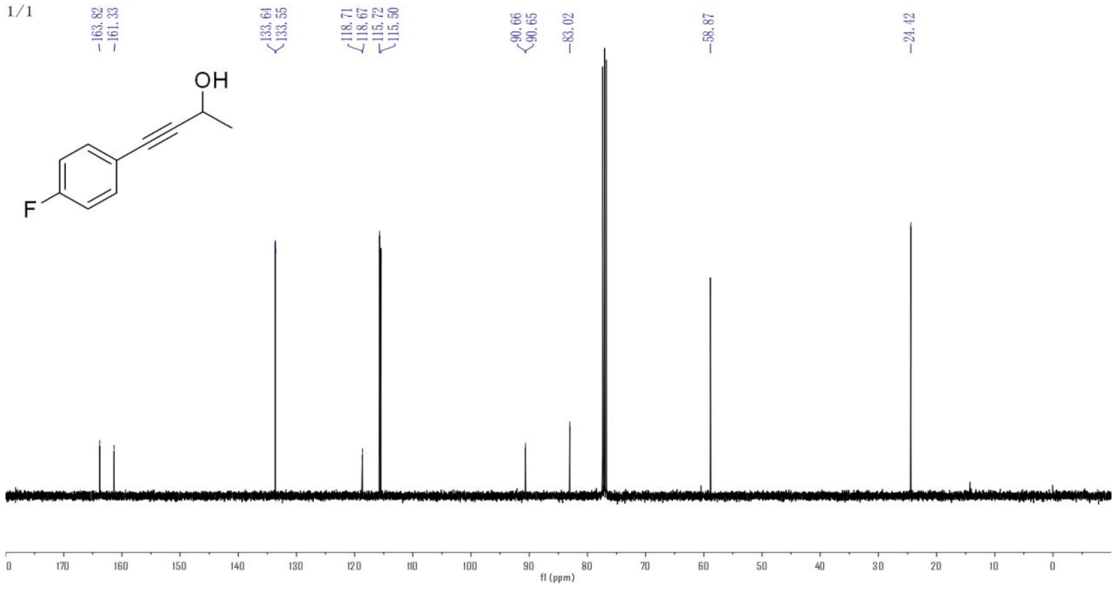

Figure S8. ^{13}C NMR (100 MHz) spectrum of racemic 4-(4-fluorophenyl)-3-butyn-2-ol (**1a**) in $CDCl_3$.

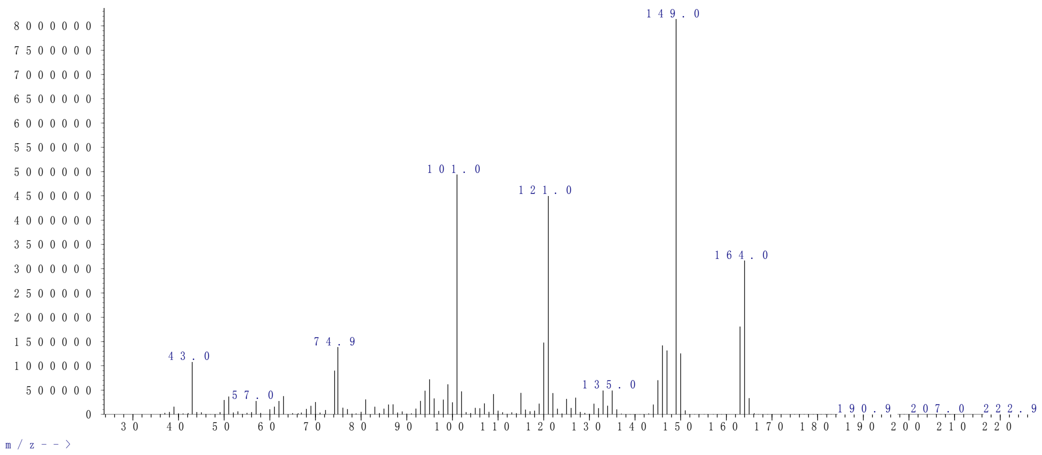

Figure S9. GC-MS spectrum of racemic 4-(4-fluorophenyl)-3-butyn-2-ol (**1a**).

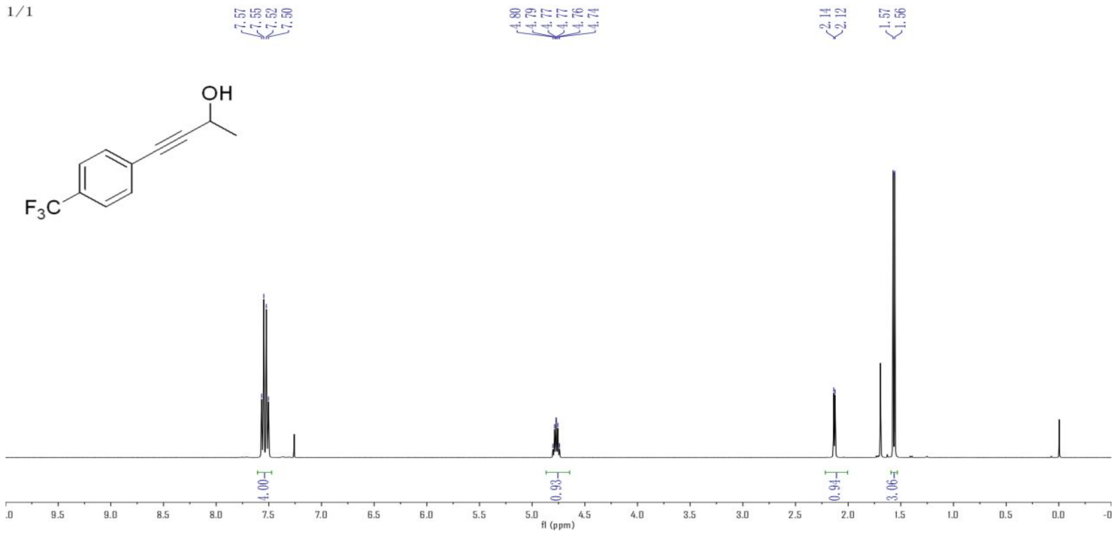

Figure S10. ^{1}H NMR (400 MHz) spectrum of racemic 4-(4-(Trifluoromethyl)phenyl)but-3-yn-2-ol (**2a**) in $CDCl_3$.

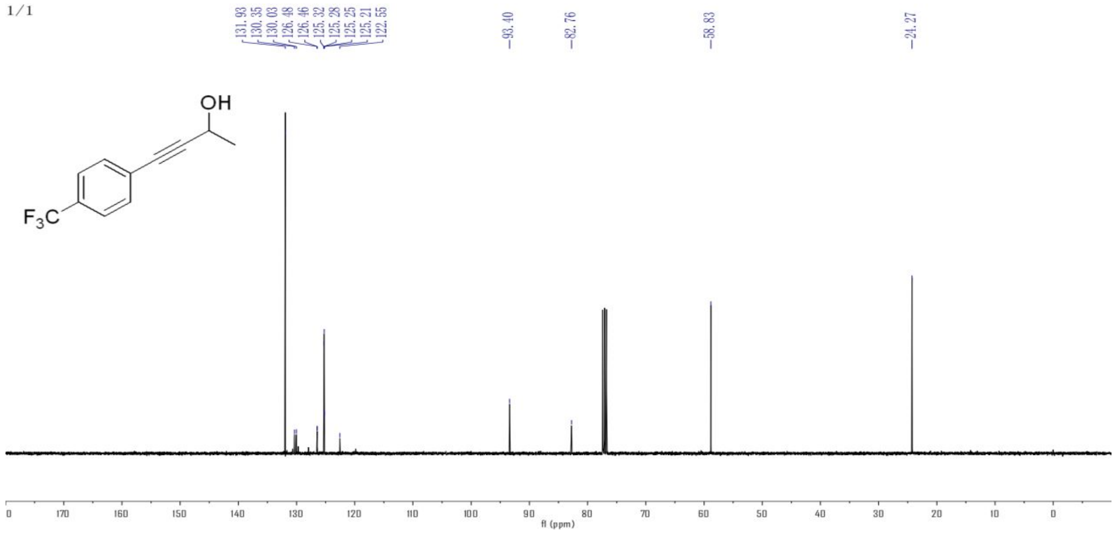

Figure S11. ^{13}C NMR (100 MHz) spectrum of racemic 4-(4-(Trifluoromethyl)phenyl)but-3-yn-2-ol (**2a**) in $CDCl_3$.

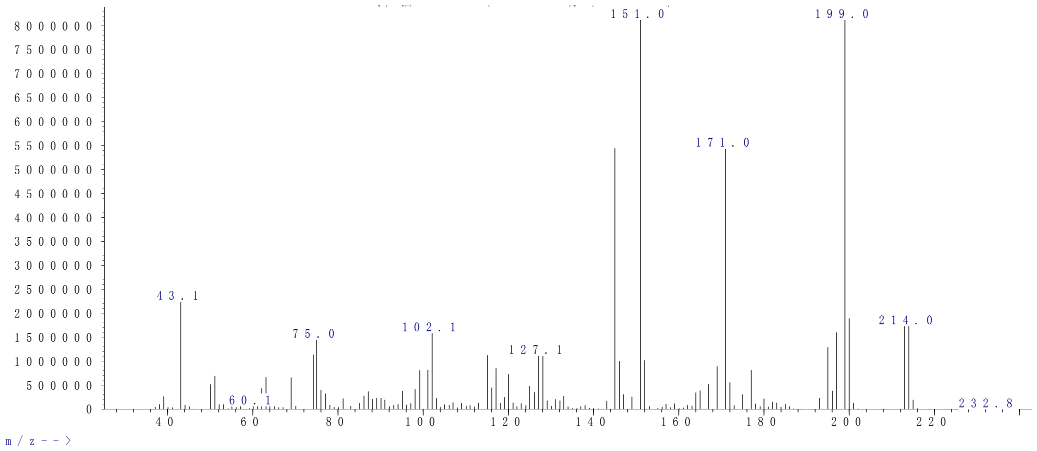

Figure S12. GC-MS spectrum of racemic 4-(4-(Trifluoromethyl)phenyl)but-3-yn-2-ol (**2a**).

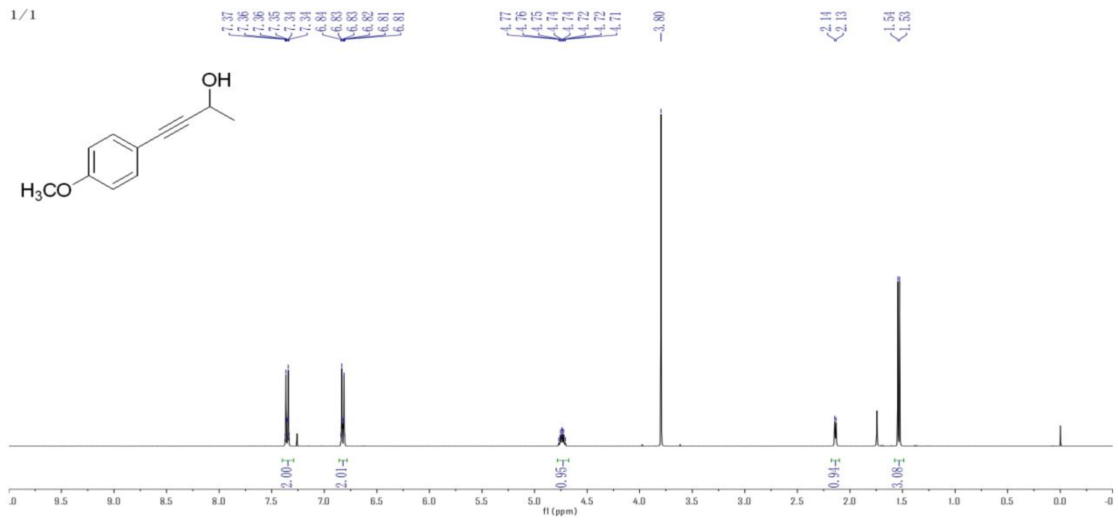

Figure S13. ^{1}H NMR (400 MHz) spectrum of racemic 4-(4-Methoxyphenyl)but-3-yn-2-ol (**3a**) in $CDCl_3$.

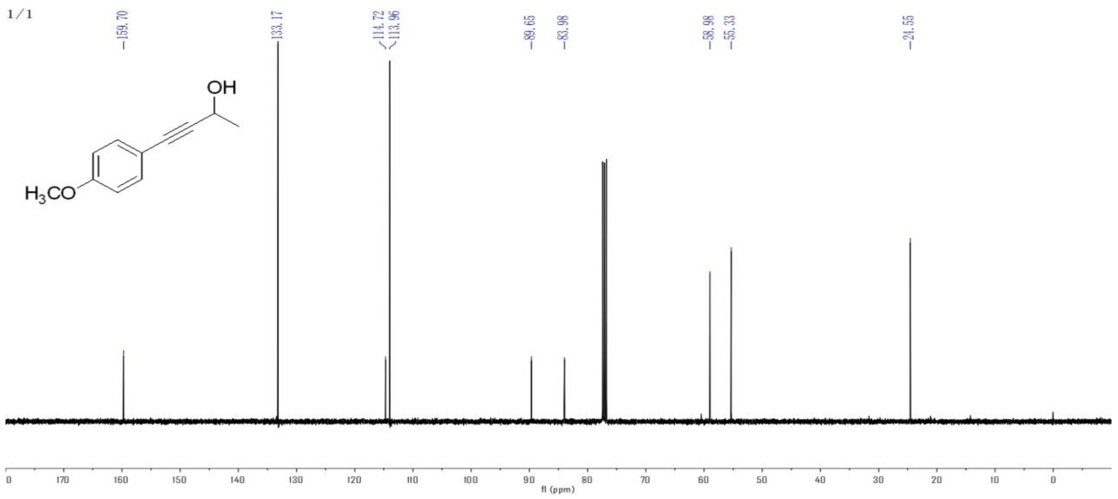

Figure **S14**. ^{13}C NMR (100 MHz) spectrum of racemic 4-(4-Methoxyphenyl)but-3-yn-2-ol (**3a**) in $CDCl_3$.

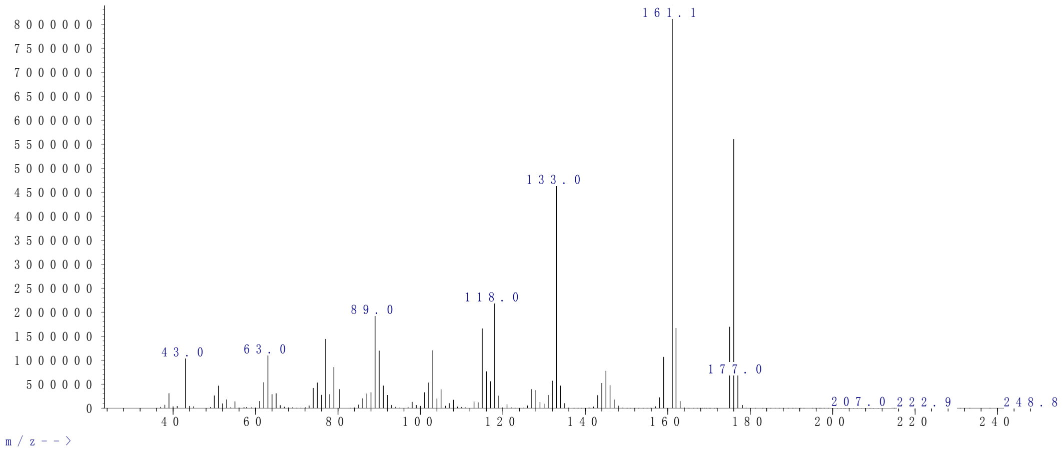

Figure S15. GC-MS spectrum of racemic 4-(4-Methoxyphenyl)but-3-yn-2-ol (**3a**).

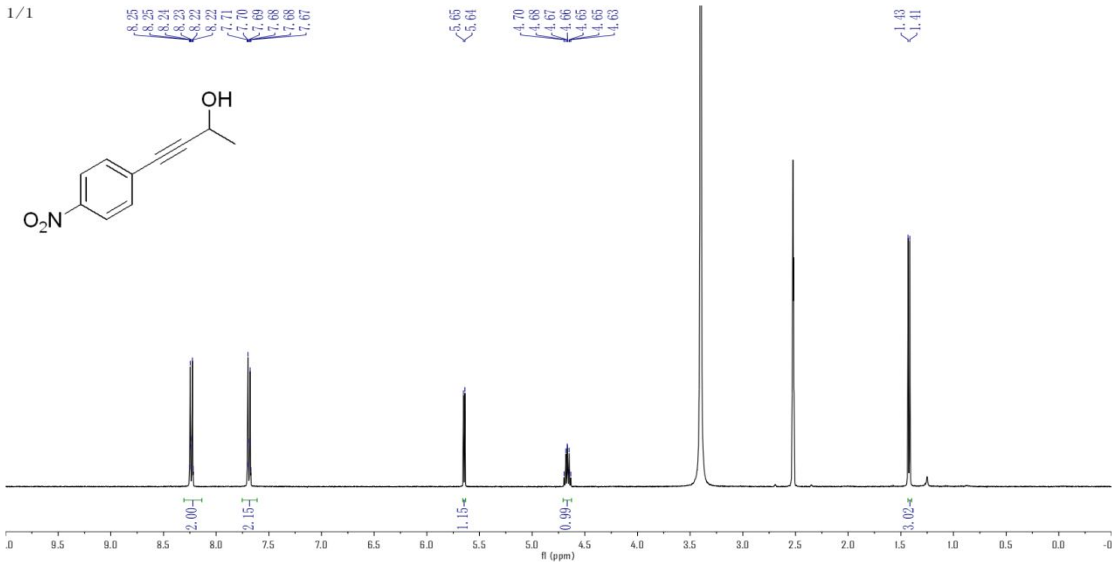

Figure S16. ^{1}H NMR (400 MHz) spectrum of racemic 4-(4'-nitrophenyl)-3-butyn-2-ol (**4a**) in DMSO.

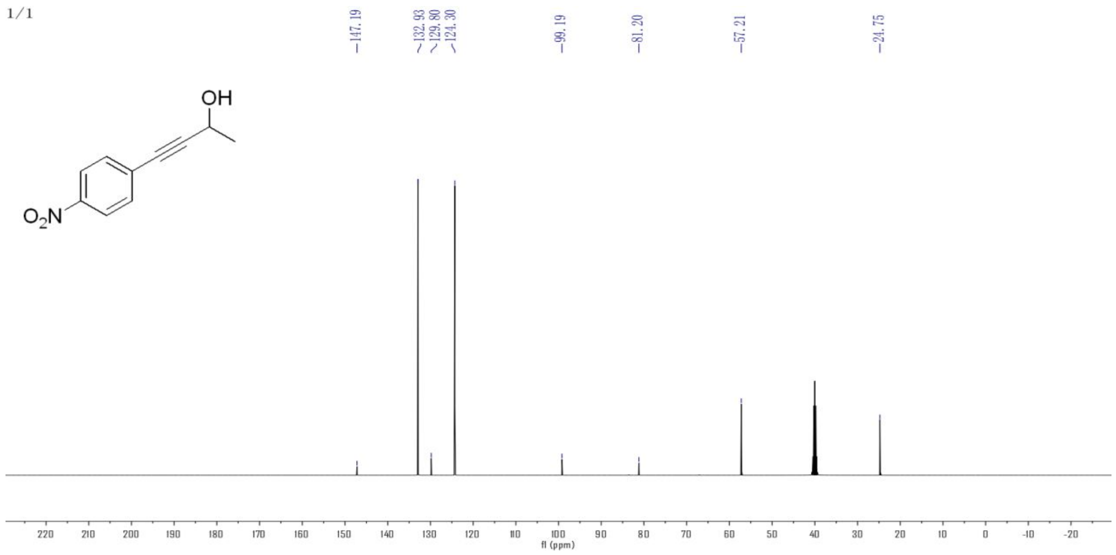

Figure S17. ^{13}C NMR (100 MHz) spectrum of racemic 4-(4'-nitrophenyl)-3-butyn-2-ol (**4a**) in DMSO.

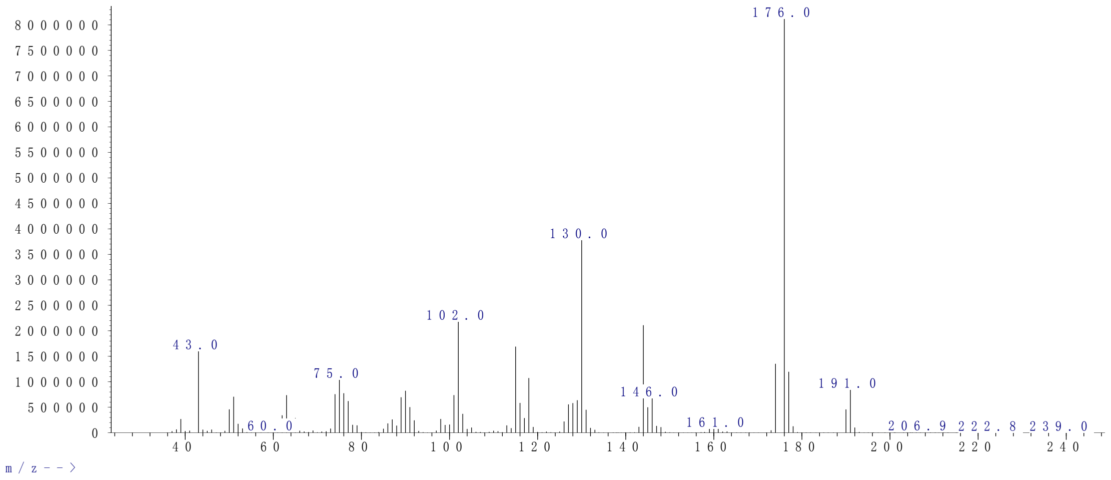

Figure S18. GC-MS spectrum of racemic 4-(4'-nitrophenyl)-3-butyn-2-ol (**4a**).

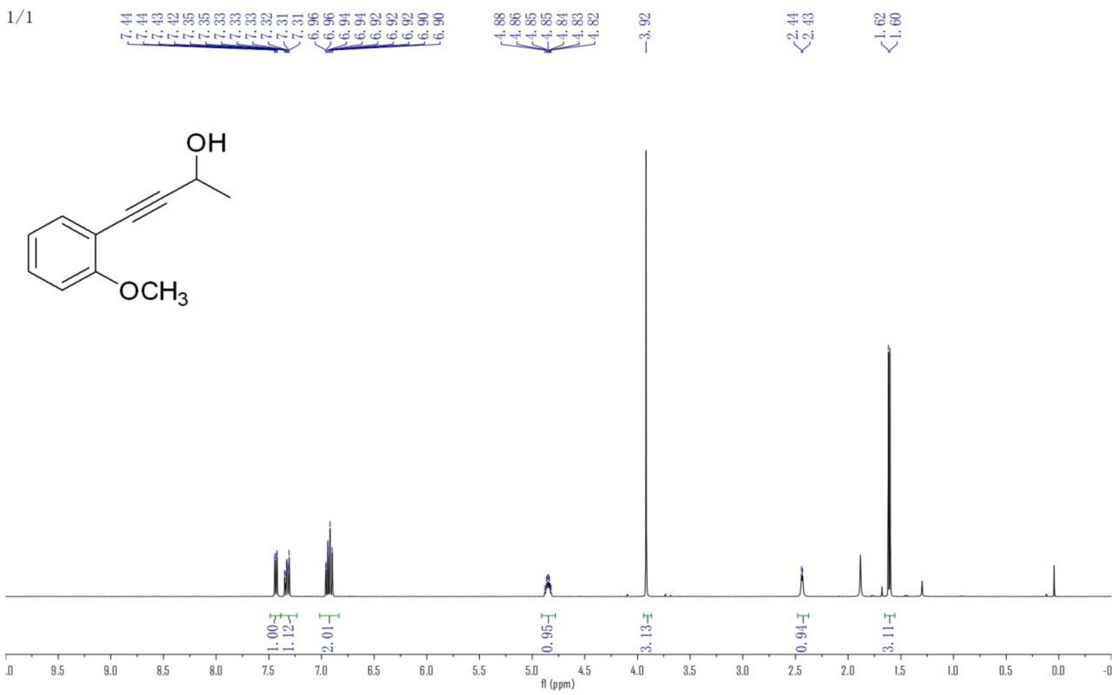

Figure S19. ^{1}H NMR (400 MHz) spectrum of racemic 4-(2-Methoxyphenyl)but-3-yn-2-ol (**5a**) in $CDCl_3$.

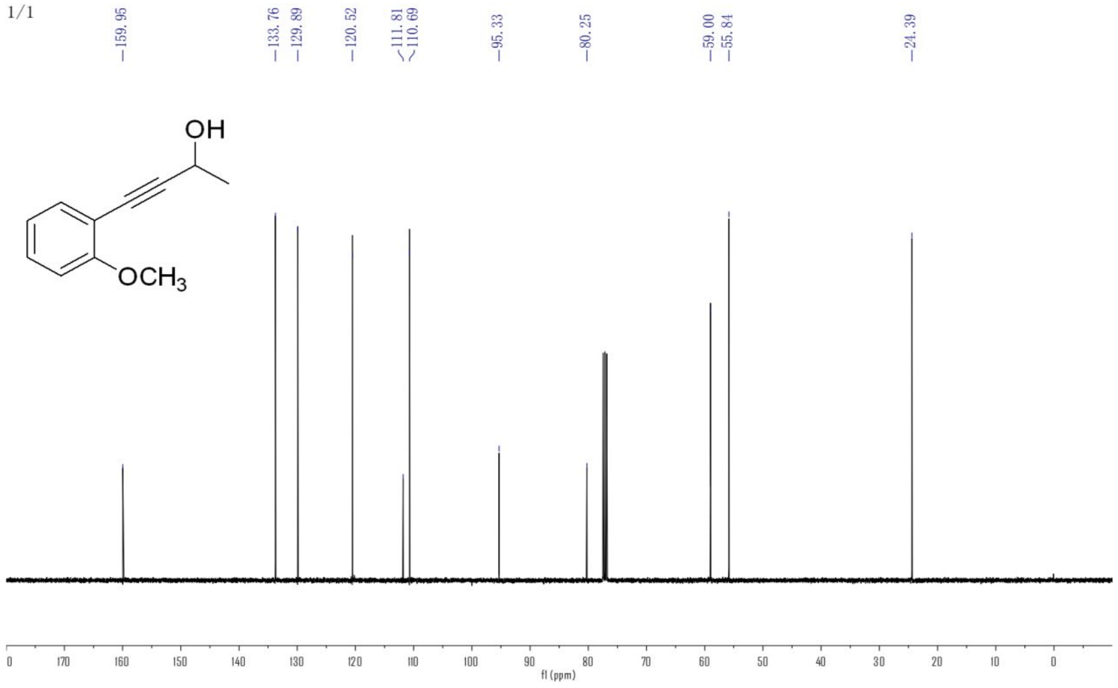

Figure S20. ^{13}C NMR (100 MHz) spectrum of racemic 4-(2-Methoxyphenyl)but-3-yn-2-ol (**5a**) in $CDCl_3$.

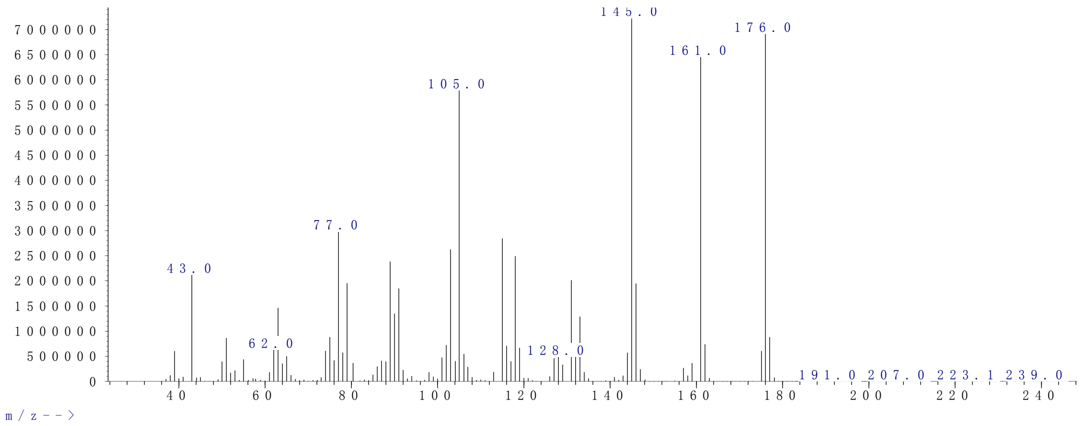

Figure S21. GC-MS spectrum of racemic 4-(2-Methoxyphenyl)but-3-yn-2-ol (**5a**).

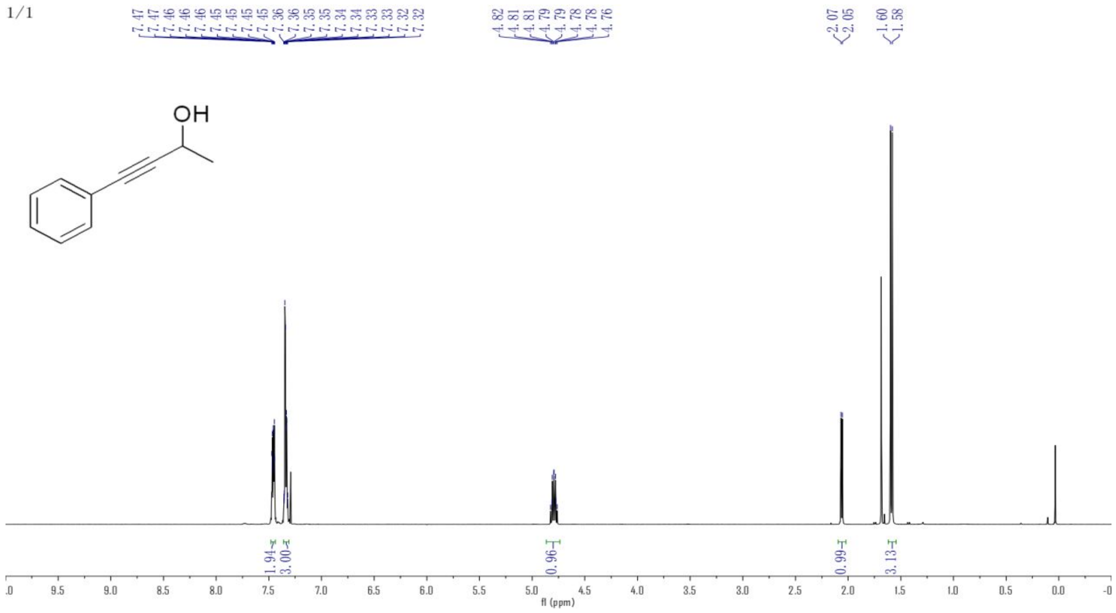

Figure S22. ^{1}H NMR (400 MHz) spectrum of racemic 4-Phenylbut-3-yn-2-ol (**6a**) in $CDCl_3$.

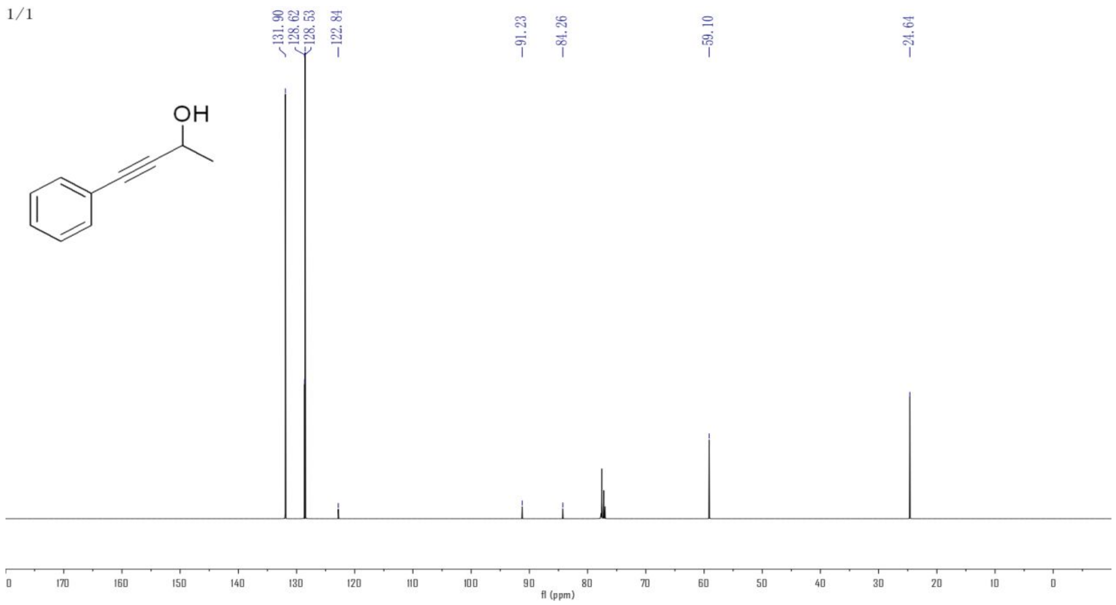

Figure S23. ^{13}C NMR (100 MHz) spectrum of racemic 4-Phenylbut-3-yn-2-ol (**6a**) in $CDCl_3$.

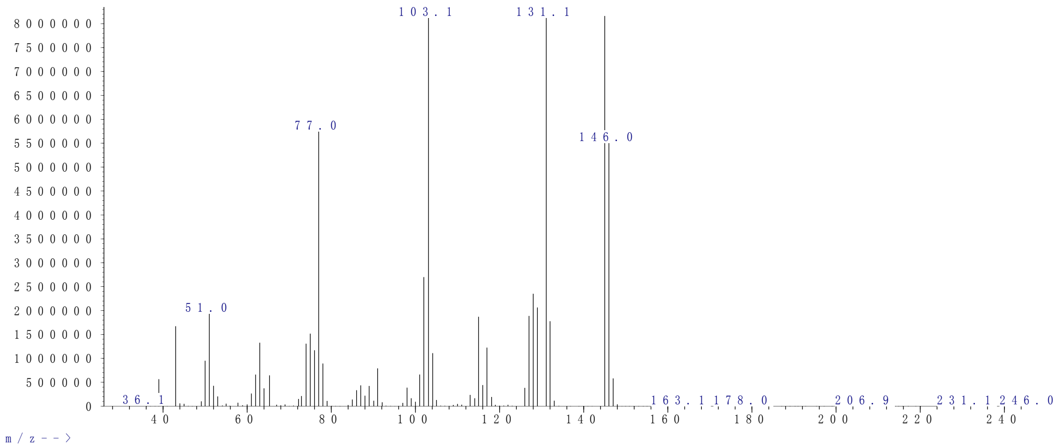

Figure S24. GC-MS spectrum of racemic 4-Phenylbut-3-yn-2-ol (**6a**).

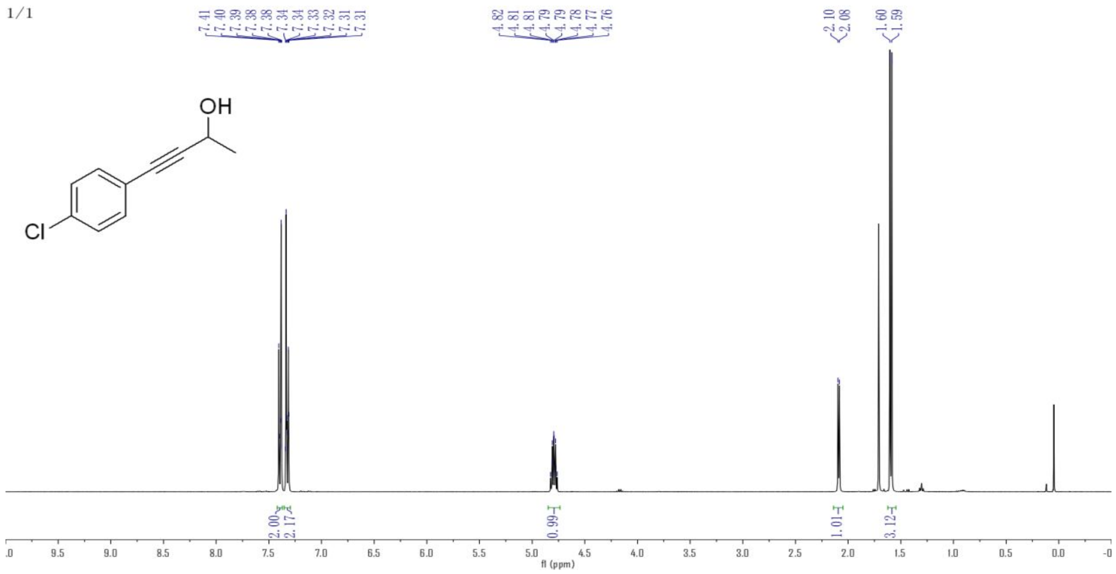

Figure S25. ^{1}H NMR (400 MHz) spectrum of racemic 4-(4-chlorophenyl)-3-butyn-2-ol (**7a**) in $CDCl_3$.

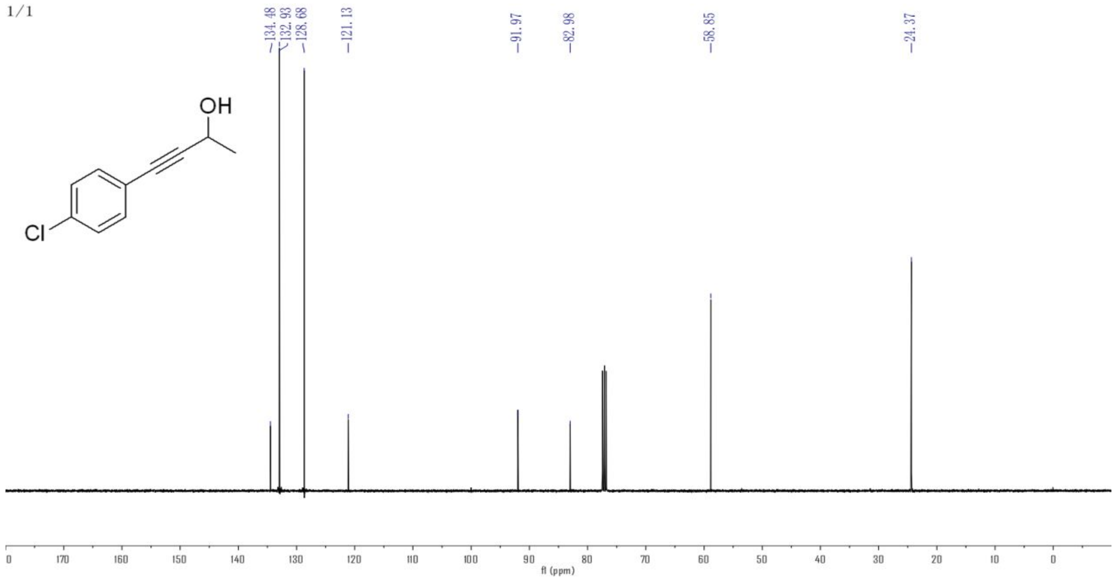

Figure S26. ^{13}C NMR (100 MHz) spectrum of racemic 4-(4-chlorophenyl)-3-butyn-2-ol (**7a**) in $CDCl_3$.

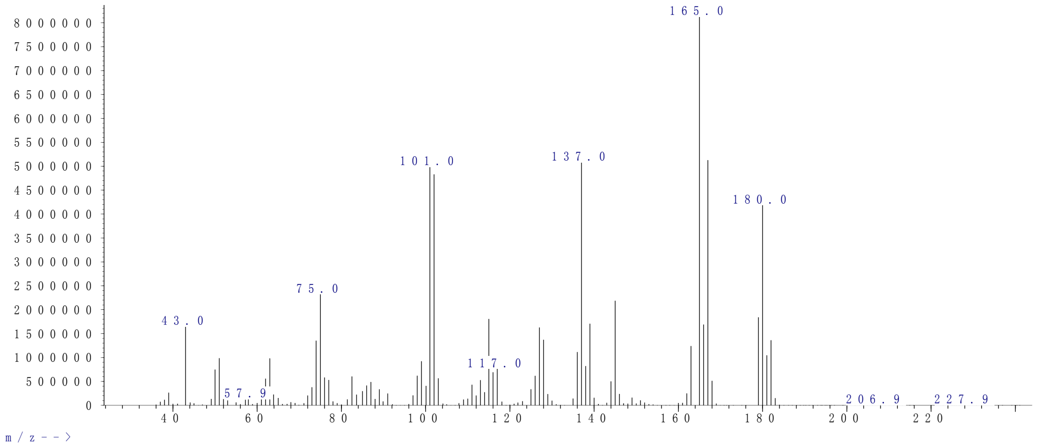

Figure S27. GC-MS spectrum of racemic 4-(4-chlorophenyl)-3-butyn-2-ol (**7a**).

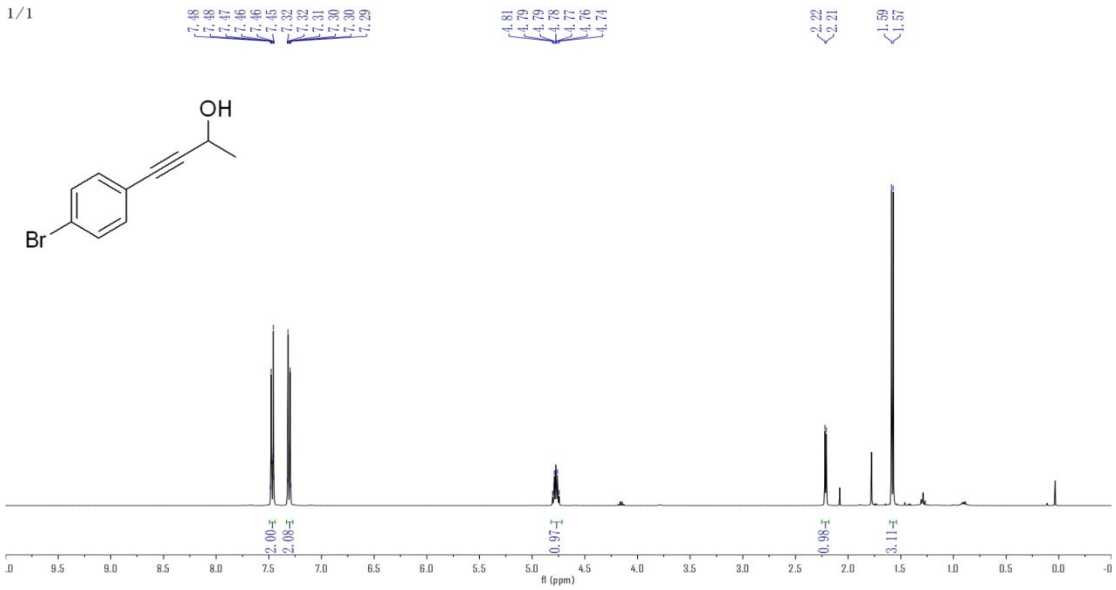

Figure S28. ^{1}H NMR (400 MHz) spectrum of racemic 4-(4-bromophenyl)-3-butyn-2-ol (**8a**) in $CDCl_3$.

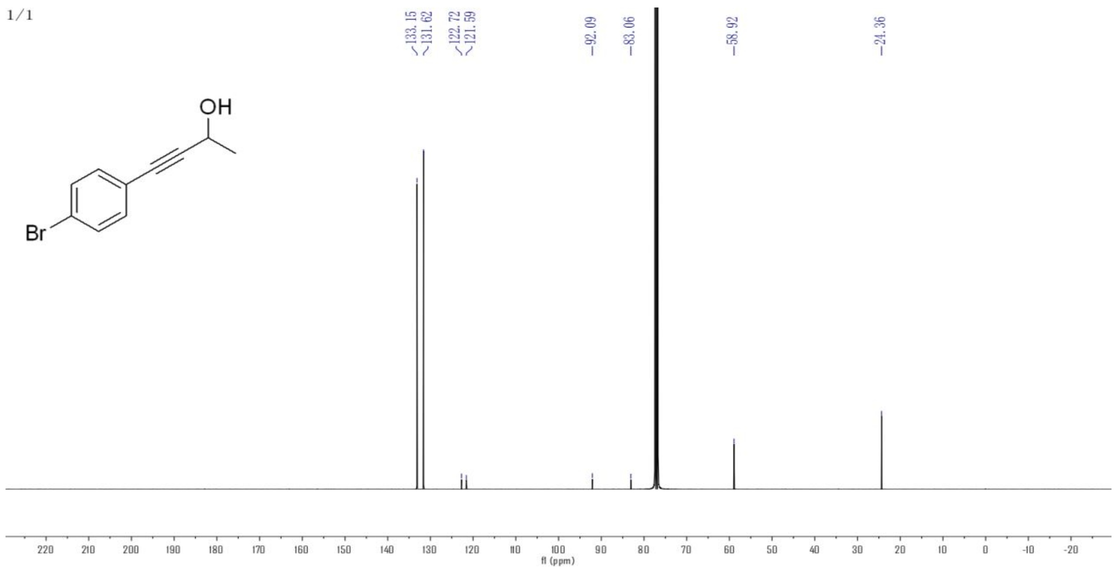

Figure S29. ^{13}C NMR (100 MHz) spectrum of racemic 4-(4-bromophenyl)-3-butyn-2-ol (**8a**) in $CDCl_3$.

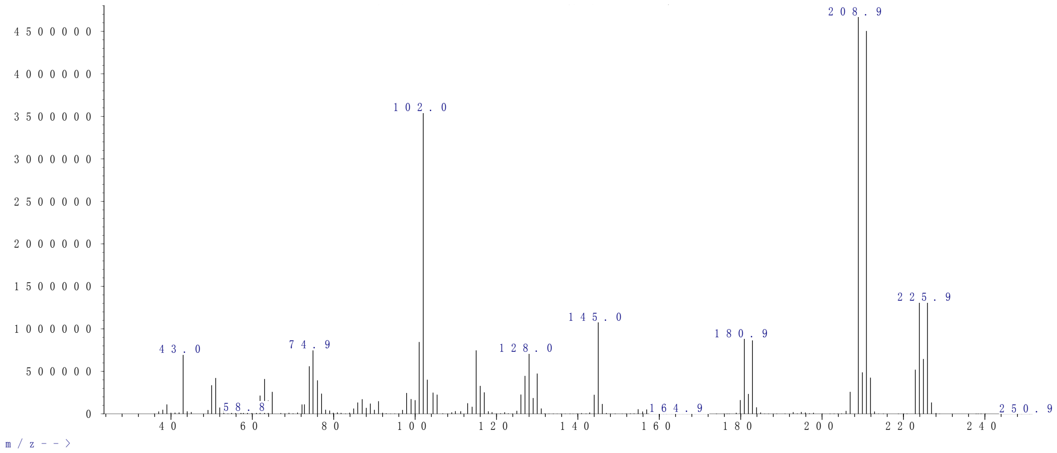

Figure S30. GC-MS spectrum of racemic 4-(4-bromophenyl)-3-butyn-2-ol (**8a**).

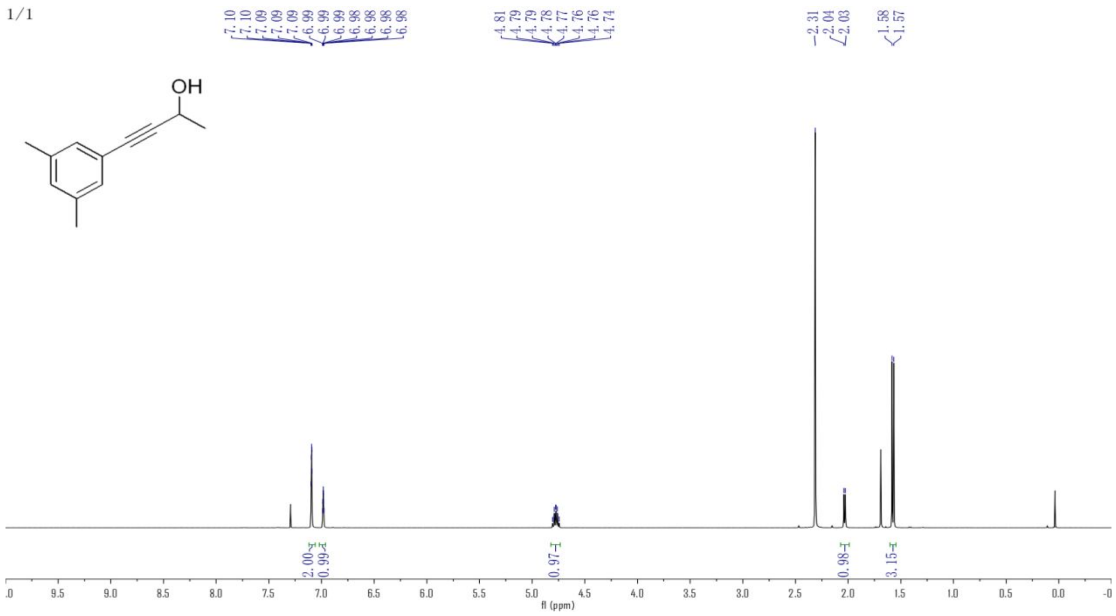

Figure S31. ^1H NMR (400 MHz) spectrum of racemic 4-(3,5-Dimethylphenyl)but-3-yn-2-ol (**9a**) in $CDCl_3$.

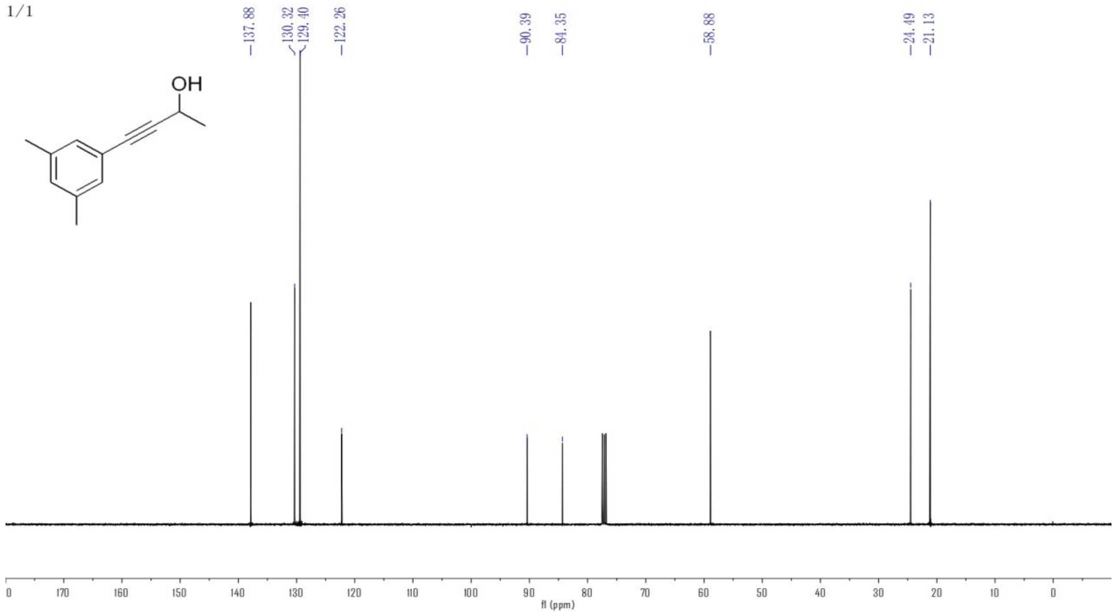

Figure S32. ^{13}C NMR (100 MHz) spectrum of racemic 4-(3,5-Dimethylphenyl)but-3-yn-2-ol (**9a**) in $CDCl_3$.

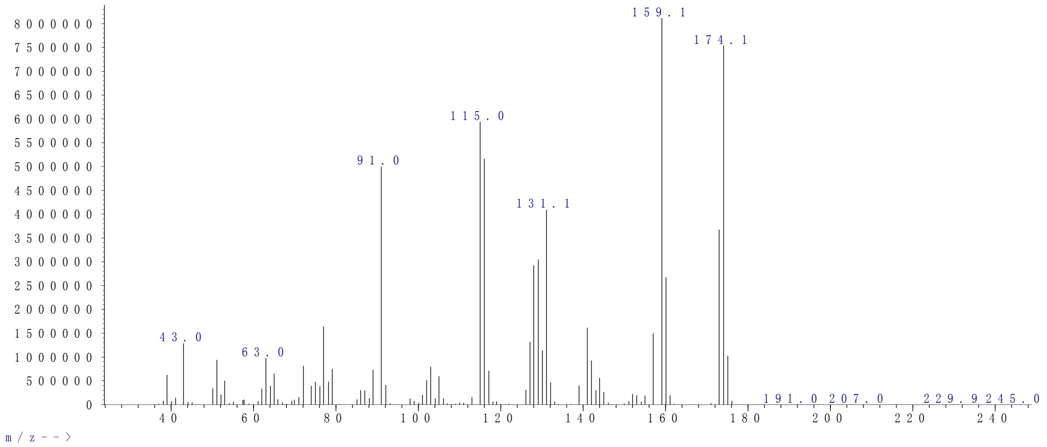

Figure S33. GC-MS spectrum of racemic 4-(3,5-Dimethylphenyl)but-3-yn-2-ol (**9a**).

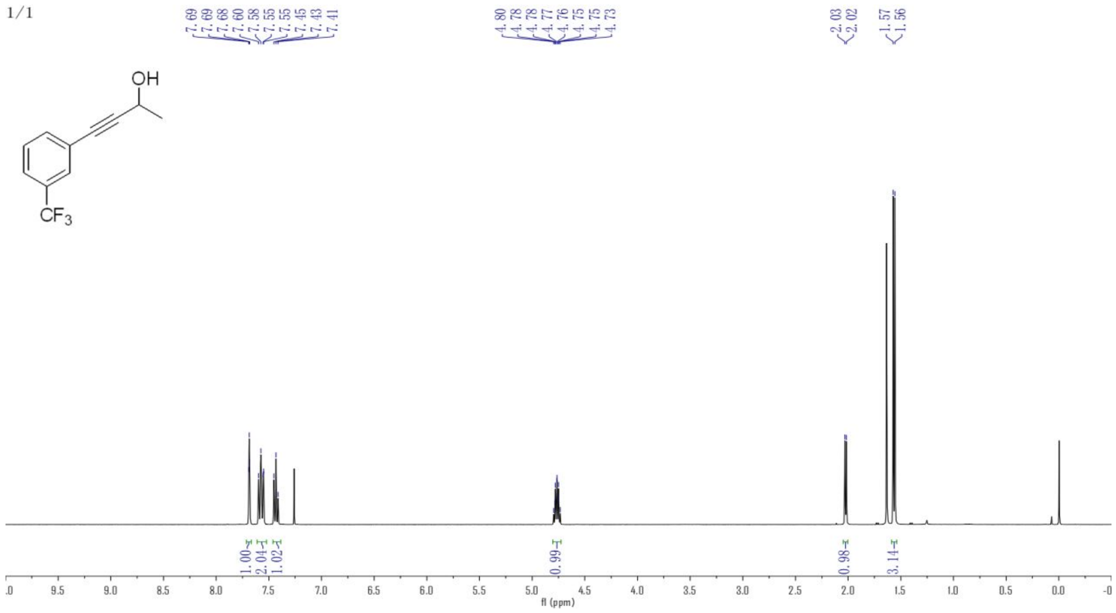

Figure S34. ^{1}H NMR (400 MHz) spectrum of racemic 4-[3-(trifluoromethyl)phenyl]but-3-yn-2-ol (**10a**) in $CDCl_3$.

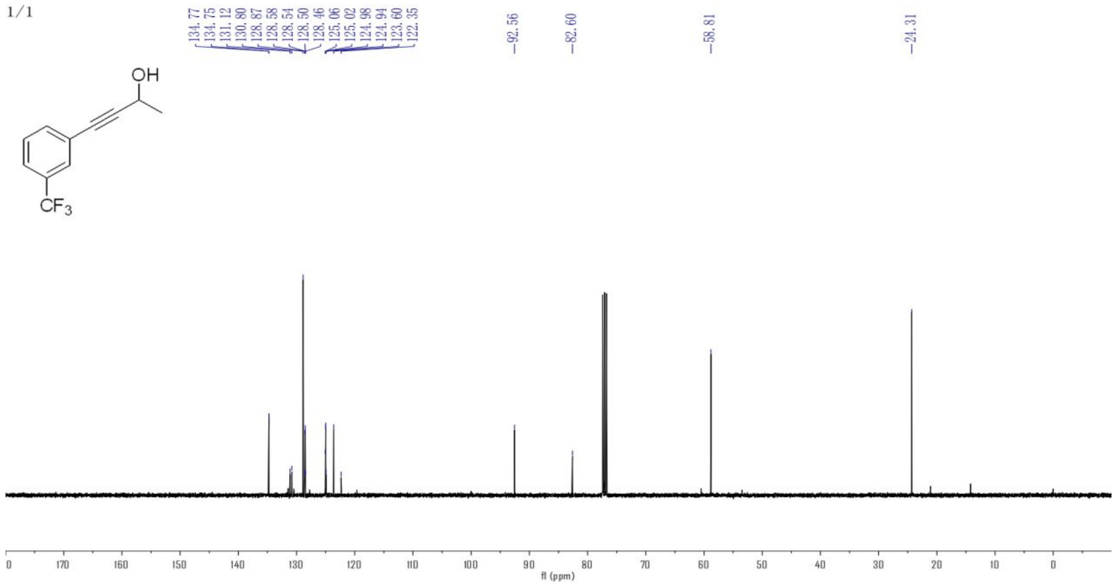

Figure S35. ^{13}C NMR (100 MHz) spectrum of racemic 4-[3-(trifluoromethyl)phenyl]but-3-yn-2-ol (**10a**) in $CDCl_3$.

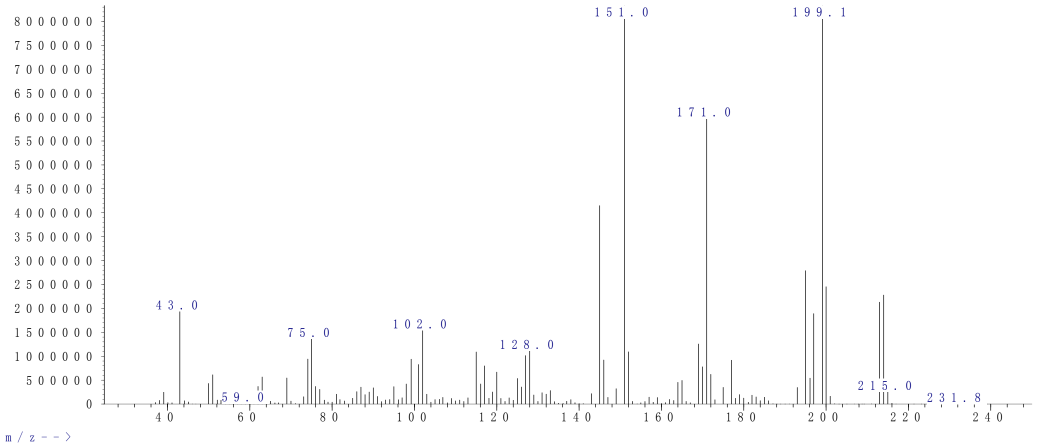

Figure S36. GC-MS spectrum of racemic 4-[3-(trifluoromethyl)phenyl]but-3-yn-2-ol (**10a**).

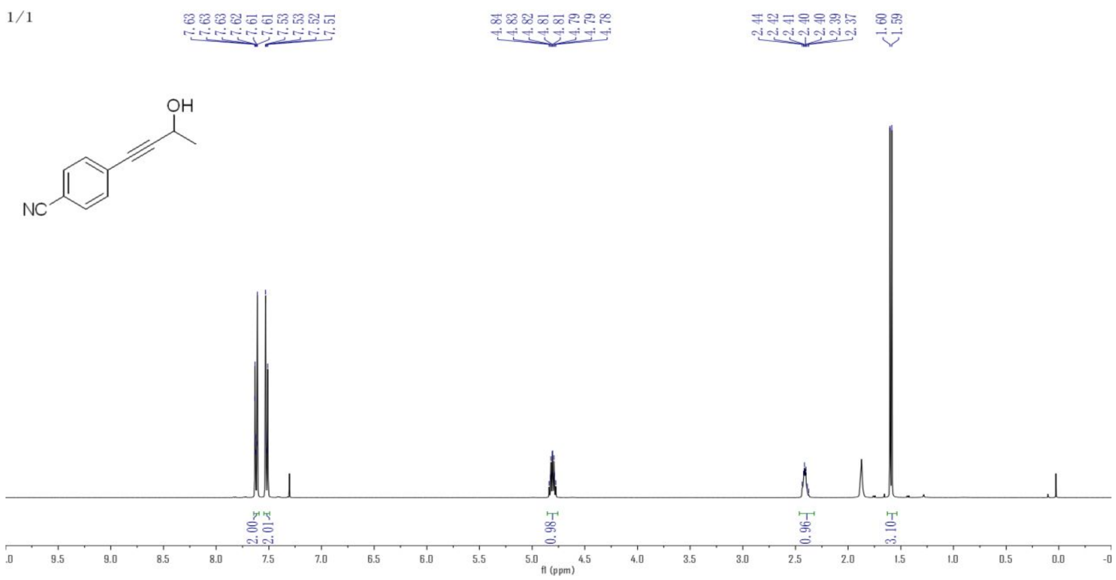

Figure S37. ^{1}H NMR (400 MHz) spectrum of racemic 4-(3-hydroxybut-1-yn-1-yl)benzonitrile (**11a**) in $CDCl_3$.

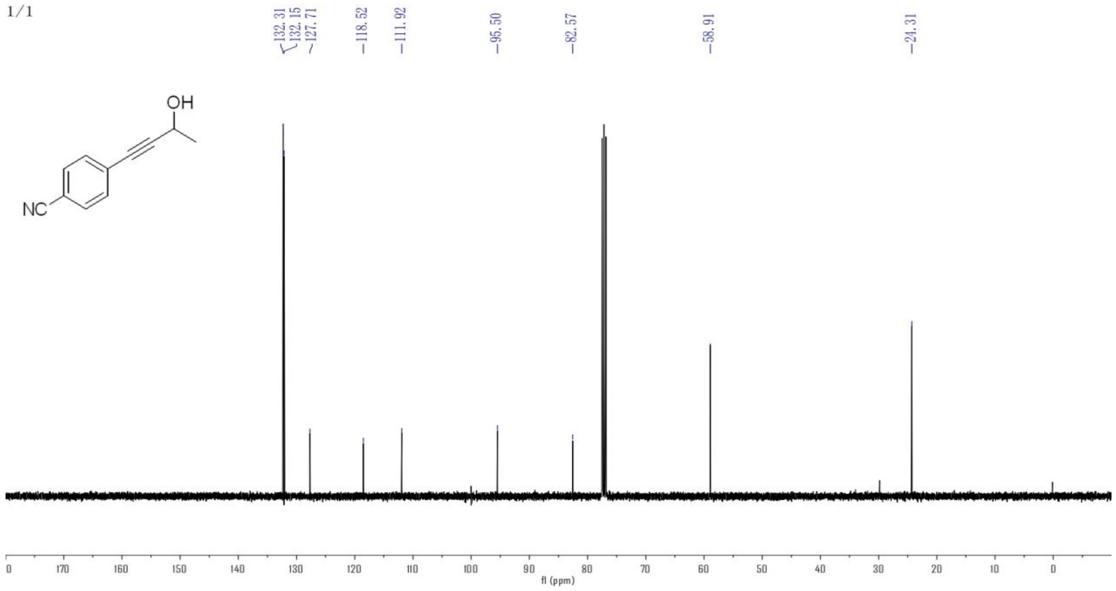

Figure S38. ^{13}C NMR (100 MHz) spectrum of racemic 4-(3-hydroxybut-1-yn-1-yl)benzonitrile (**11a**) in $CDCl_3$.

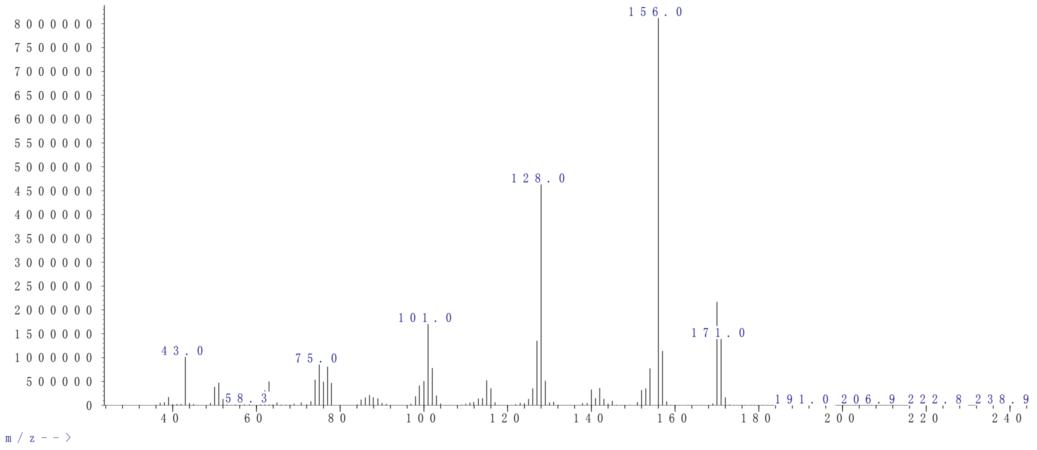

Figure S39. GC-MS spectrum of racemic 4-(3-hydroxybut-1-yn-1-yl)benzonitrile (**11a**).

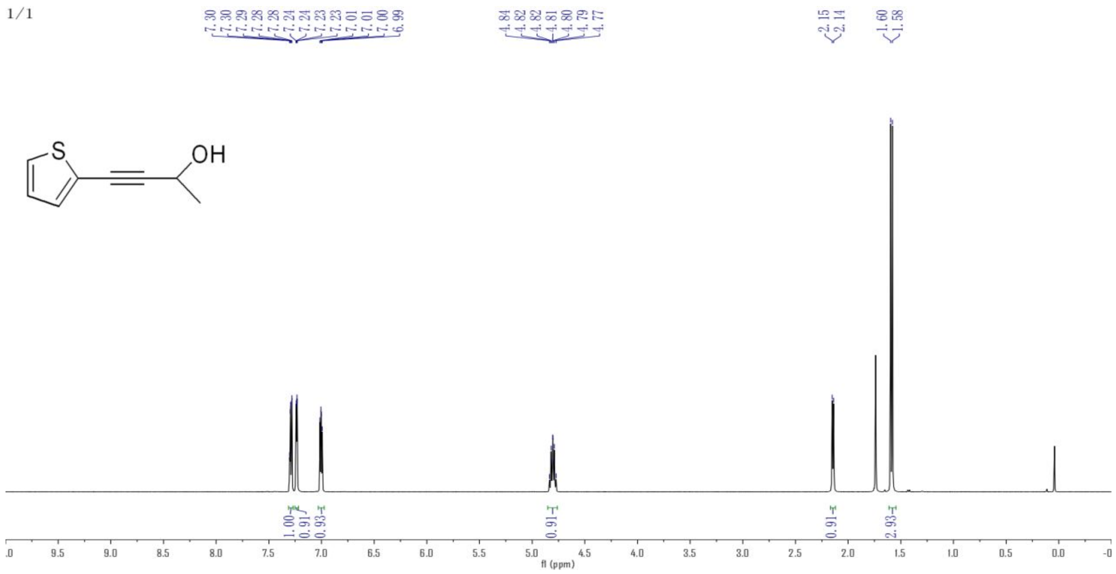

Figure S40. ^{1}H NMR (400 MHz) spectrum of racemic 4-(Thiophen-2-yl)but-3-yn-2-ol (**12a**) in $CDCl_3$.

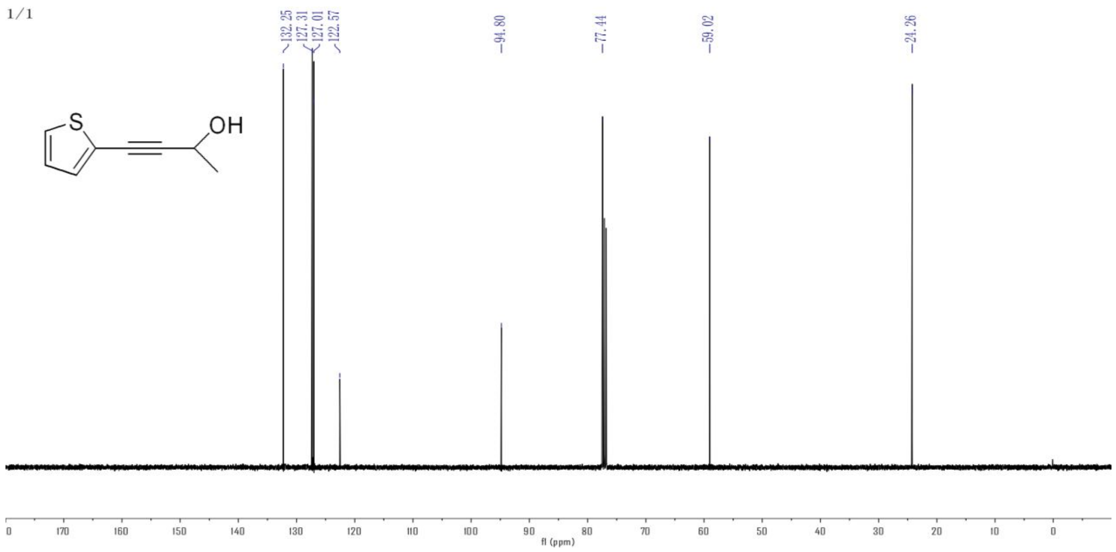

Figure S41. ^{13}C NMR (100 MHz) spectrum of racemic 4-(Thiophen-2-yl)but-3-yn-2-ol (**12a**) in $CDCl_3$.

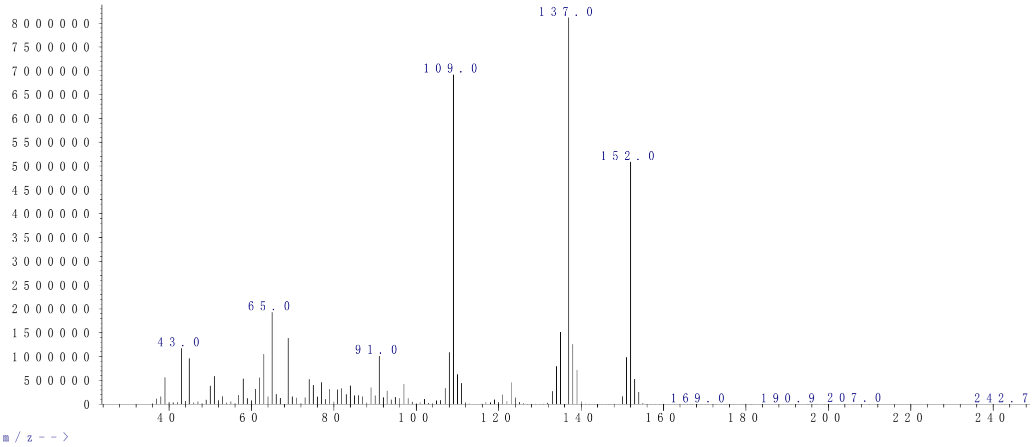

Figure S42. GC-MS spectrum of racemic 4-(Thiophen-2-yl)but-3-yn-2-ol (**12a**).

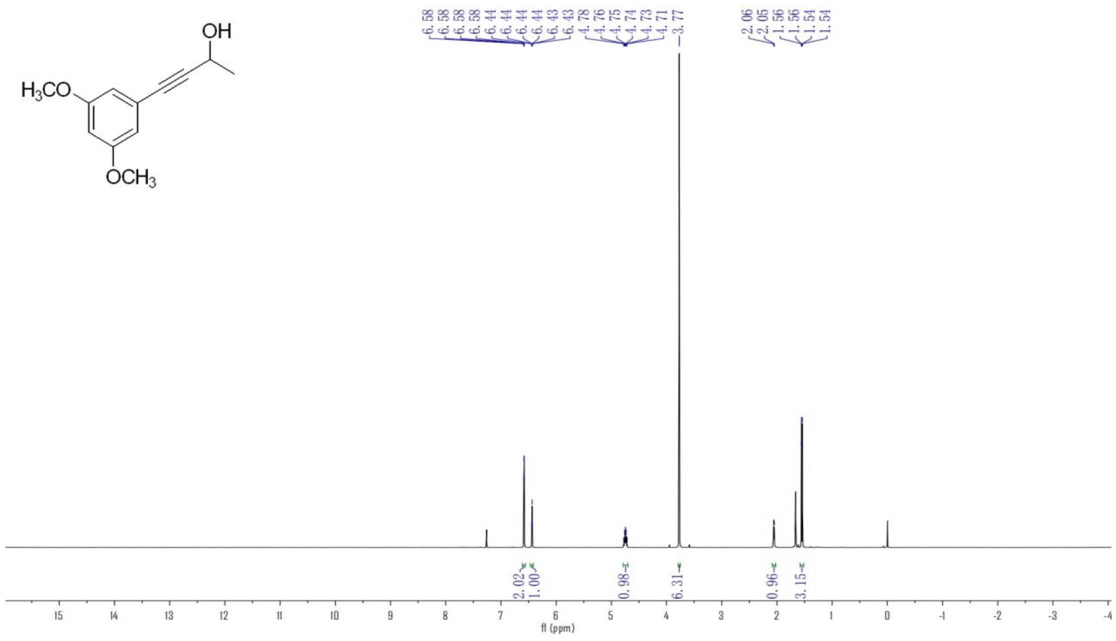

Figure S43. ^{1}H NMR (400 MHz) spectrum of racemic 4-(3,5-Dimethoxyphenyl)but-3-yn-2-ol (**13a**) in $CDCl_3$.

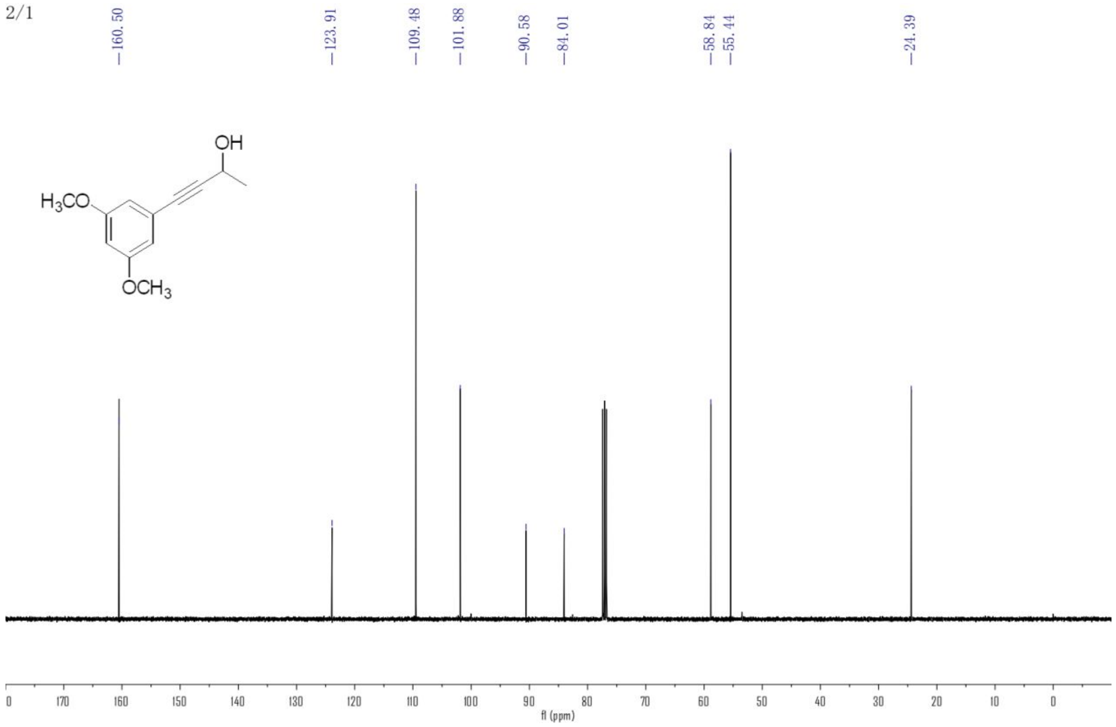

Figure S44. ^{13}C NMR (100 MHz) spectrum of racemic 4-(3,5-Dimethoxyphenyl)but-3-yn-2-ol (**13a**) in $CDCl_3$.

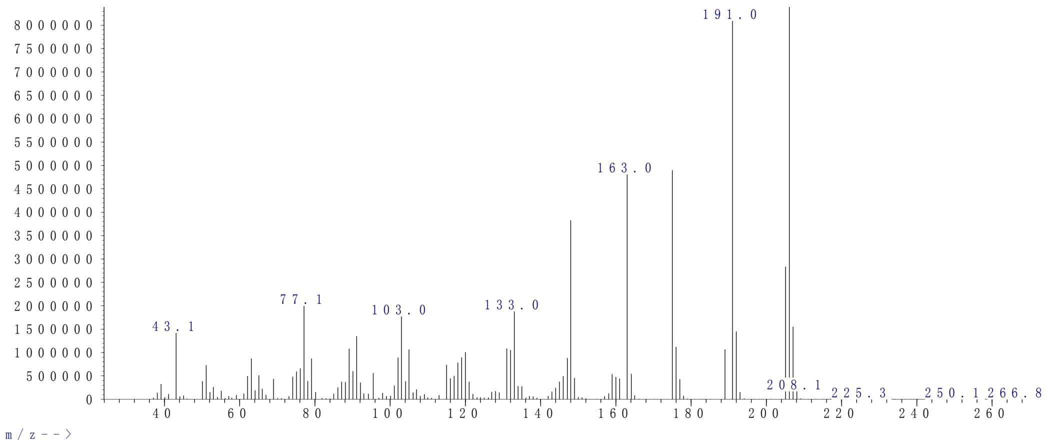

Figure S45. GC-MS spectrum of racemic 4-(3,5-Dimethoxyphenyl)but-3-yn-2-ol (**13a**).

4-(4-(Trifluoromethyl)phenyl)but-3-yn-2-ol

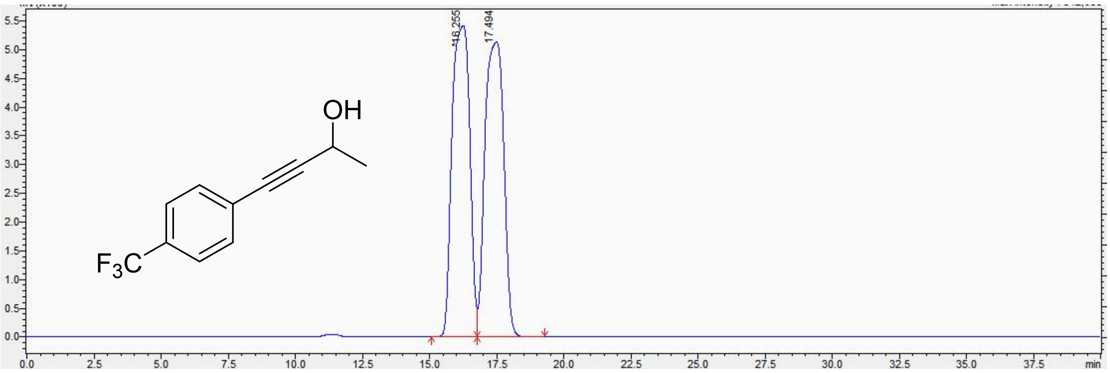

Peak	Retention time (min)	Peak area (mV*min)	Peak height (mV)	Peak area (%)
1	16.255	24951392	541962	49.805
2	17.494	25146735	513610	50.195

Figure S46. Representative HPLC chromatogram of racemic **2a** .

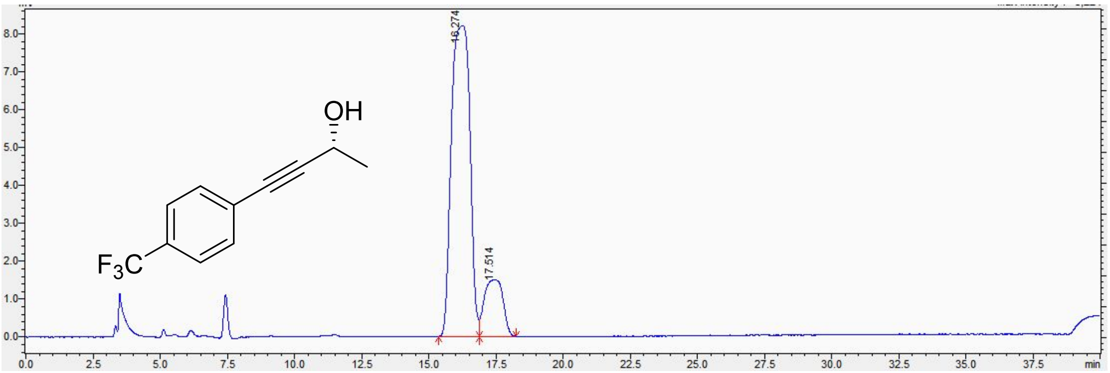

Peak	Retention time (min)	Peak area (mV*min)	Peak height (mV)	Peak area (%)
1	16.274	395651	8214	84.383
2	17.514	73223	1489	15.617

Figure S47. Representative HPLC chromatogram of (*R*)-**2a.**

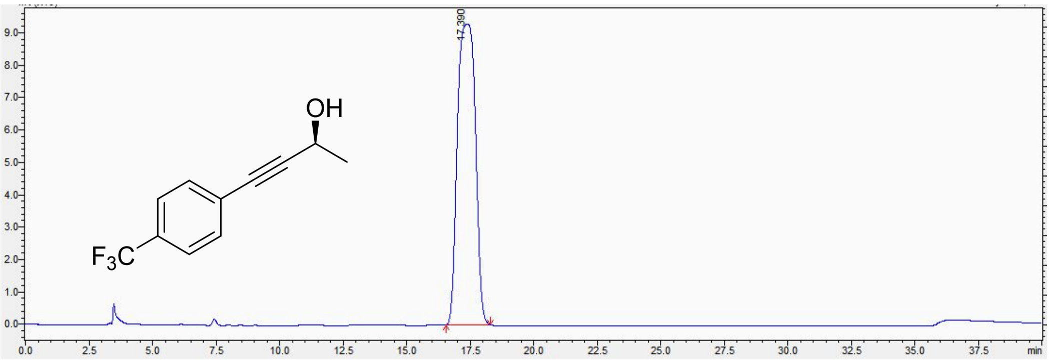

Peak	Retention time (min)	Peak area (mV*min)	Peak height (mV)	Peak area (%)
1	17.390	45833357	92960	100

Figure S48. Representative HPLC chromatogram of (*S*)-**2a** respectively.

4-(4-Methoxyphenyl)but-3-yn-2-ol

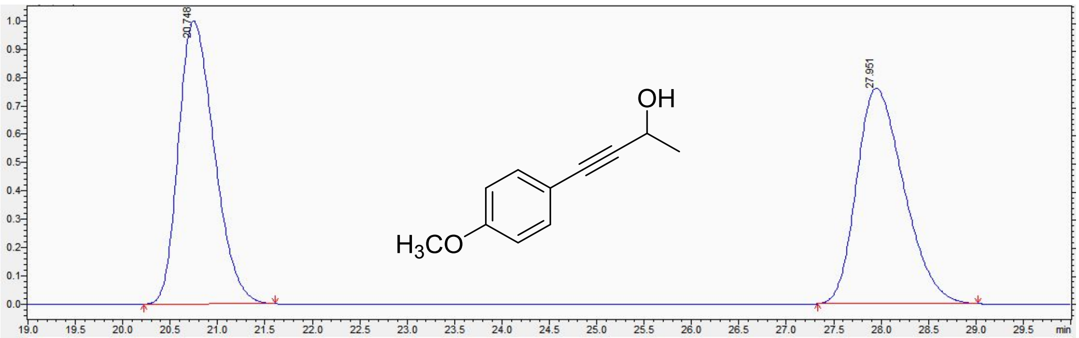

Peak	Retention time (min)	Peak area (mV*min)	Peak height (mV)	Peak area (%)
1	20.748	26295333	997952	49.900
2	27.951	26401168	759801	50.100

Figure S49. Representative HPLC chromatogram of racemic **3a**.

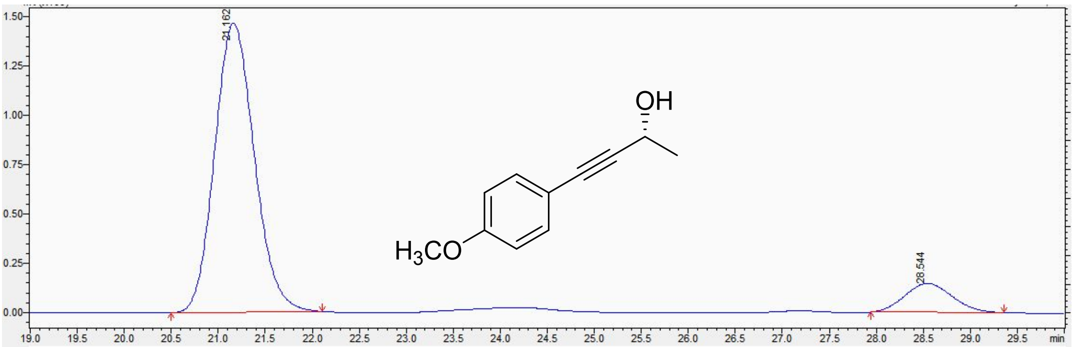

Peak	Retention time (min)	Peak area (mV*min)	Peak height (mV)	Peak area (%)
1	21.162	4307307	146518	89.335
2	28.544	514214	14623	10.665

Figure S50. Representative HPLC chromatogram of (*R*)-**3a**.

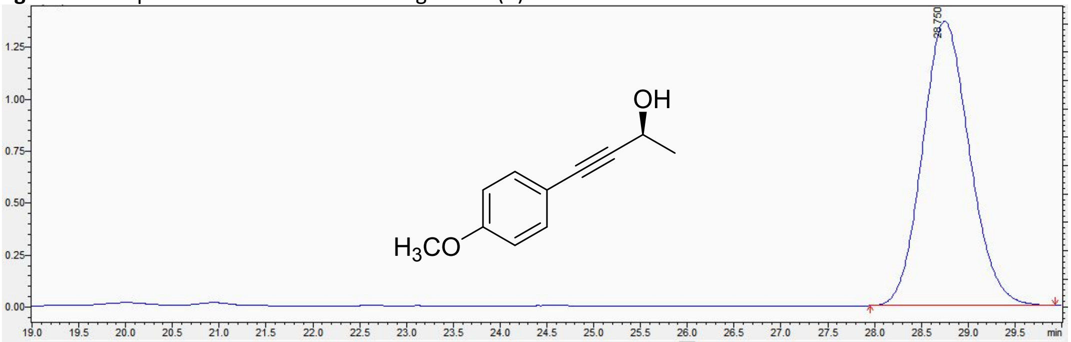

Peak	Retention time (min)	Peak area (mV*min)	Peak height (mV)	Peak area (%)
1	28.750	479795	13693	100.000

Figure S51. Representative HPLC chromatogram of (*S*)-**3a**

4-(4'-nitrophenyl)-3-butyn-2-ol

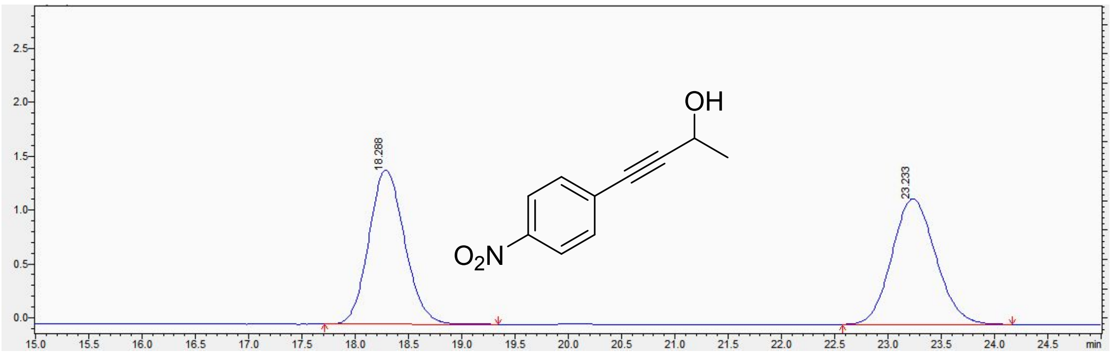

Peak	Retention time (min)	Peak area (mV*min)	Peak height (mV)	Peak area (%)
1	18.288	334243	14323	50.029
2	23.233	333851	11669	49.971

Figure S52. Representative HPLC chromatogram of racemic **4a**.

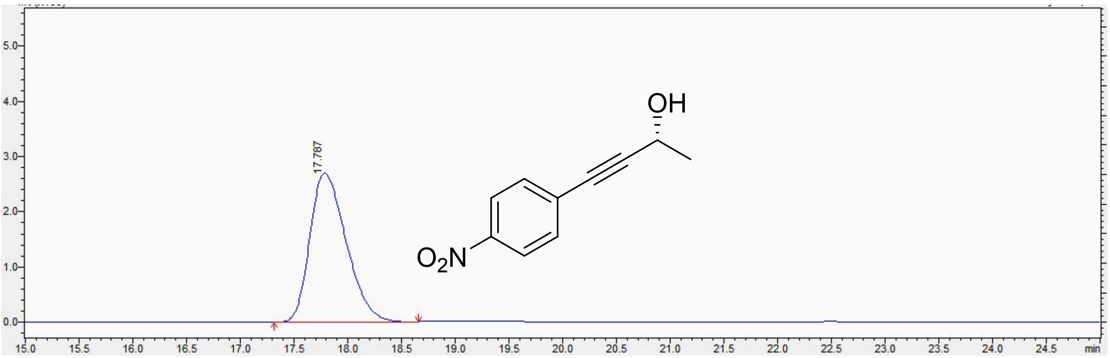

Peak	Retention time (min)	Peak area (mV*min)	Peak height (mV)	Peak area (%)
1	17.787	6320214	270094	100.000

Figure S53. Representative HPLC chromatogram of (*R*)-**4a**.

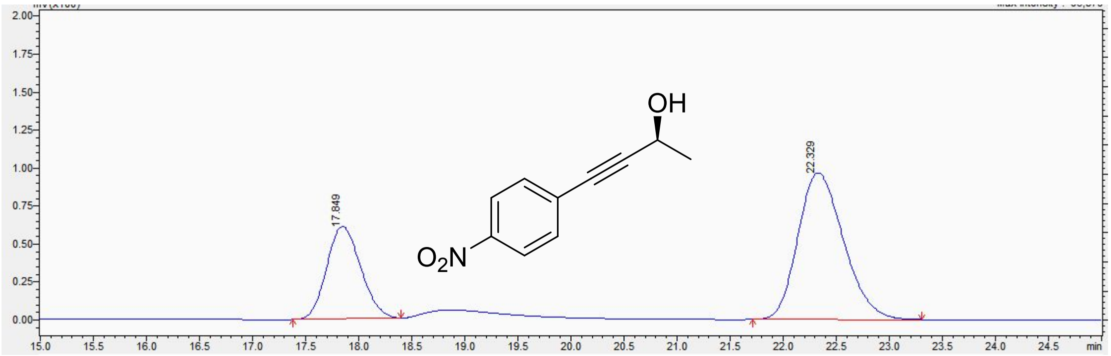

Peak	Retention time (min)	Peak area (mV*min)	Peak height (mV)	Peak area (%)
1	17.849	1324739	60712	31.982
2	22.329	2881212	96483	68.018

Figure S54. Representative HPLC chromatogram of (*S*)-**4a**.

4-(2-Methoxyphenyl)but-3-yn-2-ol

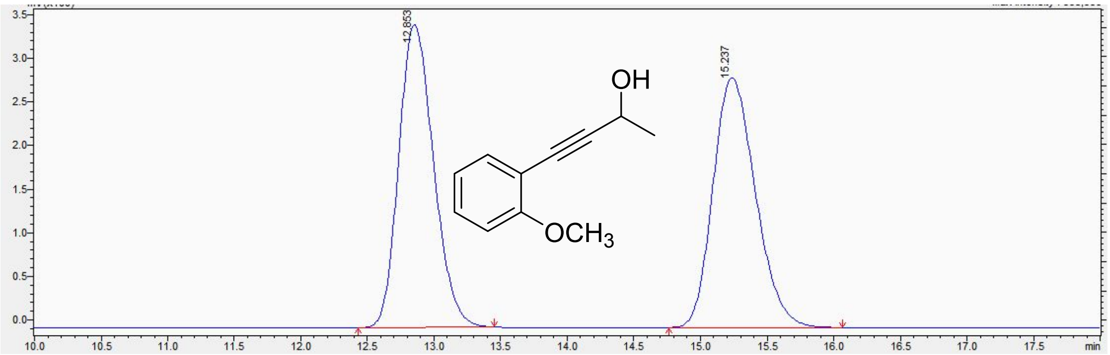

Peak	Retention time (min)	Peak area (mV*min)	Peak height (mV)	Peak area (%)
1	12.853	6307749	347083	49.909
2	15.237	6330771	286177	50.091

Figure S55. Representative HPLC chromatogram of racemic **5a**.

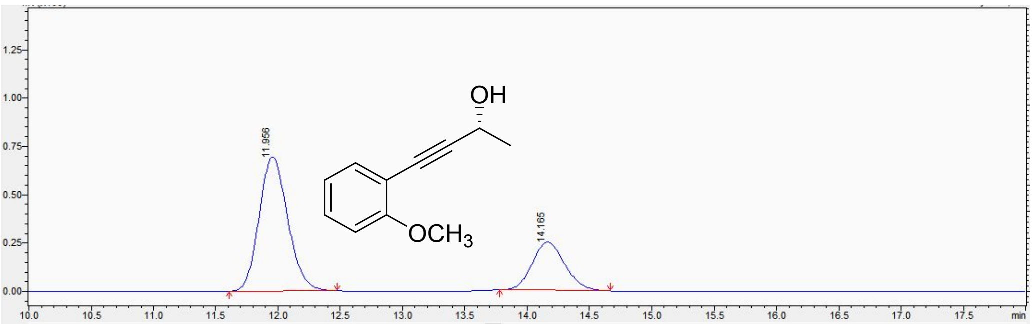

Peak	Retention time (min)	Peak area (mV*min)	Peak height (mV)	Peak area (%)
1	11.956	1111174	69434	70.958
2	14.165	454787	24889	29.042

Figure S56. Representative HPLC chromatogram of (*R*)-**5a**.

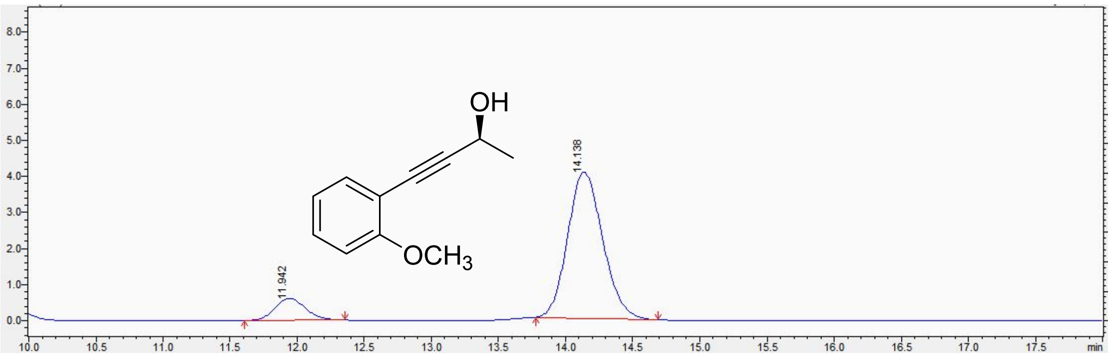

Peak	Retention time (min)	Peak area (mV*min)	Peak height (mV)	Peak area (%)
1	11.942	95410	6022	11.441
2	14.138	738541	40621	88.559

Figure S57. Representative HPLC chromatogram of (*S*)-**5a**.

4-Phenylbut-3-yn-2-ol

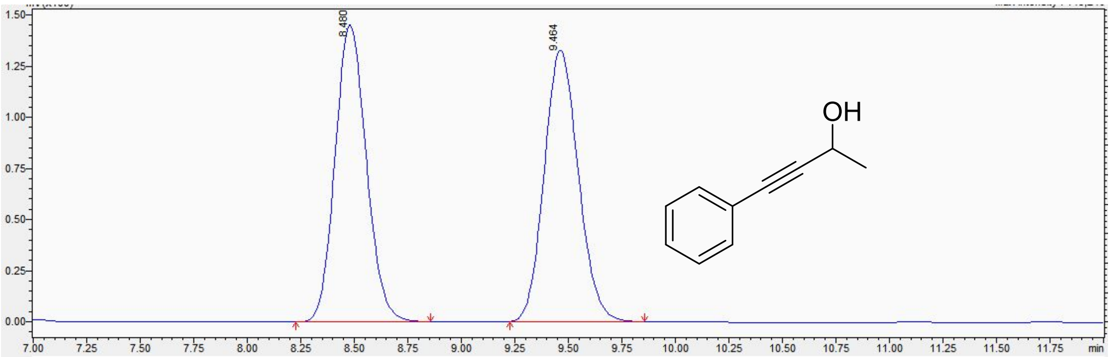

Peak	Retention time (min)	Peak area (mV*min)	Peak height (mV)	Peak area (%)
1	8.480	1476200	145325	49.990
2	9.464	1476818	132771	20.010

Figure S58. Representative HPLC chromatogram of racemic **6a**.

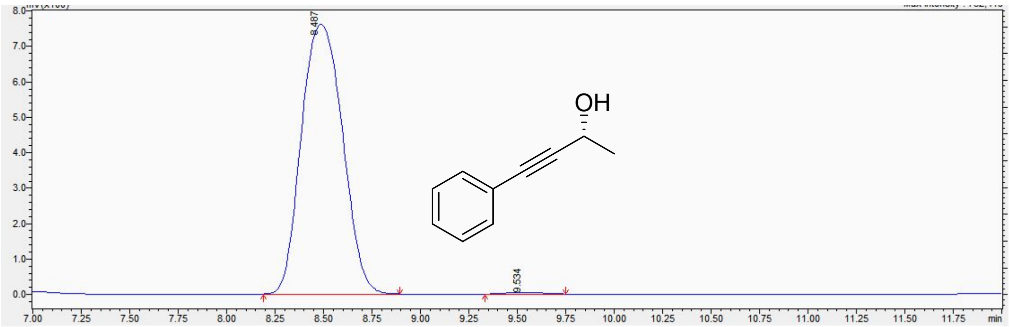

Peak	Retention time (min)	Peak area (mV*min)	Peak height (mV)	Peak area (%)
1	8.487	11286912	761133	99.469
2	9.534	60300	4448	0.531

Figure S59. Representative HPLC chromatogram of (*R*)-**6a**

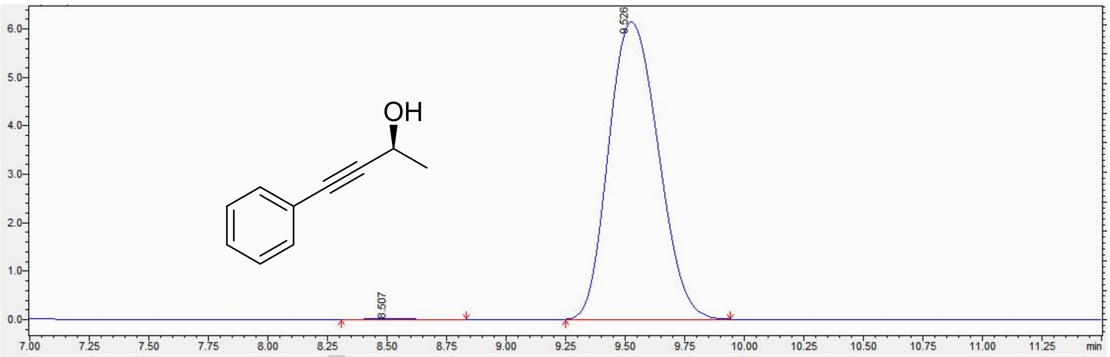

Peak	Retention time (min)	Peak area (mV*min)	Peak height (mV)	Peak area (%)
1	8.507	24618	2034	0.276
2	9.526	8893529	613741	99.724

Figure S60. Representative HPLC chromatogram of (*S*)-**6a.**

4-(4-chlorophenyl)-3-butyn-2-ol

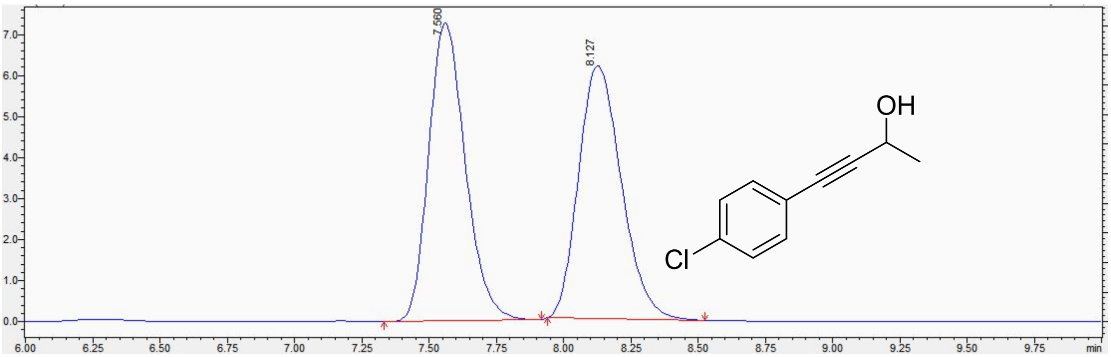

Peak	Retention time (min)	Peak area (mV*min)	Peak height (mV)	Peak area (%)
1	7.560	6873112	726130	50.159
2	8.127	6829442	617268	49.841

Figure S61. Representative HPLC chromatogram of racemic **7a**.

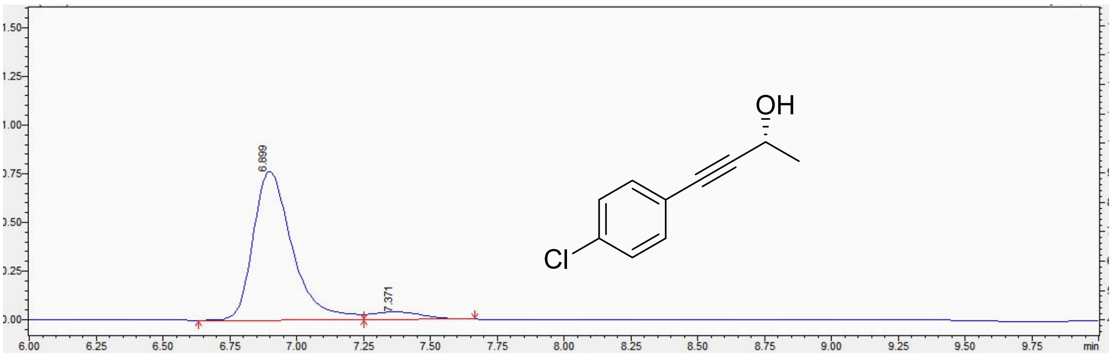

Peak	Retention time (min)	Peak area (mV*min)	Peak height (mV)	Peak area (%)
1	6.899	776198	76350	93.861
2	7.371	50769	80232	6.139

Figure S62. Representative HPLC chromatogram of (*R*)-**7a.**

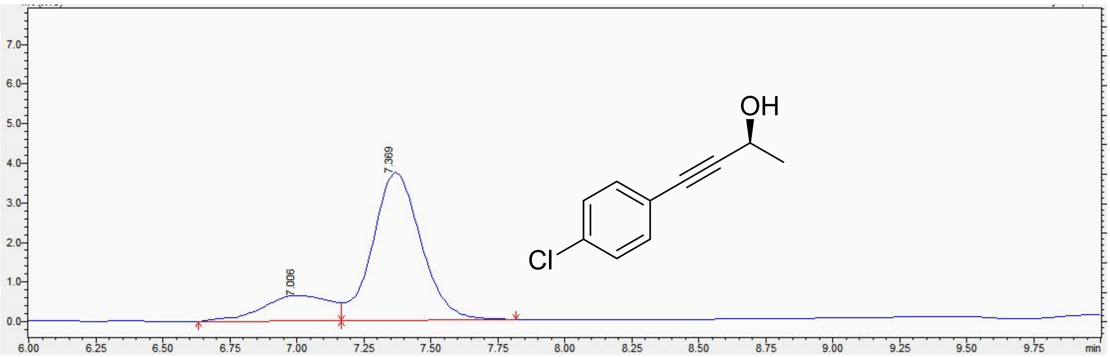

Peak	Retention time (min)	Peak area (mV*min)	Peak height (mV)	Peak area (%)
1	7.006	113641	6350	19.644
2	7.369	464851	37229	80.356

Figure S63. Representative HPLC chromatogram of (*S*)-**7a**.

4-(4-bromophenyl)-3-butyn-2-ol

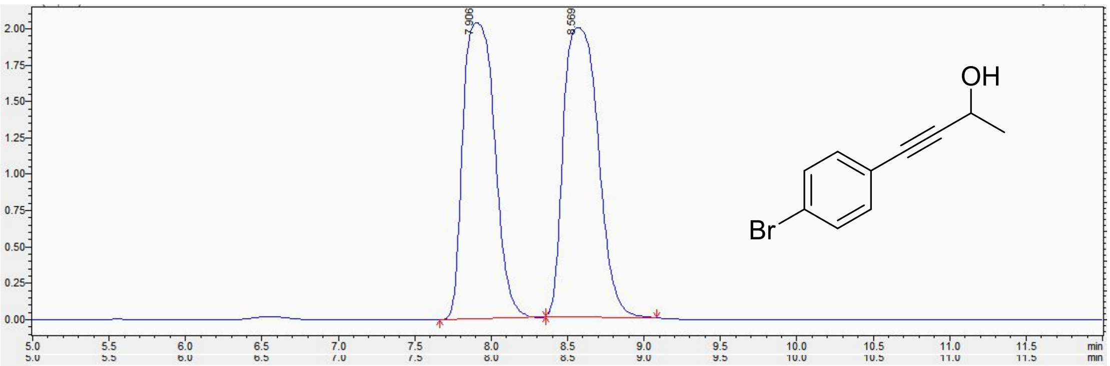

Peak	Retention time (min)	Peak area (mV*min)	Peak height (mV)	Peak area (%)
1	7.906	29244166	2032235	48.153
2	8.569	31487380	1987812	51.847

Figure S64. Representative HPLC chromatogram of racemic **8a**.

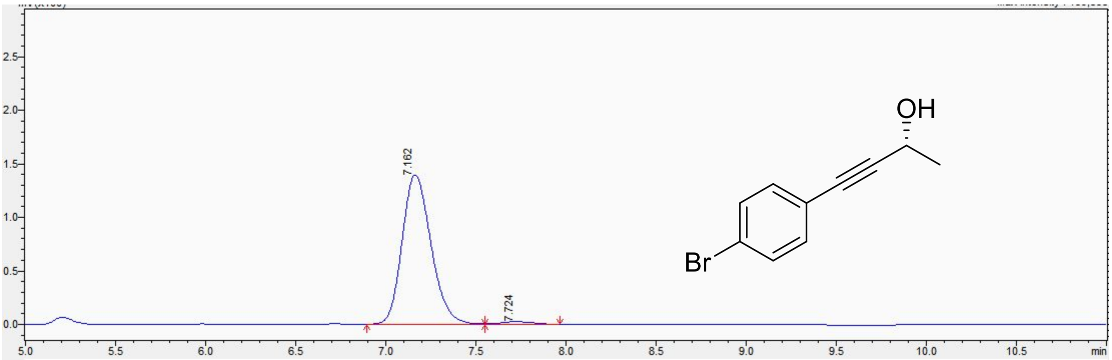

Peak	Retention time (min)	Peak area (mV*min)	Peak height (mV)	Peak area (%)
1	7.162	1558654	139685	98.128
2	7.724	29727	2465	1.872

Figure S65. Representative HPLC chromatogram of (*R*)-**8a**.

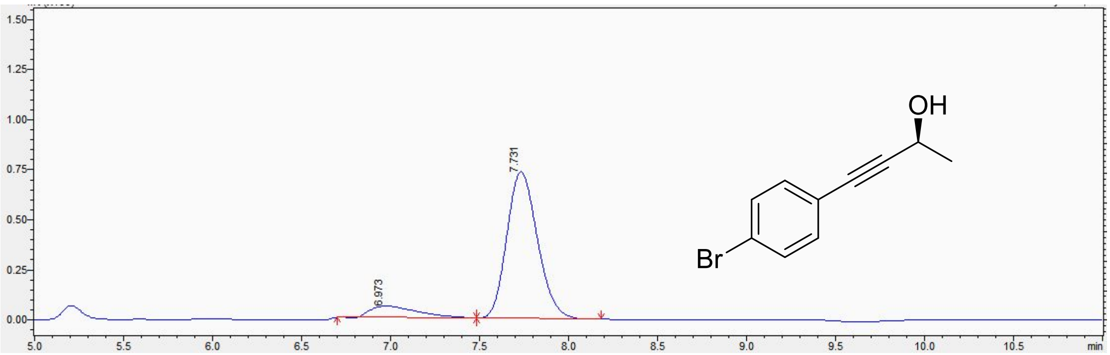

Peak	Retention time (min)	Peak area (mV*min)	Peak height (mV)	Peak area (%)
1	6.973	107021	5725	10.852
2	7.731	879160	73386	89.148

Figure S66. Representative HPLC chromatogram of (*S*)-**8a**.

4-(3,5-Dimethylphenyl)but-3-yn-2-ol

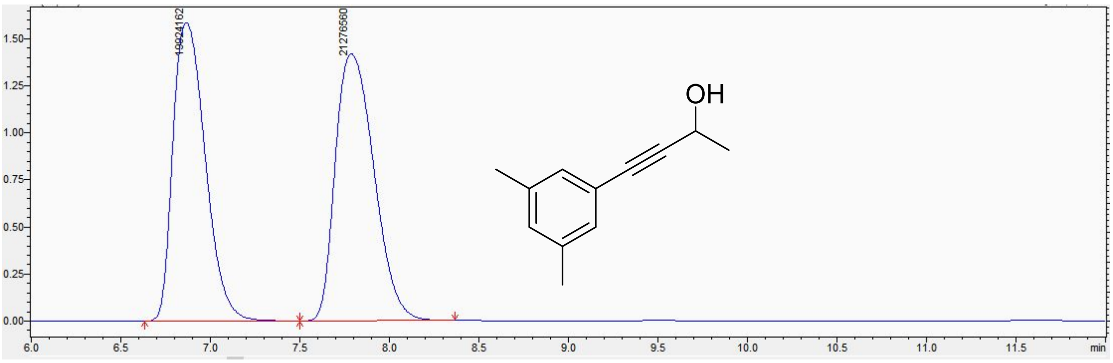

Peak	Retention time (min)	Peak area (mV*min)	Peak height (mV)	Peak area (%)
1	6.865	19924162	1583363	46.359
2	7.786	21276560	1416018	51.641

Figure S67. Representative HPLC chromatogram of racemic **9a**.

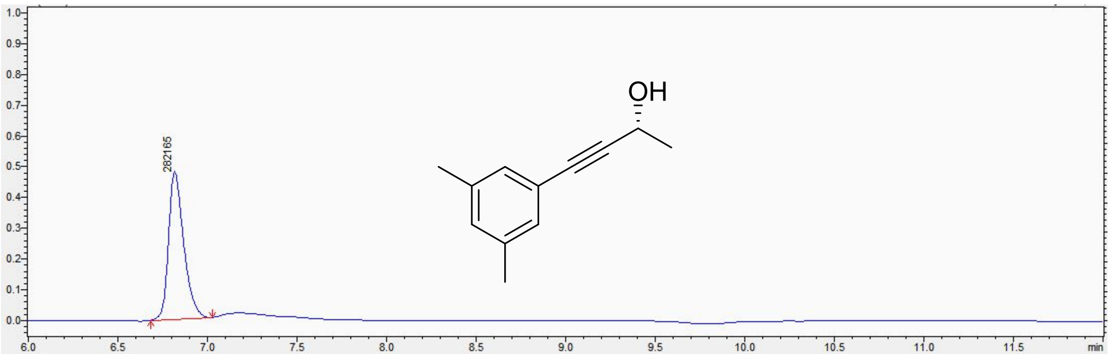

Peak	Retention time(min)	Peak area (mV*min)	Peak height (mV)	Peak area (%)
1	6.816	282165	47911	100.000

Figure S68. Representative HPLC chromatogram of (*R*)-**9a**.

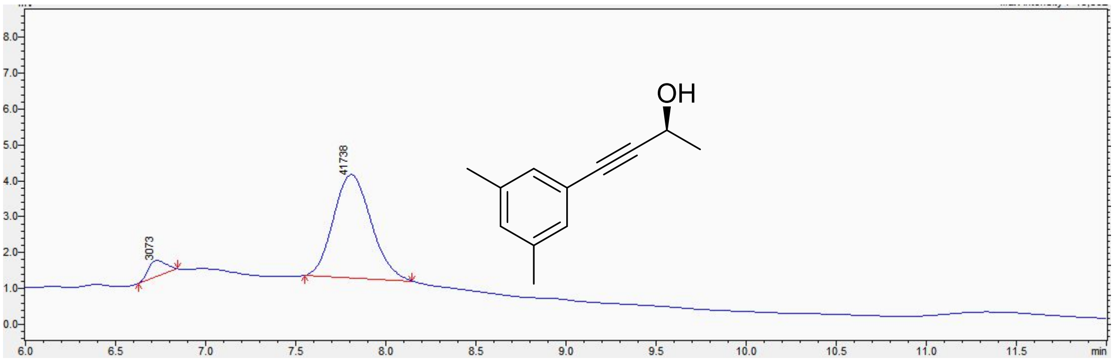

Peak	Retention time (min)	Peak area (mV*min)	Peak height (mV)	Peak area (%)
1	6.728	3073	437	6.858
2	7.806	41738	2867	93.142

Figure S69. Representative HPLC chromatogram of (*S*)-**9a**.

4-(3-(trifluoromethyl)phenyl)but-3-yn-2-ol

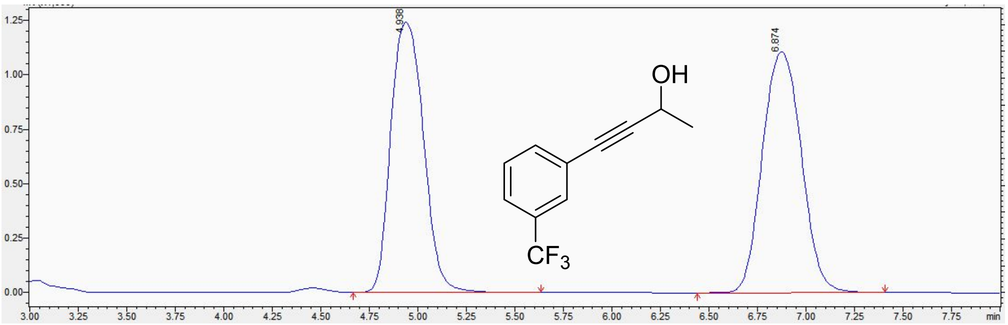

Peak	Retention time (min)	Peak area (mV*min)	Peak height (mV)	Peak area (%)
1	4.938	14576936	1243797	48.460
2	6.874	15503326	1107294	51.540

Figure S70. Representative HPLC chromatogram of racemic **10a**.

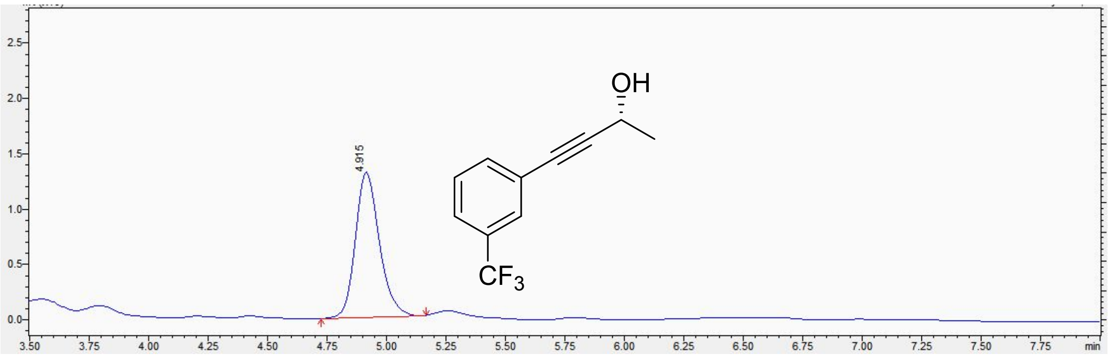

Peak	Retention time (min)	Peak area (mV*min)	Peak height (mV)	Peak area (%)
1	4.951	92101	13155	100.000

Figure S71. Representative HPLC chromatogram of (*R*)-**10a**.

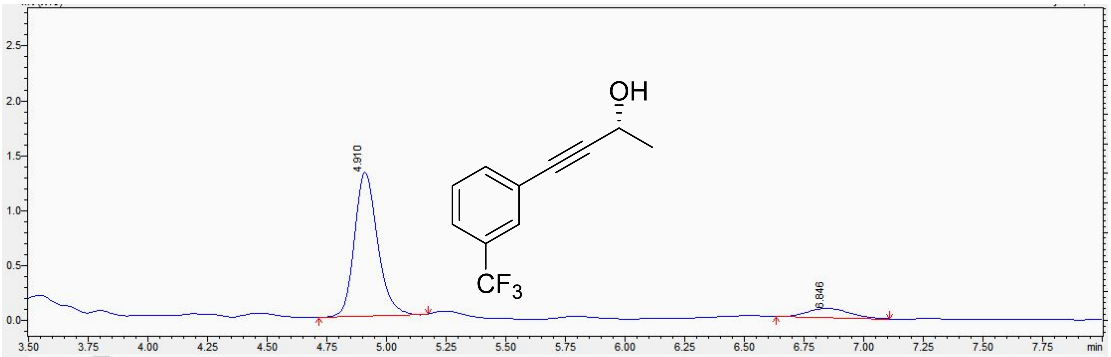

Peak	Retention time (min)	Peak area (mV*min)	Peak height (mV)	Peak area (%)
1	4.910	88075	13132	94.128
2	6.846	5405	598	5.782

Figure S72. Representative HPLC chromatogram of (*R*)-**10a**. Note: both *Lk*ADH and *Tb*ADH yielded the progargylic alcohol product in (*R*)-configuration.

4-(3-hydroxybut-1-yn-1-yl)benzonitrile

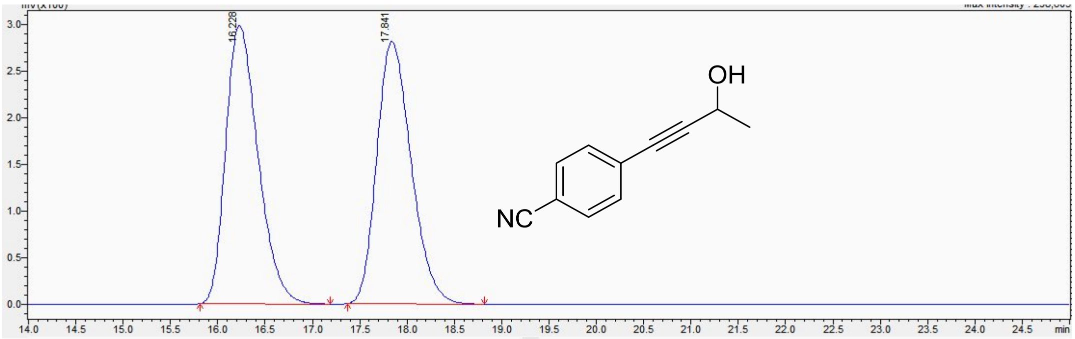

Peak	Retention time (min)	Peak area (mV*min)	Peak height (mV)	Peak area (%)
1	16.228	7087551	297794	49.936
2	17.841	7105579	280665	50.064

Figure S73. Representative HPLC chromatogram of racemic **11a**.

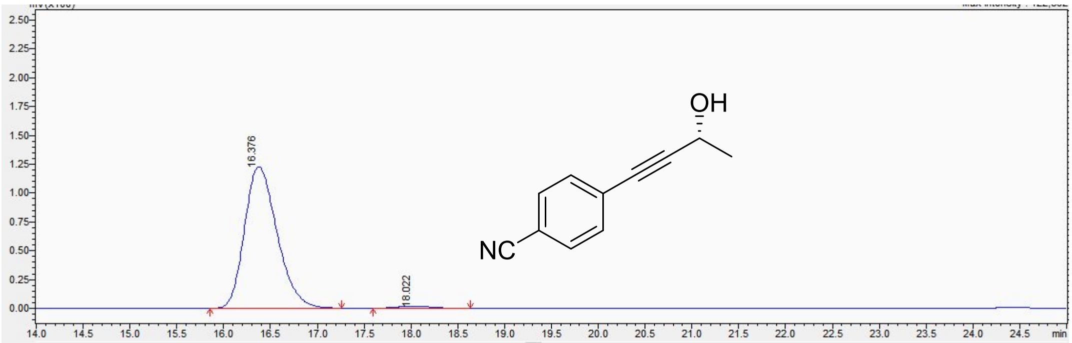

Peak	Retention time (min)	Peak area (mV*min)	Peak height (mV)	Peak area (%)
1	16.376	2964446	122762	98.486
2	18.022	45574	1860	1.514

Figure S74. Representative HPLC chromatogram of (*R*)-**11a**.

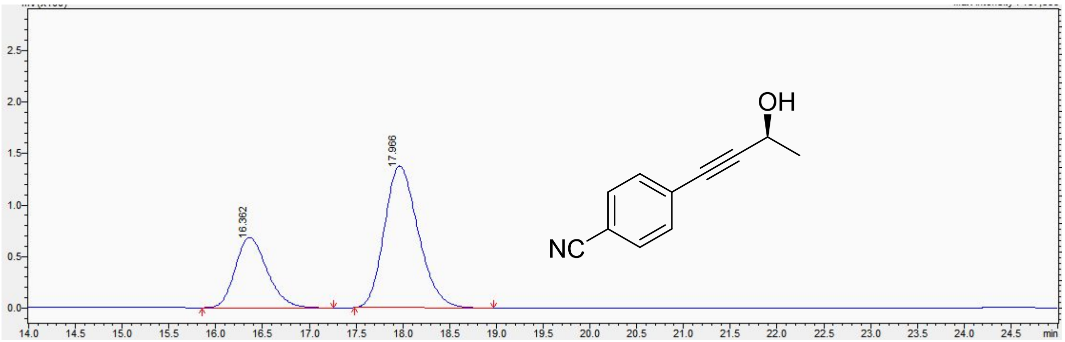

Peak	Retention time (min)	Peak area (mV*min)	Peak height (mV)	Peak area (%)
1	16.362	1622036	68227	31.839
2	17.966	3472499	137389	68.161

Figure S75. Representative HPLC chromatogram of (*S*)-**11a**.

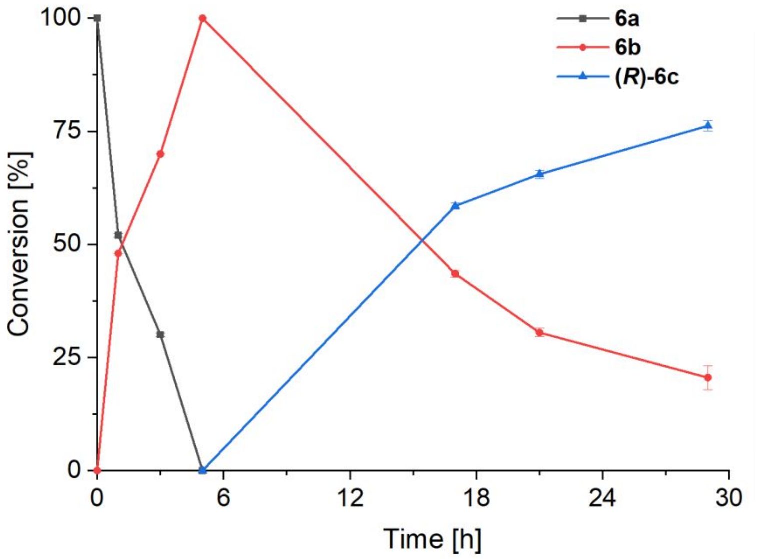

Figure S76. Representative time course of a one-pot two-step synthesis of ***(R)*-6c**. The r*Aae*UPO-catalysed oxidation step was performed first, followed by addition of the ATA, PLP and D-alanine after 5 h. Conditions: [substrate] = 5 mM, [r*Aae*UPO] = 2 µM; $[H_2O_2]_{final}$ = 10 mM added at 2 mM h^{-1} (for 5 h), [D-Alanine] = 1 M, NaPi buffer (100 mM, pH 8), 30 % (v/v) MeCN as cosolvent, [PLP] = 0.1 mM, [*At*ATA] = 80 µM, 30 °C, 24 h, 800 rpm.

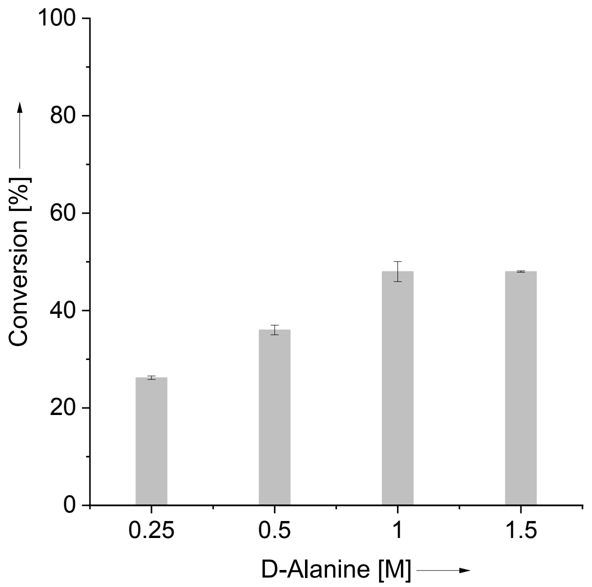

Figure S77. Influence of D-alanine concentrations on the conversion of **6b** to ***(R)*-6c**. Conditions: [D-Alanine] = 0.25-1.5 M, [substrate] = 5 mM, NaPi buffer (100 mM, pH 8), 30 % (v/v) MeCN as cosolvent, [PLP] = 0.1 mM, [*At*ATA] = 80 µM, 30 °C, 24 h, 800 rpm. The conversion was calculated by gas chromatography: [amine **6c**]$_{final}$ × ([ketone **6b**]$_{final}$ + [amine **6c**]$_{final}$)$^{-1}$.

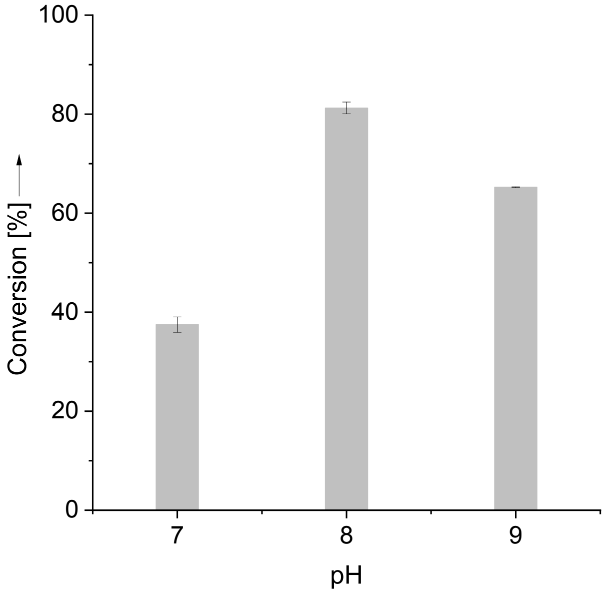

Figure S78. Influence of pH on the conversion of **6b** to **(*R*)-6c**. Conditions: [D-Alanine] = 1 M, [substrate] = 5 mM, NaPi buffer (100 mM, pH 7-9), 30 % (v/v) MeCN as cosolvent, [PLP] = 0.1 mM, [*At*ATA] = 80 µM, 30 ^{o}C, 24 h, 800 rpm. The conversion was calculated by gas chromatography**:** The conversion was calculated by gas chromatography: [amine **6c**]$_{final}$ $\times$ ([ketone **6b**]$_{final}$ + [amine **6c**]$_{final}$)$^{-1}$**.**

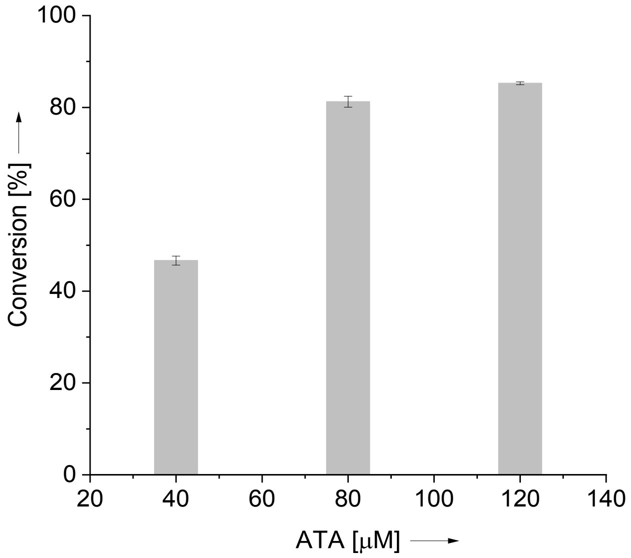

Figure S79. Influence of the enzyme concentration on the conversion of **6b** to (***R***)**-6c**. Conditions: [D-Alanine] = 1 M, [substrate] = 5 mM, NaPi buffer (100 mM, pH 8), 30 % (v/v) MeCN as cosolvent, [PLP] = 0.1 mM, [*At*ATA] = 40-120 µM, 30 °C, 24 h, 800 rpm. The conversion was calculated by gas chromatography: [amine **6c**]$_{final}$ × ([ketone **6b**]$_{final}$ + [amine **6c**]$_{final}$)$^{-1}$.

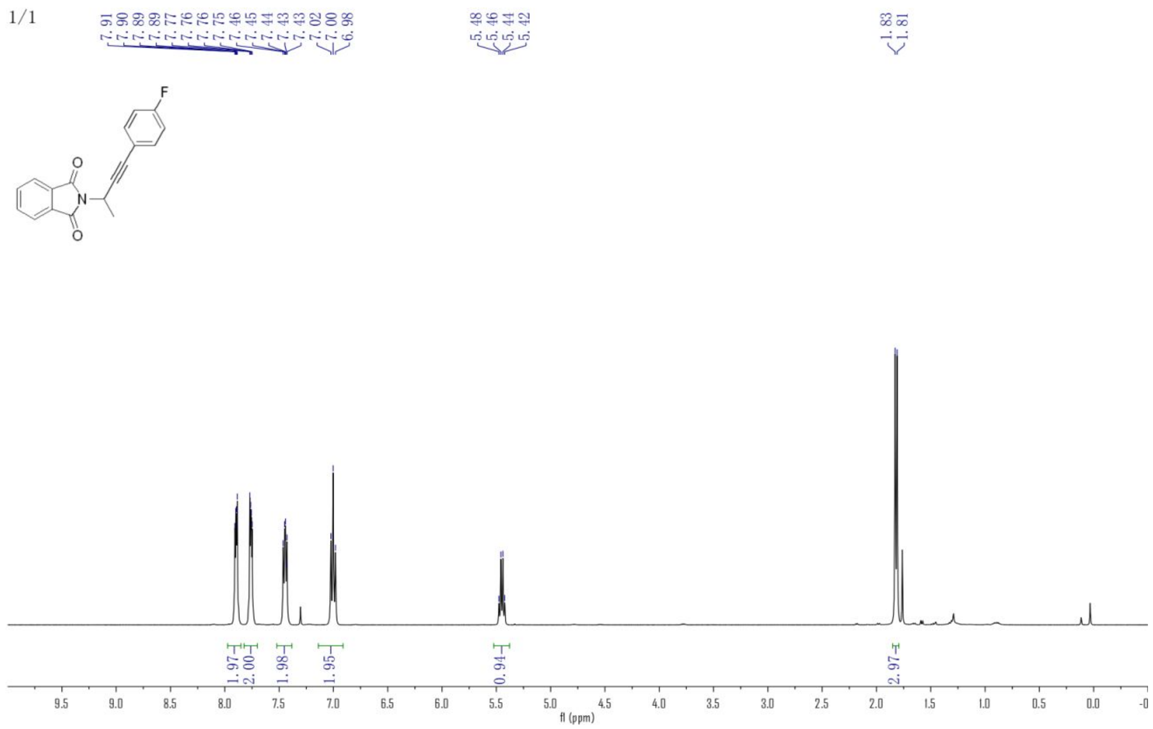

Figure S80. ^{1}H NMR (400 MHz) spectrum of the intermediate 2-(4-(4-fluorophenyl)but-3-yn-2-yl)isoindoline- 1,3-dione as the precursor for racemic (**1c**) in $CDCl_3$.

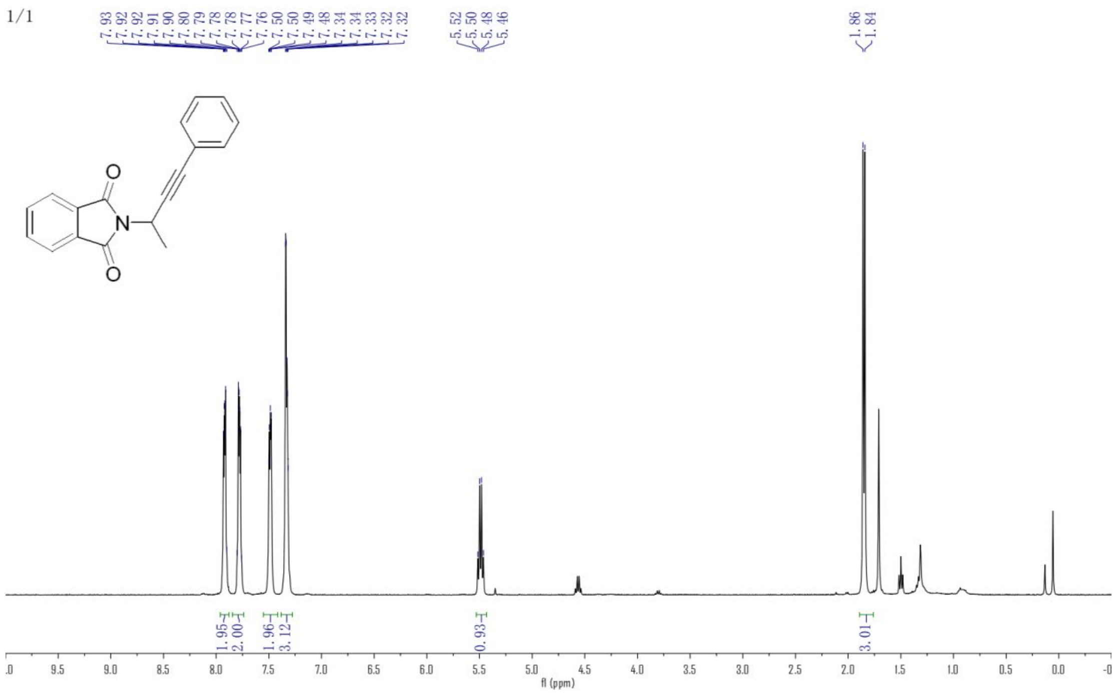

Figure S81. ^{1}H NMR (400 MHz) spectrum of the intermediate 2-(4-Phenylbut-3-yn-2-yl)isoindoline-1,3-dione as the precursor for racemic (**6c**) in $CDCl_3$.

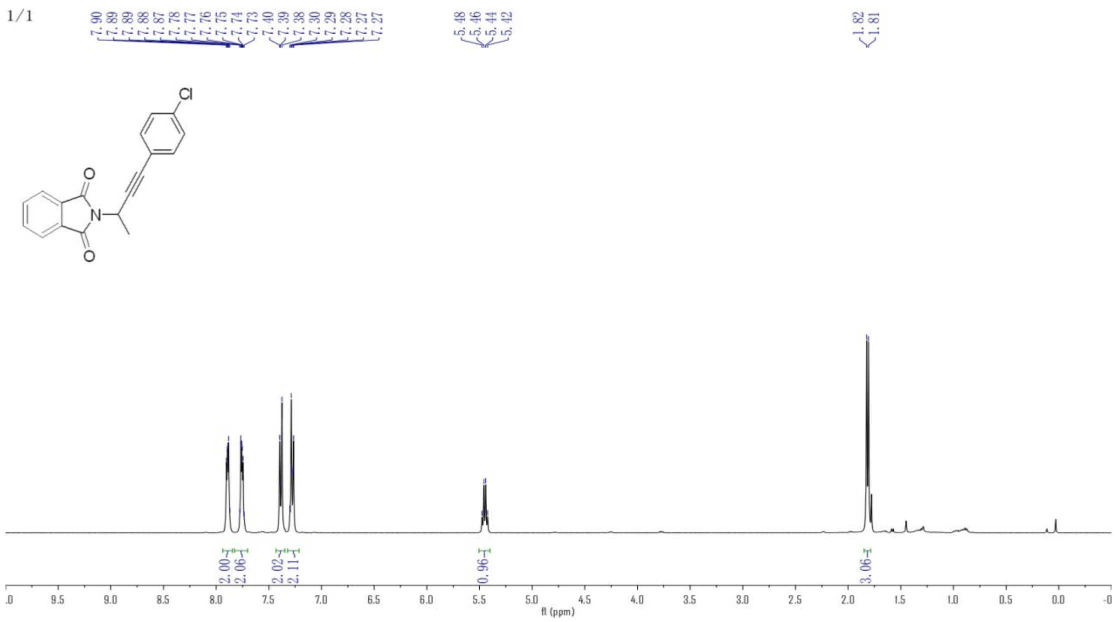

Figure S82. ^{1}H NMR (400 MHz) spectrum of the intermediate 2-(4-(4-chlorophenyl)but-3-yn-2-yl)isoindoline- 1,3-dione as the precursor for racemic (**7c**) in $CDCl_3$.

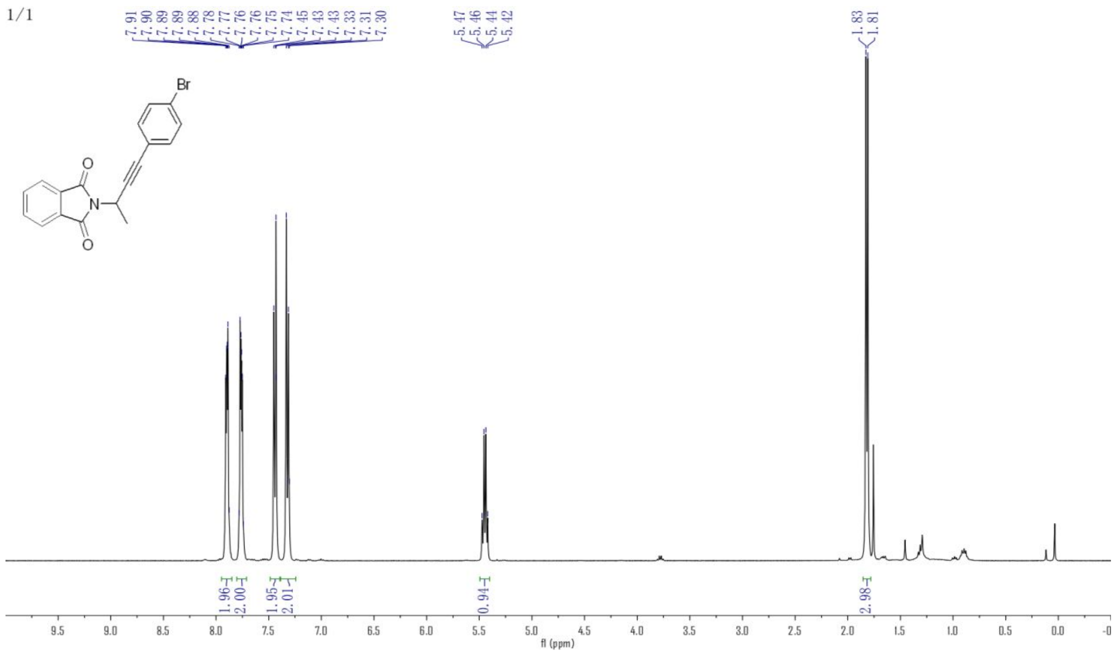

Figure S83. ^{1}H NMR (400 MHz) spectrum of the intermediate 2-(4-(4-bromophenyl)but-3-yn-2-yl)isoindoline- 1,3 -dione as the precursor for racemic (**8c**) in $CDCl_3$.

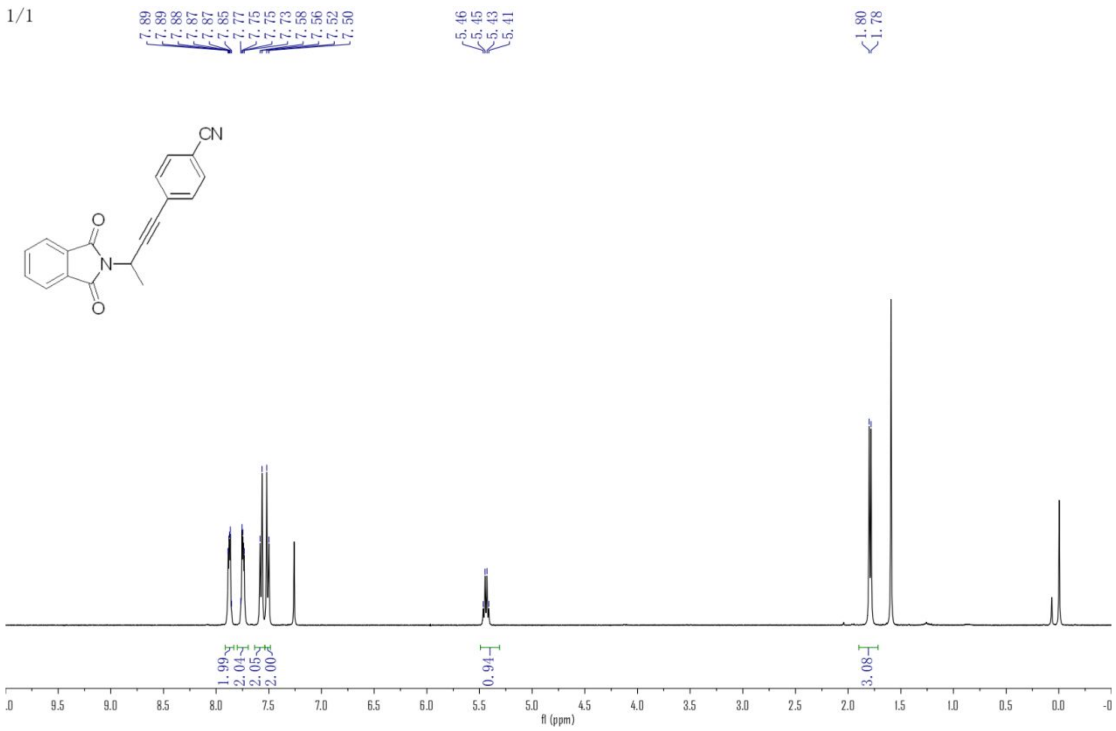

Figure S84. ^1H NMR (400 MHz) spectrum of the intermediate 2-(3-(1,3-dioxoisoindolin-2-yl)but-1-yn-1-yl) benzonitrile as the precursor for racemic (**11c**) **i**n $CDCl_3$.

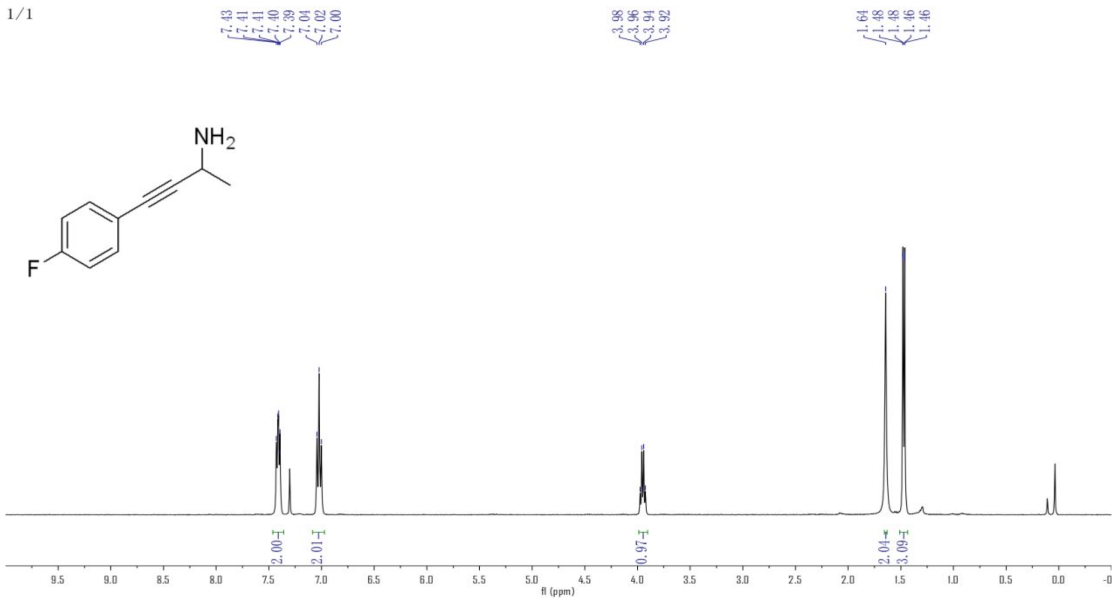

Figure S85. ^{1}H NMR (400 MHz) spectrum of racemic 4-(4-fluorophenyl)but-3-yn-2-amine (**1c**) in $CDCl_3$.

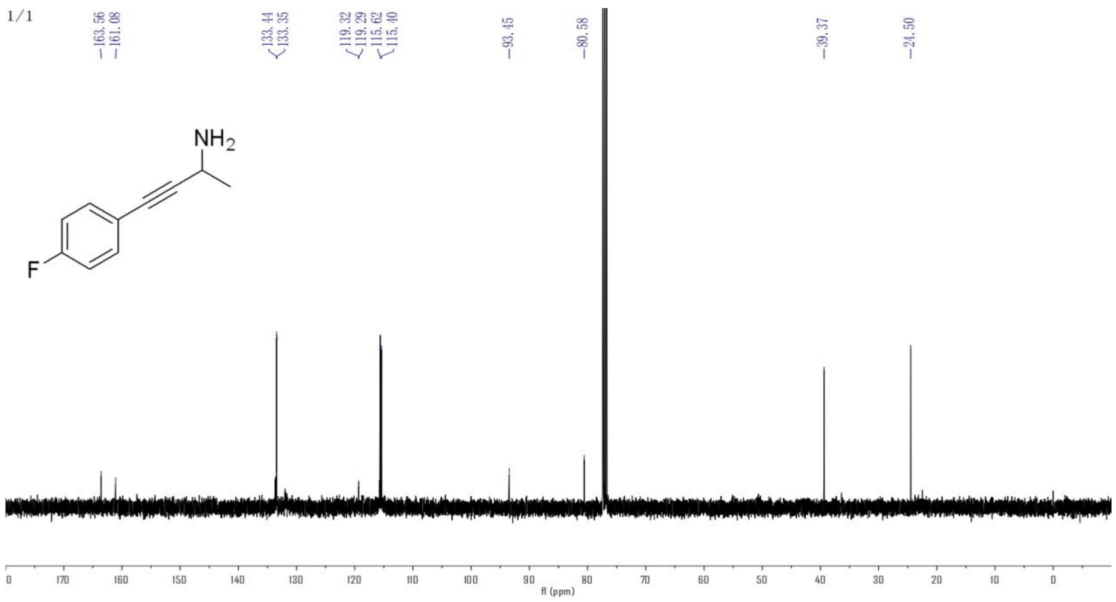

Figure S86. ^{13}C NMR (100 MHz) spectrum of racemic 4-(4-fluorophenyl)but-3-yn-2-amine (**1c**) in $CDCl_3$.

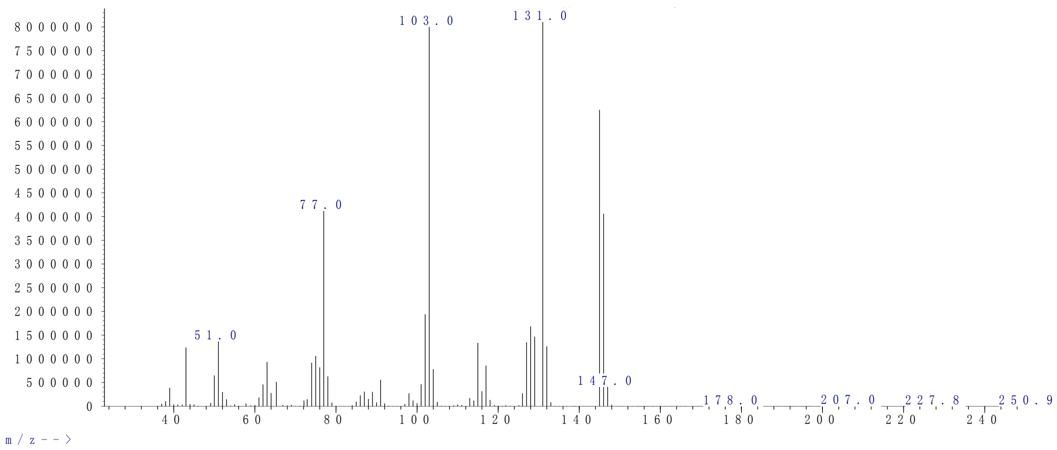

Figure S87. GC-MS spectrum of racemic 4-(4-fluorophenyl)but-3-yn-2-amine (**1c**).

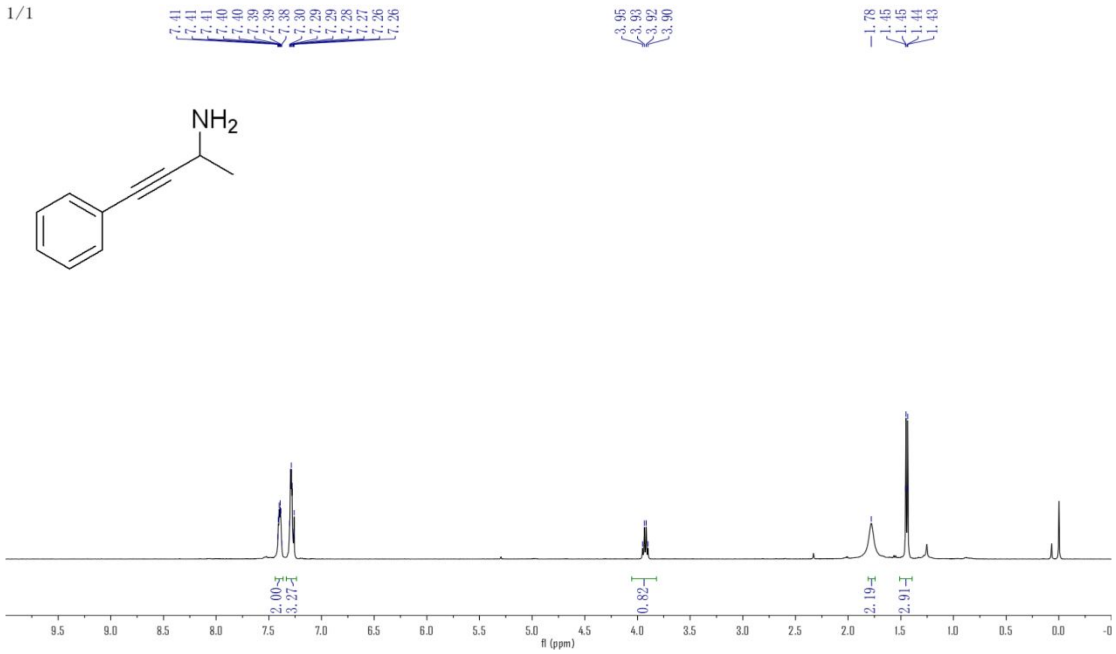

Figure S88. ^1H NMR (400 MHz) spectrum of racemic 4-phenylbut-3-yn-2-amine (**6c**) in $CDCl_3$.

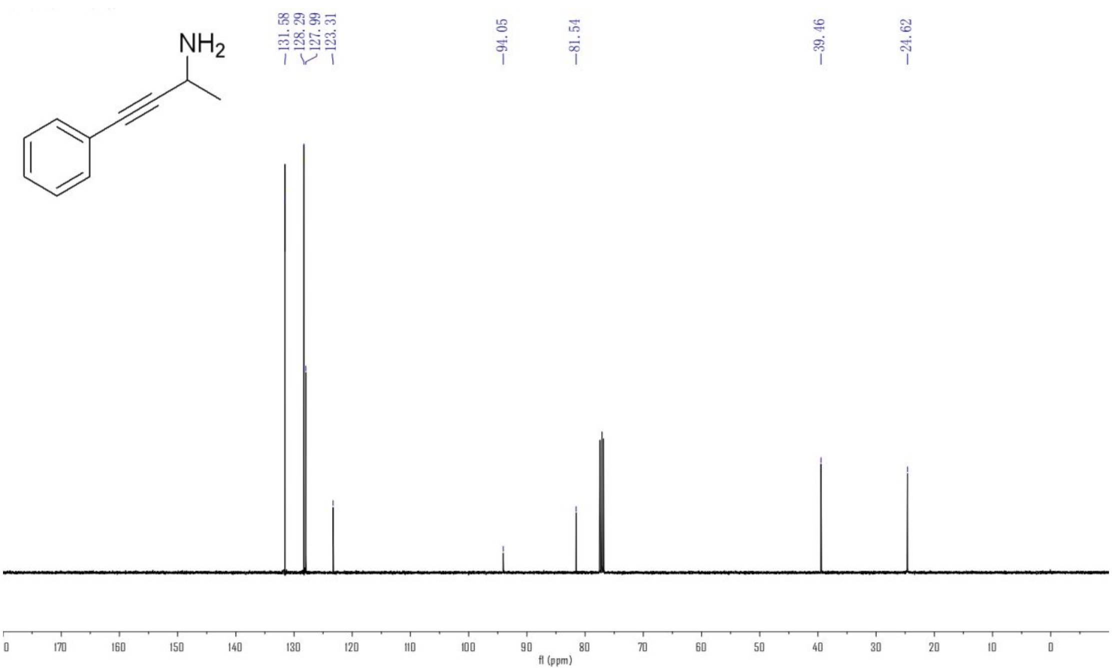

Figure S89. ^{13}C NMR (100 MHz) spectrum of racemic 4-phenylbut-3-yn-2-amine (**6c**) in $CDCl_3$.

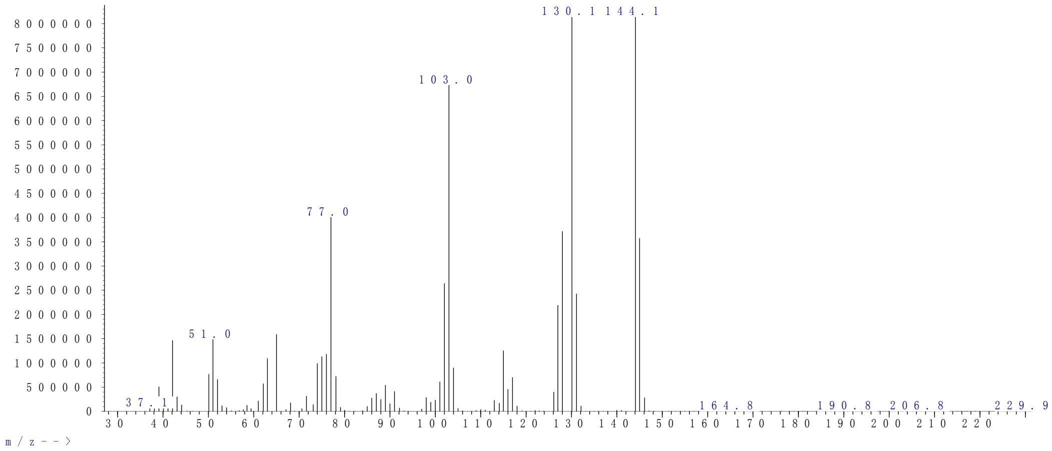

Figure S90. GC-MS spectrum of racemic 4-(4-fluorophenyl)but-3-yn-2-amine (**6c**).

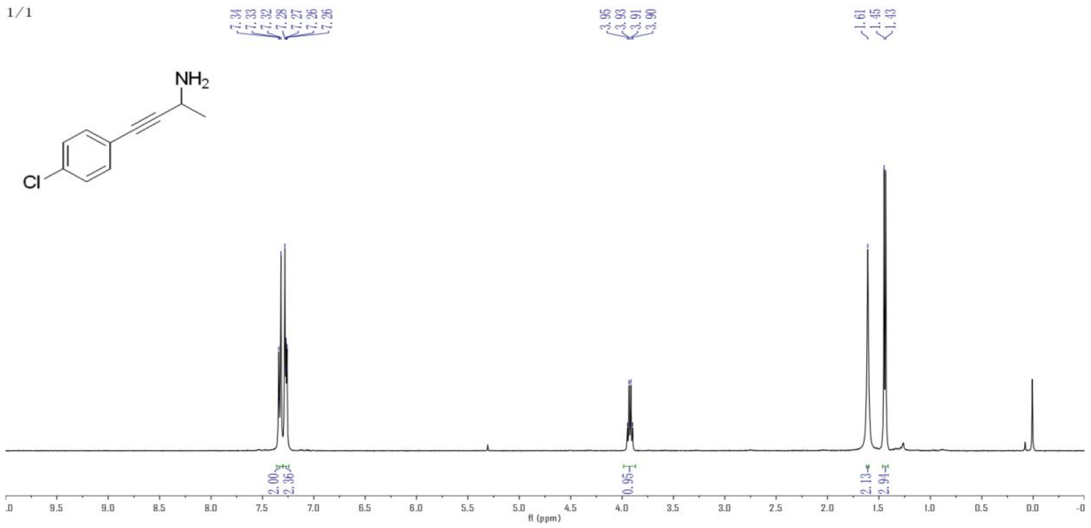

Figure S91. ^{1}H NMR (400 MHz) spectrum of racemic 4-(4-chlorophenyl)but-3-yn-2-amine (**7c**) in $CDCl_3$.

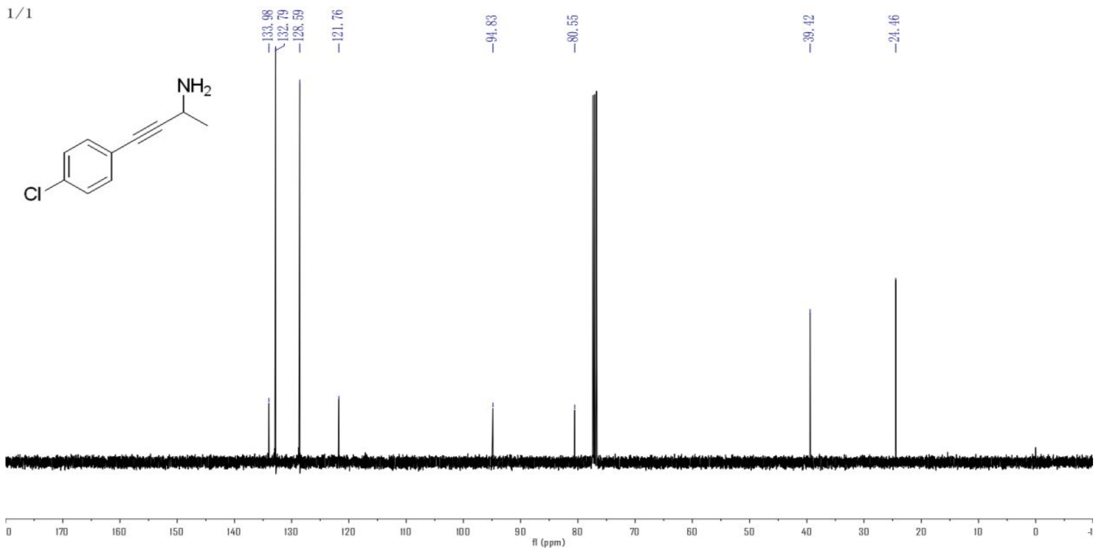

Figure S92. ^{13}C NMR (100 MHz) spectrum of racemic 4-(4-chlorophenyl)but-3-yn-2-amine (**7c**) in $CDCl_3$.

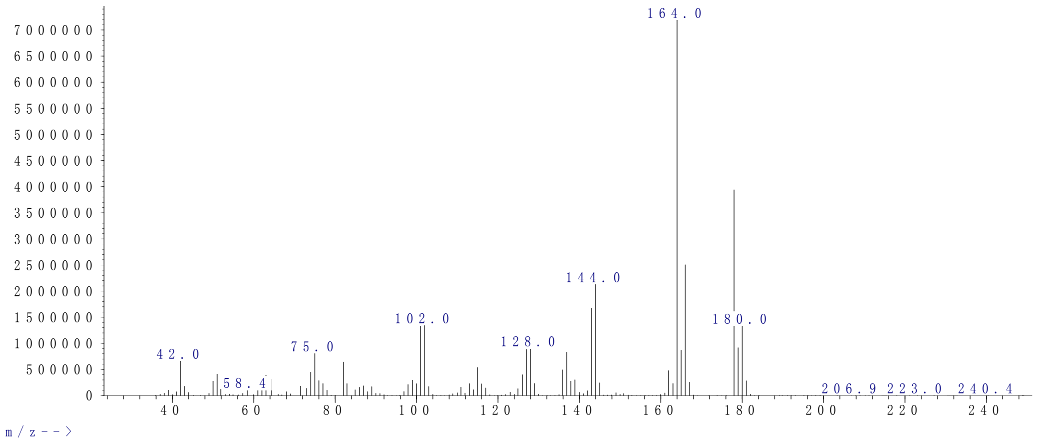

Figure S93. GC-MS spectrum of racemic 4-(4-chlorophenyl)but-3-yn-2-amine (**7c**).

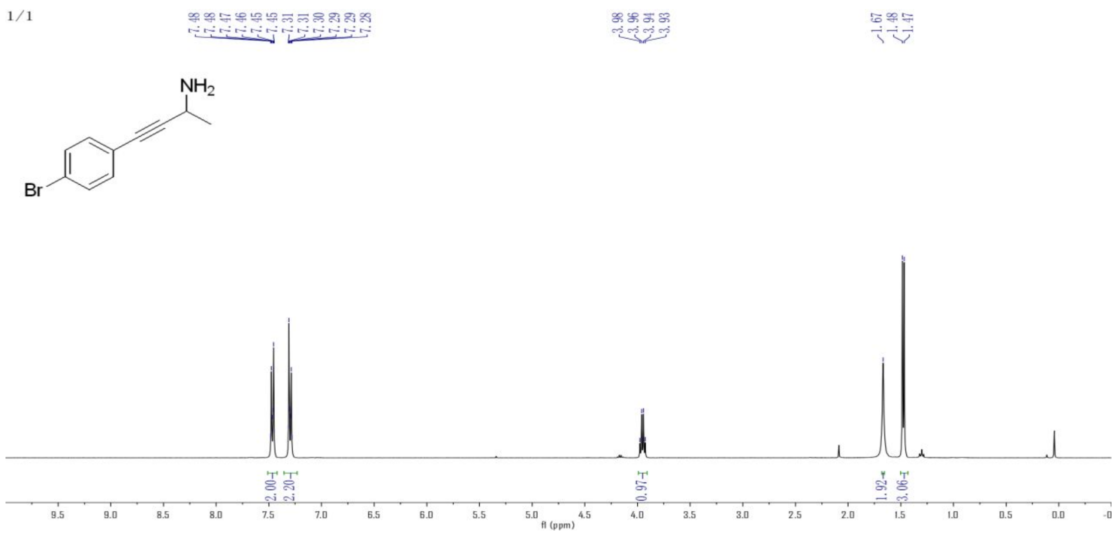

Figure S94. ^{1}H NMR (400 MHz) spectrum of racemic 4-(4-bromophenyl)but-3-yn-2-amine (**8c**) in $CDCl_3$.

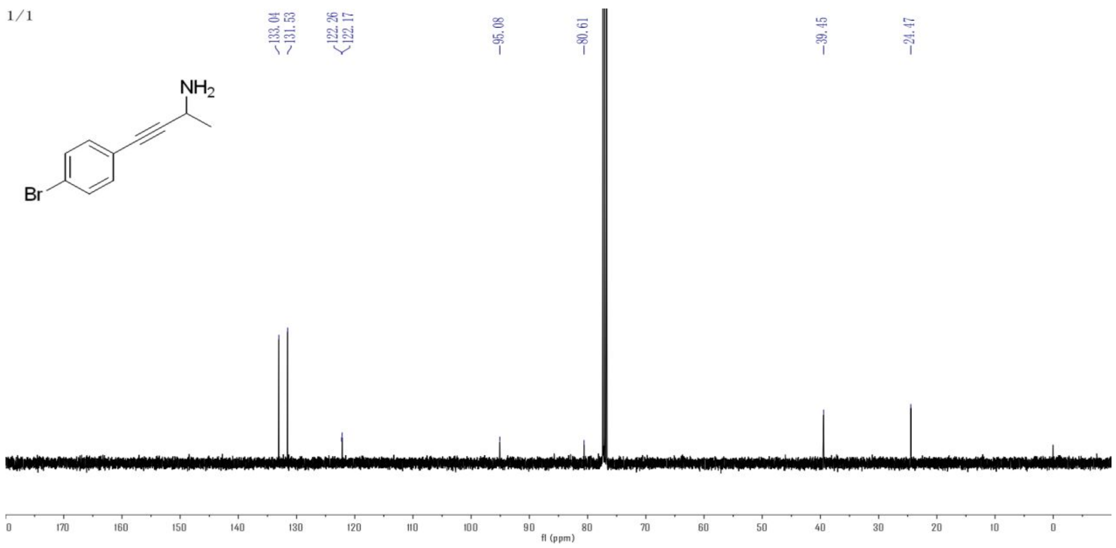

Figure S95. ^{13}C NMR (100 MHz) spectrum of racemic 4-(4-bromophenyl)but-3-yn-2-amine (**8c**) in $CDCl_3$.

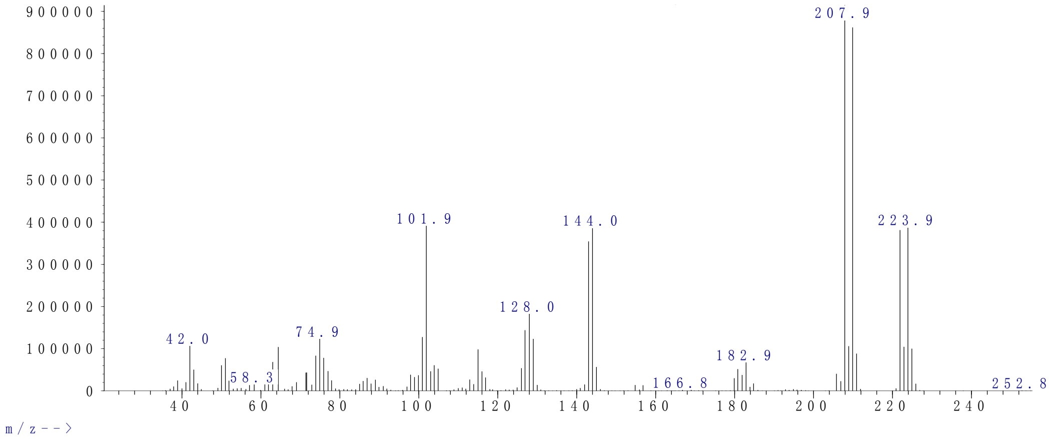

Figure S96. GC-MS spectrum of racemic 4-(4-bromophenyl)but-3-yn-2-amine (**8c**).

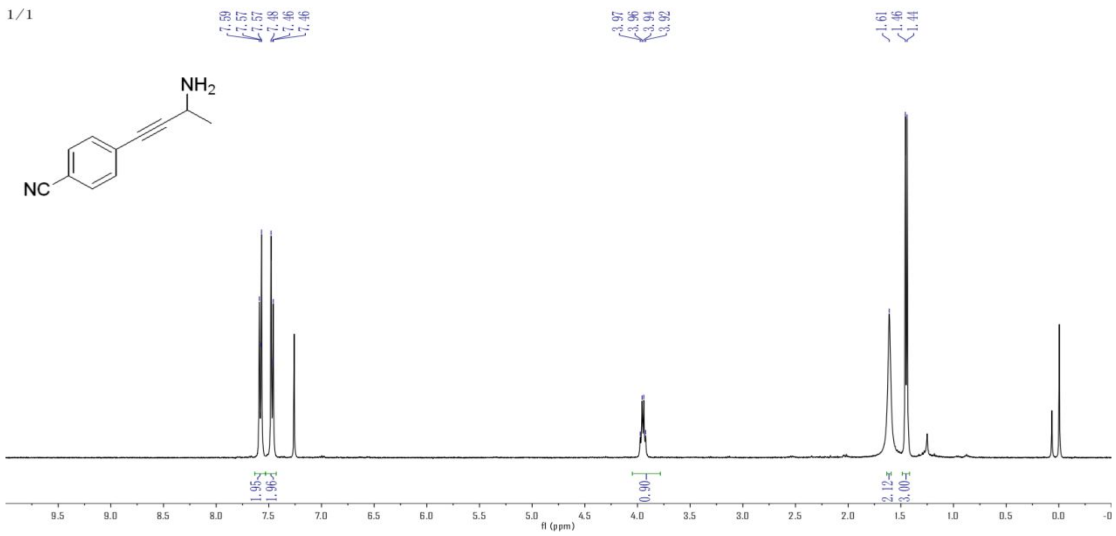

Figure S97. ^{1}H NMR (400 MHz) spectrum of racemic 4-(3-aminobut-1-yn-1-yl)benzonitrile (**11c**) in $CDCl_3$.

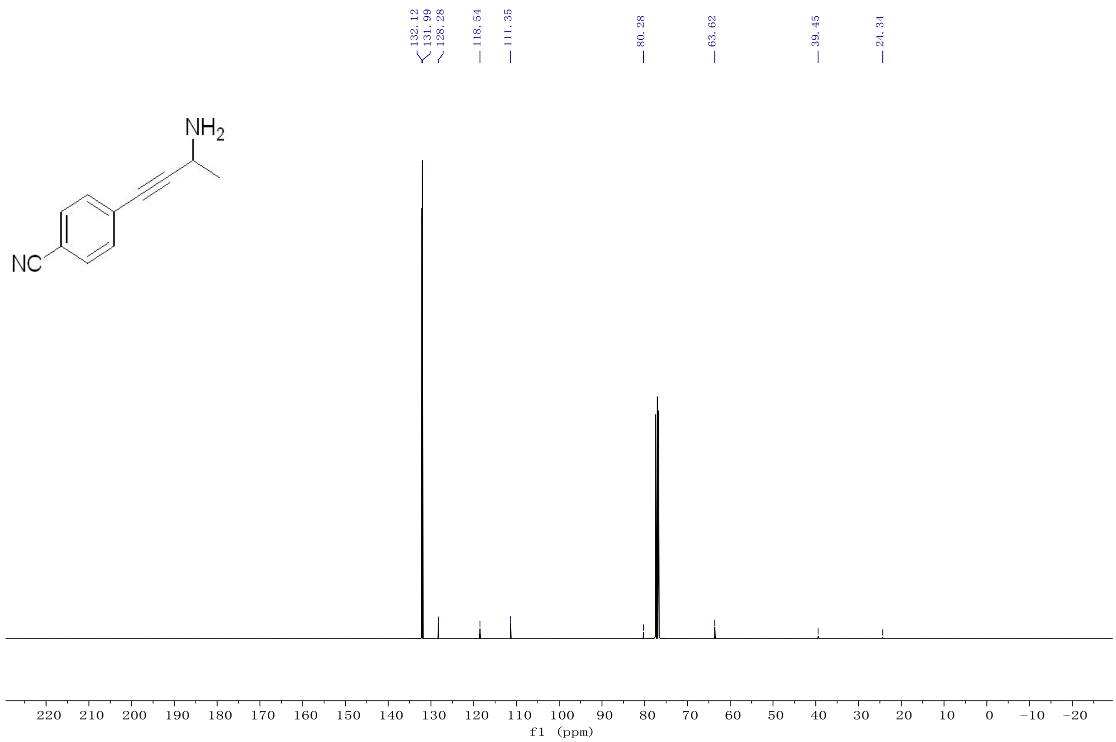

Figure S98. ^{13}C NMR (100 MHz) spectrum of racemic 4-(3-aminobut-1-yn-1-yl)benzonitrile (**11c**) in $CDCl_3$.

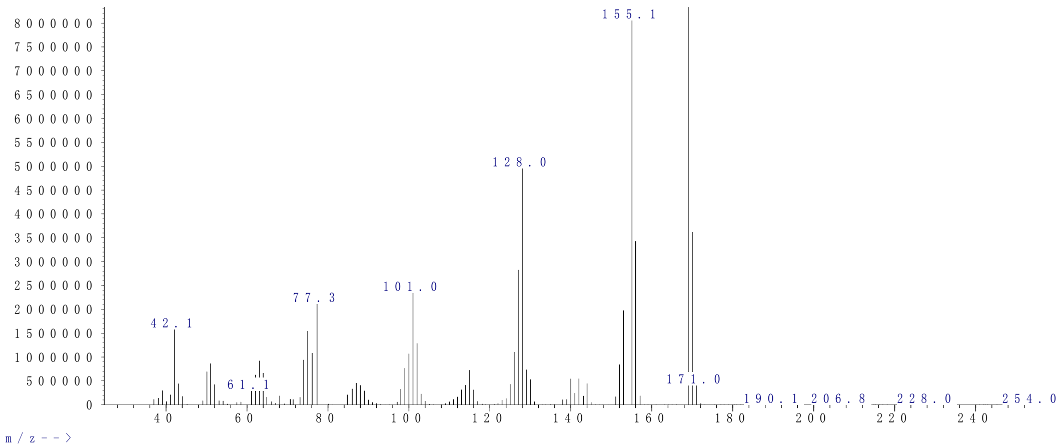

Figure S99. GC-MS spectrum of racemic 4-(3-aminobut-1-yn-1-yl)benzonitrile (**11c**).

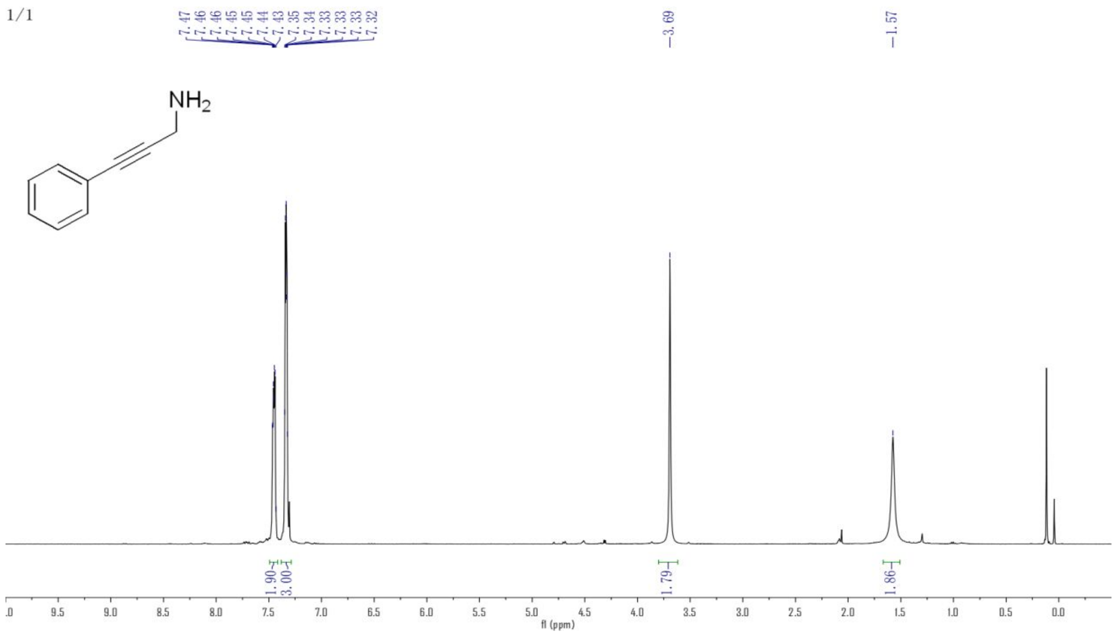

Figure S100. ^1H NMR (400 MHz) spectrum of racemic 3-Phenylprop-2-yn-1-amine (**15c**) in $CDCl_3$.

4-(4-fluorophenyl)but-3-yn-2-amine

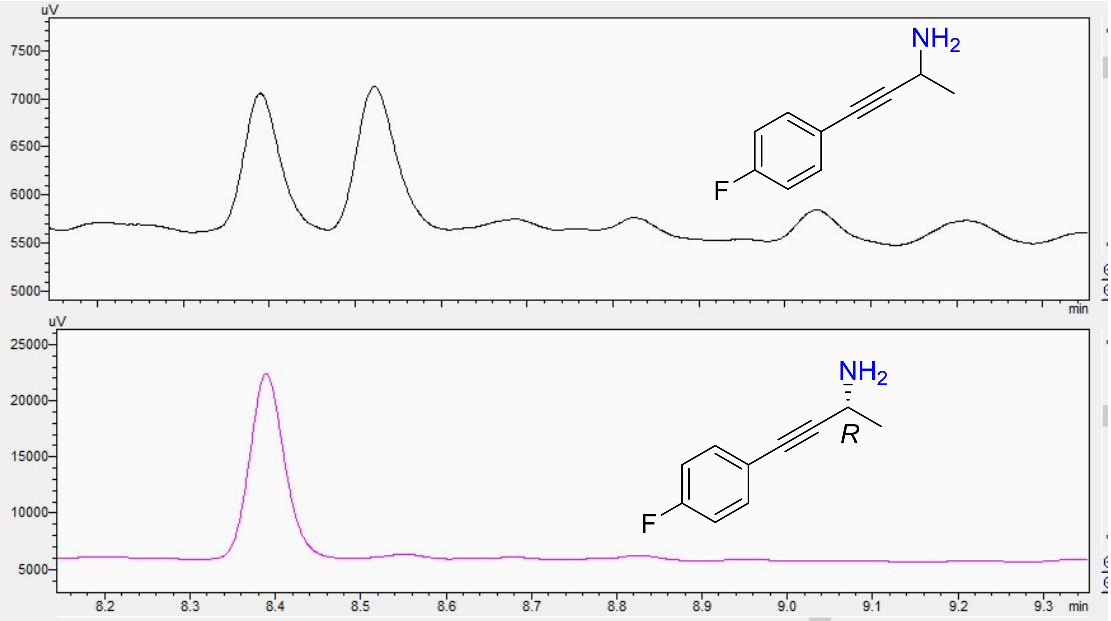

Figure S101. Representative GC chromatogram of racemic 4-(4-fluorophenyl)but-3-yn-2-amine and synthesized (*R*)-**1c**, respectively.

4-phenylbut-3-yn-2-amine

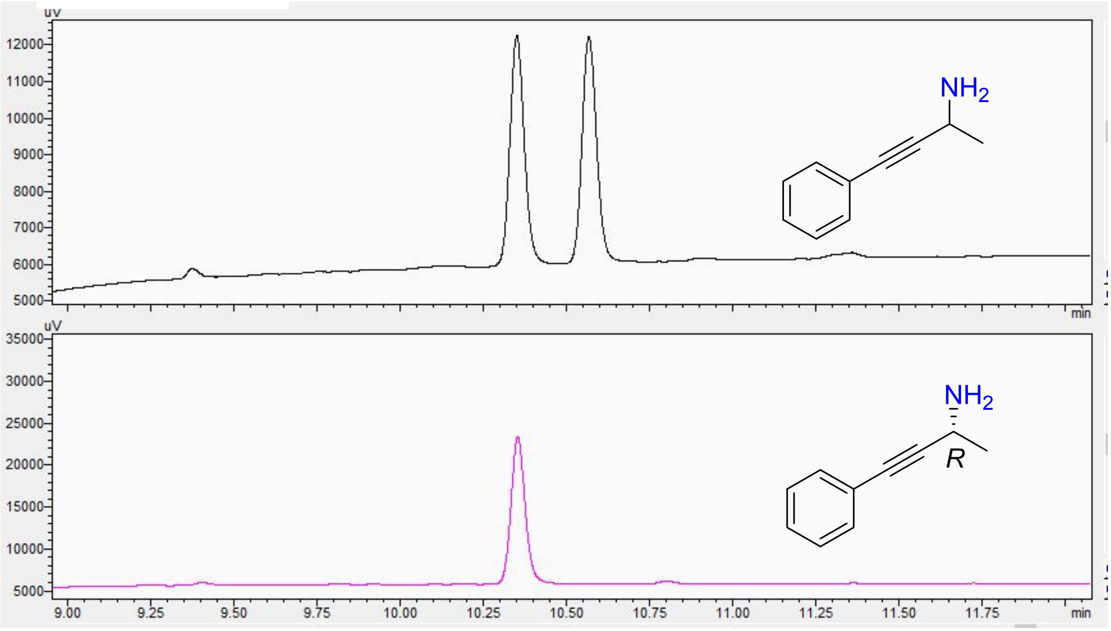

Figure S102. Representative GC chromatogram of racemic 4-phenylbut-3-yn-2-amine and synthesized (*R*)-**6c**, respectively.

4-(4-chlorophenyl)but-3-yn-2-amine

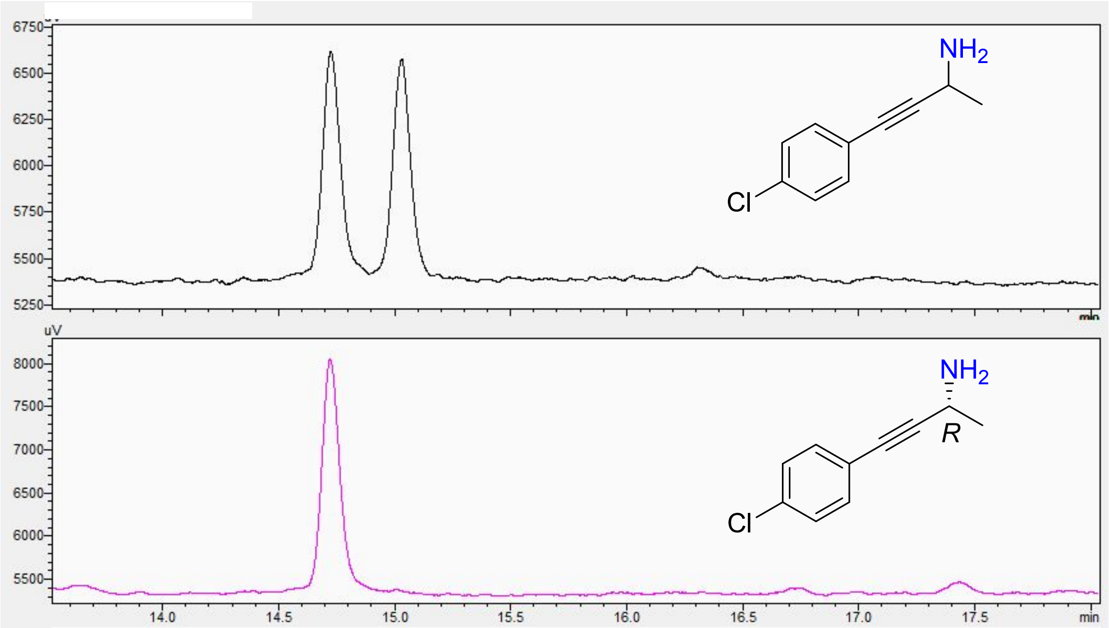

Figure S103. Representative GC chromatogram of racemic 4-(4-chlorophenyl)but-3-yn-2-amine and synthesized (*R*)-**7c**, respectively.

4-(4-bromophenyl)but-3-yn-2-amine

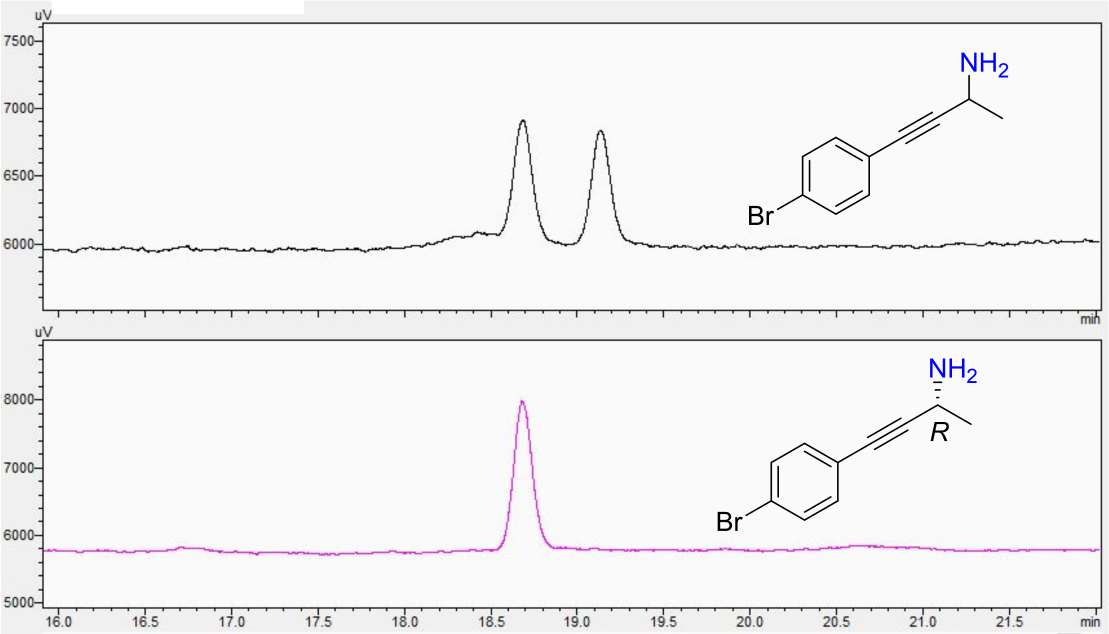

Figure S104. Representative GC chromatogram of racemic 4-(4-bromophenyl)but-3-yn-2-amine and synthesized (*R*)-**8c**, respectively.

4-(4-fluorophenyl)but-3-yn-2-amine

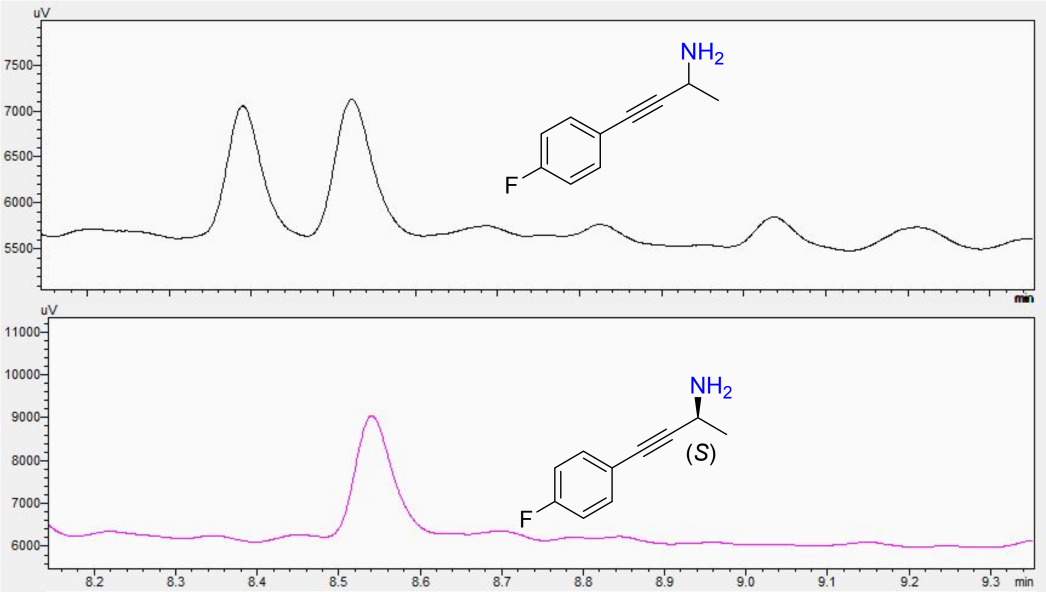

Figure S105. Representative GC chromatogram of racemic 4-(4-fluorophenyl)but-3-yn-2-amine and synthesized (*S*)-**1c**, respectively.

4-phenylbut-3-yn-2-amine

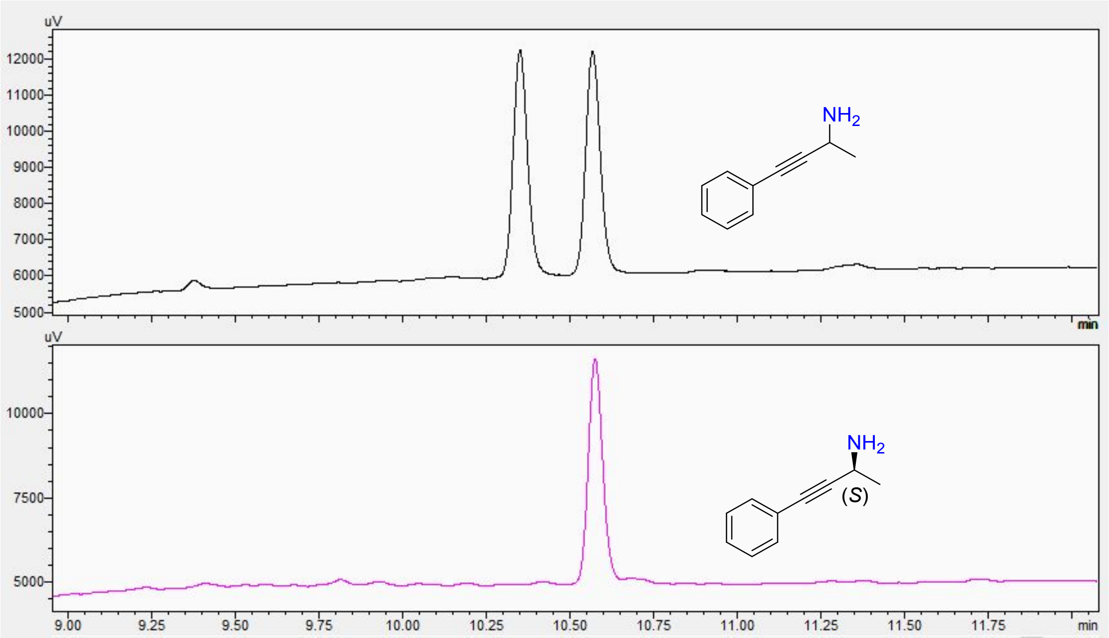

Figure S106. Representative GC chromatogram of racemic 4-phenylbut-3-yn-2-amine and synthesized (*S*)-**6c**, respectively.

4-(4-chlorophenyl)but-3-yn-2-amine

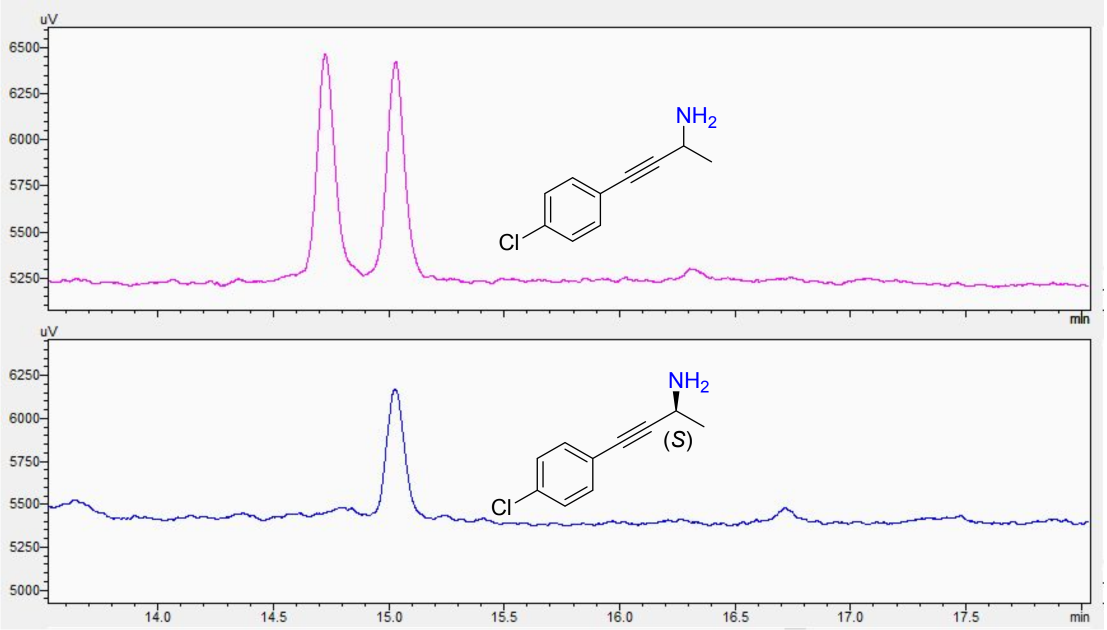

Figure S107. Representative GC chromatogram of racemic 4-(4-chlorophenyl)but-3-yn-2-amine and synthesized (*S*)-**7c**, respectively.

4-(4-bromophenyl)but-3-yn-2-amine

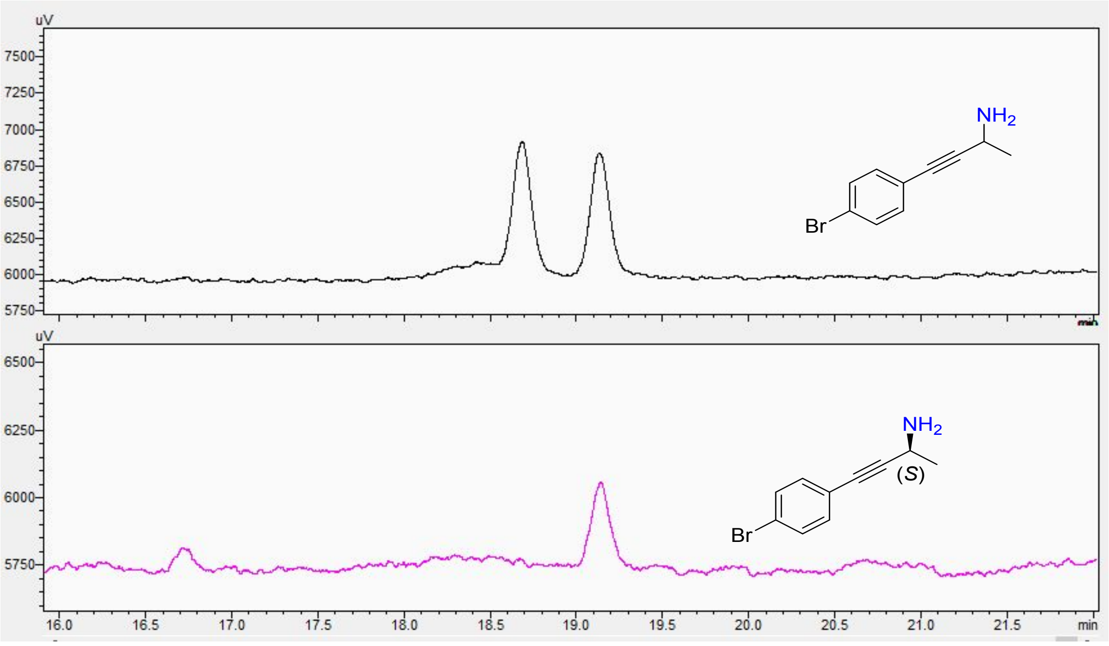

Figure S108. Representative GC chromatogram of racemic 4-(4-bromophenyl)but-3-yn-2-amine and synthesized (*S*)-**8c**, respectively.

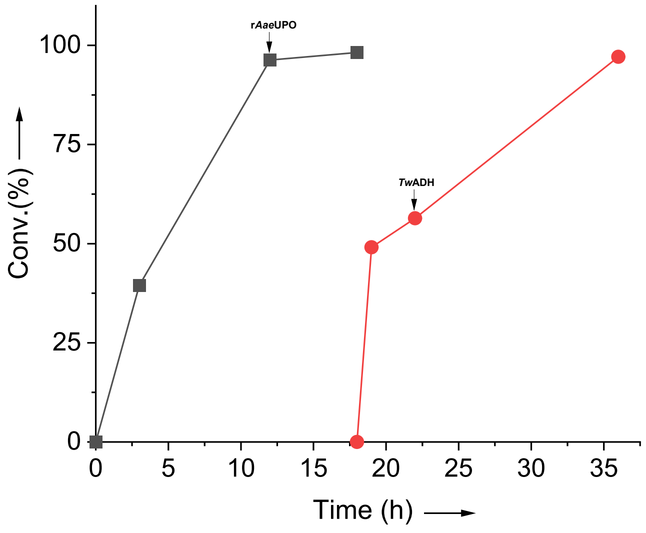

Figure S109. Time course of the preparative scale synthesis of (*S*)-**1a** via the bienzymatic cascade. The formation of ketone **1b** (■) and (*S*)-**1a** (●) was shown over the time course. Fresh r*Aae*UPO and *Tb*ADH were added once during the reactions.

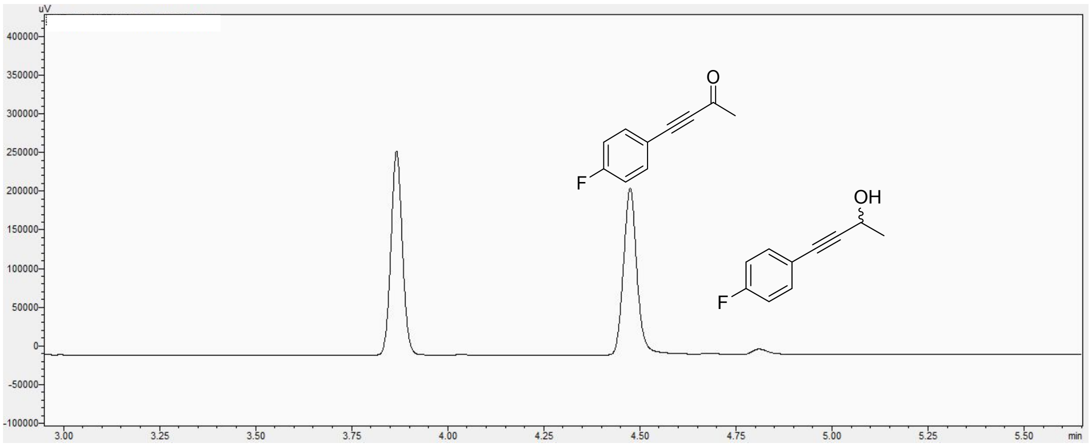

Figure S110. GC chromatogram of **1a** oxidation into **1b** catalyzed by peroxygenase in a preparative scale synthesis.

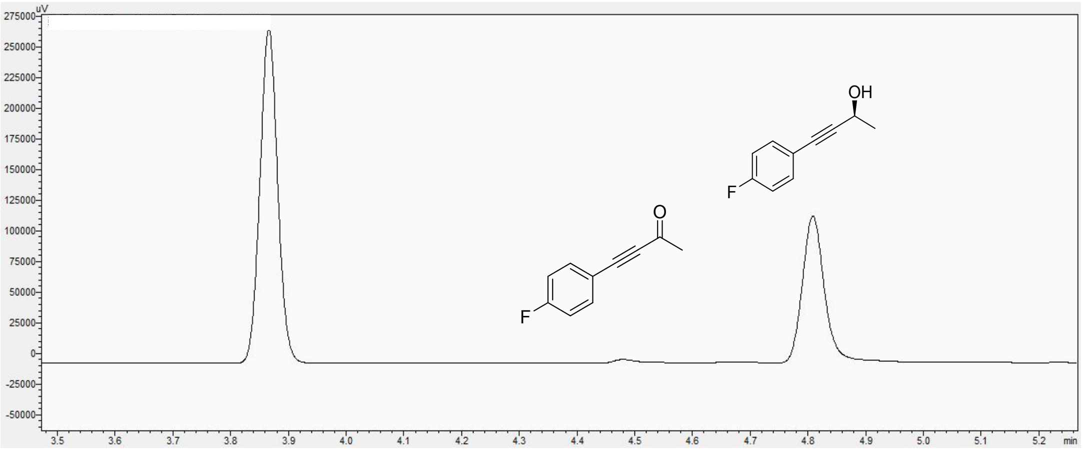

Figure S111. GC chromatogram of **1b** reduction into (*S*)-**1a** catalyzed by *Tb*ADH in a preparative scale synthesis.

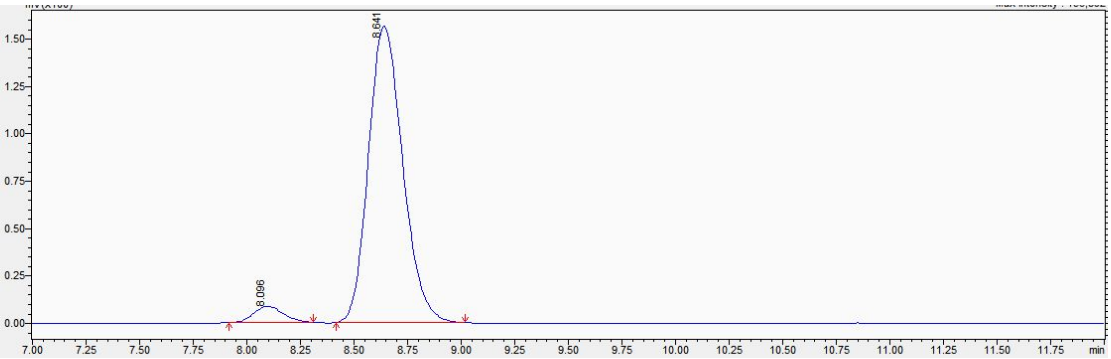

Peak	Retention time (min)	Peak area (mV*min)	Peak height (mV)	Peak area (%)
1	8.096	81432	8626	4.468
2	8.641	1741218	156438	95.532

Figure S112. HPLC chromatogram of **1b** reduction into (*S*)-**1a** catalyzed by *Tb*ADH in a preparative scale synthesis. The ee was 91.1 %.

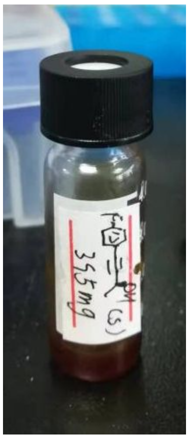

Figure S113. Image of the purified (*S*)-**1a** obtained from the cascade reactions in a preparative scale synthesis.

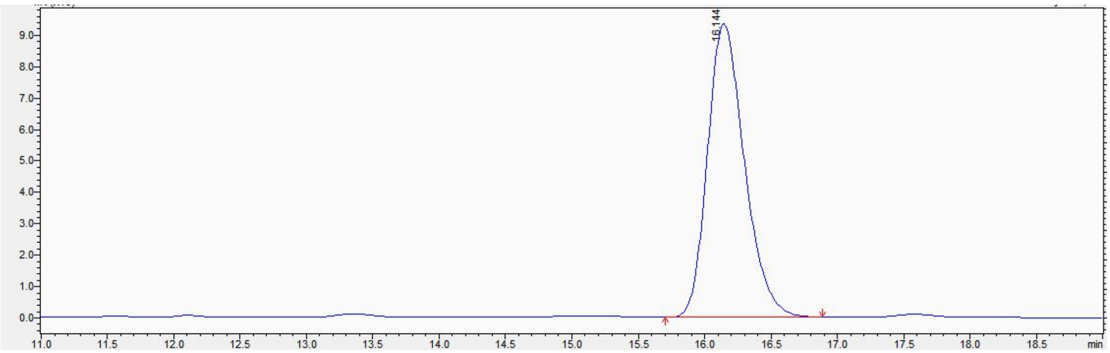

Peak	Retention time (min)	Peak area (mV*min)	Peak height (mV)	Peak area (%)
1	16.144	1810096	93458	100.000

Figure S114. HPLC chromatogram of **1b** reduction into (*R*)-**1c** catalyzed by *At*ATA in a preparative scale synthesis. The ee was >99 %.

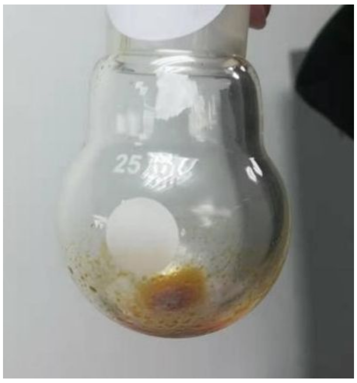

Figure S115. Image of the purified (*R*)-**1c** obtained from the cascade reactions in a preparative scale synthesis.

References

1. Weckbecker, A.; Hummel, W., Cloning, expression, and characterization of an (R)-specific alcohol dehydrogenase from Lactobacillus kefir. *Biocatal. Biotransform.* **2006,** *24* (5), 380-389.

2. Keinan, E.; Hafeli, E. K.; Seth, K. K.; Lamed, R., Thermostable enzymes in organic synthesis. 2. Asymmetric reduction of ketones with alcohol dehydrogenase from Thermoanaerobium brockii. *J. Am. Chem. Soc.* **1986,** *108* (1), 162-169.

3. Schätzle, S.; Steffen-Munsberg, F.; Thontowi, A.; Höhne, M.; Robins, K.; Bornscheuer, U. T., Enzymatic Asymmetric Synthesis of Enantiomerically Pure Aliphatic, Aromatic and Arylaliphatic Amines with (R)-Selective Amine Transaminases. *Adv. Synth. Catal.* **2011,** *353* (13), 2439-2445.

4. Kaulmann, U.; Smithies, K.; Smith, M. E. B.; Hailes, H. C.; Ward, J. M., Substrate spectrum of ω-transaminase from Chromobacterium violaceum DSM30191 and its potential for biocatalysis. *Enzyme Microb. Technol.* **2007,** *41* (5), 628-637.

5. Morris, G. M.; Huey, R.; Lindstrom, W.; Sanner, M. F.; Belew, R. K.; Goodsell, D. S.; Olson, A. J., AutoDock4 and AutoDockTools4: Automated docking with selective receptor flexibility. *J. Comput. Chem.* **2009,** *30* (16), 2785-2791.

6. Panteleev, J.; Huang, R. Y.; Lui, E. K. J.; Lautens, M., Addition of Arylboronic Acids to Arylpropargyl Alcohols en Route to Indenes and Quinolines. *Org. Lett.* **2011,** *13* (19), 5314-5317.

7. Watanabe, K.; Miyazaki, Y.; Okubo, M.; Zhou, B.; Tsuji, H.; Kawatsura, M., Nickel-Catalyzed Asymmetric Propargylic Amination of Propargylic Carbonates Bearing an Internal Alkyne Group. *Org. Lett.* **2018,** *20* (17), 5448-5451.

8. Zhang, X.; Lu, Z.; Fu, C.; Ma, S., Synthesis of highly substituted allylic alcohols by a regio- and stereo-defined CuCl-mediated carbometallation reaction of 3-aryl-substituted secondary propargylic alcohols with Grignard reagents. *Org. Biomol. Chem.* **2009,** *7* (16), 3258-3263.

9. Ying, J.; Le, Z.; Wu, X.-F., Benzene-1,3,5-triyl Triformate (TFBen)-Promoted Palladium-Catalyzed Carbonylative Synthesis of 2-Oxo-2,5-dihydropyrroles from Propargyl Amines. *Org. Lett.* **2020,** *22* (1), 194-198.

10. Liu, Z.; Qin, Z.-Y.; Zhu, L.; Athavale, S. V.; Sengupta, A.; Jia, Z.-J.; Garcia-Borràs, M.; Houk, K. N.; Arnold, F. H., An Enzymatic Platform for Primary Amination of 1-Aryl-2-alkyl Alkynes. *J. Am. Chem. Soc.* **2022,** *144* (1), 80-85.

11. Klauber, E. G.; De, C. K.; Shah, T. K.; Seidel, D., Merging Nucleophilic and Hydrogen Bonding Catalysis: An Anion Binding Approach to the Kinetic Resolution of Propargylic Amines. *J. Am. Chem. Soc.* **2010,** *132* (39), 13624-13626.
